# Calcium Phosphate Bioceramics: A Review of Their History, Structure, Properties, Coating Technologies and Biomedical Applications

**DOI:** 10.3390/ma10040334

**Published:** 2017-03-24

**Authors:** Noam Eliaz, Noah Metoki

**Affiliations:** Biomaterials and Corrosion Lab, Department of Materials Science and Engineering, Tel-Aviv University, Ramat Aviv 6997801, Israel; noametoki@gmail.com

**Keywords:** bioceramics, biomineralization, bone cement, calcium phosphate, coating, composites, drug delivery, electrochemical deposition, functionally graded materials, nano-hydroxyapatite

## Abstract

Calcium phosphate (CaP) bioceramics are widely used in the field of bone regeneration, both in orthopedics and in dentistry, due to their good biocompatibility, osseointegration and osteoconduction. The aim of this article is to review the history, structure, properties and clinical applications of these materials, whether they are in the form of bone cements, paste, scaffolds, or coatings. Major analytical techniques for characterization of CaPs, *in vitro* and *in vivo* tests, and the requirements of the US Food and Drug Administration (FDA) and international standards from CaP coatings on orthopedic and dental endosseous implants, are also summarized, along with the possible effect of sterilization on these materials. CaP coating technologies are summarized, with a focus on electrochemical processes. Theories on the formation of transient precursor phases in biomineralization, the dissolution and reprecipitation as bone of CaPs are discussed. A wide variety of CaPs are presented, from the individual phases to nano-CaP, biphasic and triphasic CaP formulations, composite CaP coatings and cements, functionally graded materials (FGMs), and antibacterial CaPs. We conclude by foreseeing the future of CaPs.

## 1. Historical Perspective

Calcium phosphate (CaP) is the common name of a family of minerals containing calcium cations (Ca^2+^) together with orthophosphate (${\mathrm{PO}}_{4}^{3-}$), metaphosphate (${\mathrm{PO}}_{3}^{-}$), or pyrophosphate ($P_{2}O_{7}^{4-}$) anions, and sometimes hydrogen (H^+^) or hydroxide (OH^−^) ions. CaP is the principal form of calcium found in bovine milk and blood. It is the major inorganic constituent of bone (~60 wt % of bone), and the main constituent of tooth enamel (ca. 90%). Calcium phosphates with a Ca/P atomic ratio between 1.5 and 1.67 are called apatites (e.g., hydroxyapatite or fluorapatite). The term apatite was coined in 1786 by German geologist Abraham Gottlob Werner based on the ancient Greek word “apatao”, which means “to mislead”.

The history of CaPs has been reviewed in various articles (see, for example, [1,2,3,4]). The first articles to describe the structure and composition of bones, teeth and other types of calcified tissues appeared in the last quarter of the 17th century (see, for example [5,6]). In 1769, the Swedish chemist and metallurgist Johan Gottlieb Gahn discovered the existence of CaP in bones. However, only since this fact was reported by the German-Swedish pharmaceutical chemist Carl Wilhelm Scheele in 1771, has phosphorus been produced from bone ash [7]. In the last decade of the 18th century, the French chemists Antoine François Comte de Fourcroy and Nicolas Louis Vauquelin discovered the existence of acidic CaP, nowadays known as monocalcium phosphate monohydrate (MCPM), monocalcium phosphate anhydrous (MCPA), dicalcium phosphate anhydrous (DCPA, monetite), and dicalcium phosphate dihydrate (DCPD, brushite). The organic-inorganic composite nature of bone has been known since at least 1788 [1].

Various processes for preparation of calcium-deficient hydroxyapatite (CDHA) were developed by 1807 [8]. In 1808, Nicholson already reported [9] the considerable compositional differences between enamel, dentin, and bones. The presence of carbonates, fluorides, chlorides, magnesium, and sodium in the mineral phase of bones was already known at that time. In 1809, the structure, composition, properties and formation mechanisms of bones were described in details [10]. The general principles of bone and teeth formation (biomineralization) were established by 1814 [11]. By 1827, the German mineralogist Gustav Rose established the correct understanding of the chemical composition of apatites [1]. In 1832, the chemical term “tribasic phosphate of lime” was introduced, which corresponds to the currently known α-tricalcium phosphate (TCP) and β-TCP [12]. In 1843 John Percy presumably reported the first synthesis of octacalcium phosphate (OCP), as he referred to both “basic water” (which implies existence of HPO_4_) and five molecules of hydrate water [13]. The presence of CaPs in corals was found in 1846 [14]. The first solubility tests of CaPs were reported in 1847 [15]. The first academic thesis on CaP was published in 1853 [16]. In the second half of the 19th century, CaPs were extensively investigated as fertilizers. In addition, the compositional differences between bones in young and old individuals were investigated [17].

Attempts to treat various diseases with CaPs date back to 1797, initially to treat rickets (rachitis) [1]. In the 19th century, the first well-documented studies on autografts and allografts were published. The German surgeon and ophthalmologist Philipp Franz von Walther replaced surgically removed parts of a skull after trepanation with a bone autograft [18]. Sixty years later, the Scottish surgeon Sir William Macewen successfully reconstructed an infected humerus of a four-year-old boy by a bone allograft obtained from the tibia of a child with rickets [19]. Junius Cravens mixed CaP powder with lactic acid and applied it onto an exposed pulp tissue [20]. This pulp-capping agent was marketed by the S.S. White Company (Lakewood, NJ, USA), with the tradename “Lacto-Phosphate of Lime”. This may be considered as the first report on artificial CaP-based biocomposites and hybrid biomaterials [1].

In 1906, the earliest structural drawing of an ion-substituted molecule of CaP, nowadays known as carbonate apatite, was published [21]. The first attempt to implant a laboratory-made CaP (specifically, TCP) as an artificial material to repair surgically created fractures in rabbit bones was made in 1920 by the American surgeon Fred Houdlette Albee [22]. A much more rapid bone growth and union was observed when TCP was injected into the gap between the bone ends than in the controls. Five years earlier, Albee invented bone grafting [23]. The crystal structure of apatites was reported in the early 1930s [24,25,26]. In parallel, first studies of the crystal dimensions of biological apatites were reported [27,28,29]. In the second quarter of the 20th century, the solubility of apatites and other CaPs was studied extensively. In the early 1930s, differentiation was made between α- and β-TCP, and the early versions of the high-temperature diagram for the binary system CaO–P_2_O_5_ were proposed [30,31]. The term “osteoinduction” refers to the process by which osteogenesis is induced. It is a phenomenon regularly seen in any type of bone healing process. In a bone healing situation such as a fracture, the majority of bone healing is dependent on osteoinduction [32]. The classic osteoinductive phenomenon was defined in 1931 [33]. In 1940, a fundamental study on the equilibrium in the system CaO–P_2_O_5_–H_2_O was published [34]. In 1948, it was established that only certain types of CaPs influence the bone-healing process [35]. In 1950, distinct Ca/P borders were drawn between OCP and hydroxyapatite (HAp) [36]. Year 1950 also marks the beginning of self-setting CaP formulations [37]. However, their first biomedical application is attributed to Köster et al. [38].

In 1952, Per-Ingvar Brånemark, a Swedish physician and research professor, coined an important term—osseointegration, which derives from the Greek *osteon* (bone) and the Latin *integrare* [39]. Brånemark conducted an experiment where he utilized a titanium implant optic chamber to study blood flow in rabbit bone. When the experiment ended, he discovered that the bone had integrated so completely with the implant that the chamber could not be removed. Osseointegration was originally defined as a direct structural and functional connection between ordered living bone and the surface of a load-carrying implant. However, nowadays an implant is regarded as osseointegrated when there is no progressive relative movement between the implant and the bone with which it has direct contact. Osseointegration is also defined as the formation of a direct interface between an implant and bone, without intervening soft (fibrous) tissue. In 1965 Brånemark placed dental implants into the first human patient, Gösta Larsson, who had a cleft palate defect and required implants to support a palatal obturator. Today, Brånemark is considered as “the father of modern dental implantology”.

Amorphous calcium phosphate (ACP) was first described by Aaron Posner et al. in the mid-1960s [40,41,42]. The smallest construction block in the apatite structure that he suggested is the so-called “Posner’s cluster”, Ca_9_(PO_4_)_6_. In 1969, hot-pressed HAp powder into dense and useful shapes was reported [43]. This may be the earliest article on the fabrication of CaP implants. In 1971, the first study on preparation of biodegradable porous β-TCP scaffolds was published [44]. In 1973, the first study on preparation and implantation of resorbable and porous CaP (specifically, β-TCP) was published [45]. In 1975, the modern dental application of CaP began: β-TCP was applied both in surgically created periodontal defects [46] and as an adjunct to apical closure in pulpless permanent teeth [47]. In 1979, dense HAp cylinders were used for immediate tooth root replacement [48].

The history of CaP coatings, films and layers [49] started in 1976 [50], while that of CaP-based biocomposites and hybrid biomaterials started in 1981 [51]. In the early 1980s, the dental community began using HAp blocks and coatings to augment bone to encourage fixation in restorative dental procedures; the chemical stability and excellent biocompatibility of HAp made it an attractive material of choice. Subsequently, the orthopedic community began using HAp for bone defect augmentation, and as an implant coating [4]. A rapid commercialization of CaP (mainly, HAp) bioceramics in the dental and orthopedic markets occurred in the 1980s, and was led by Jarcho in the USA [52], de Groot in Europe [53], and Aoki in Japan [54]. The first HAp-coated primary hip prosthesis in humans was implanted in 1985 by Furlong [55]. His femoral implant was fully coated with a 200 μm thick layer of bioceramic. In 1985, drug-loaded CaP bioceramics was reported for the first time [56]. The history of nano-CaP started in 1994 [57,58,59,60]. In the same year, the use of CaP as scaffolds began [61], while applications of CaP bioceramics in tissue engineering began in 1998 [62,63].

Many books have been published over the years on CaP bioceramics. References [49,53,64,65,66,67,68,69,70,71,72,73] are few examples.

## 2. The Structure, Chemistry and Mechanical Properties of Bone

The structure, chemistry and mechanical properties of natural bone have been reviewed in numerous articles (see, for example, [74,75,76,77,78,79,80,81,82,83,84,85,86,87,88]).

**Figure 1:**
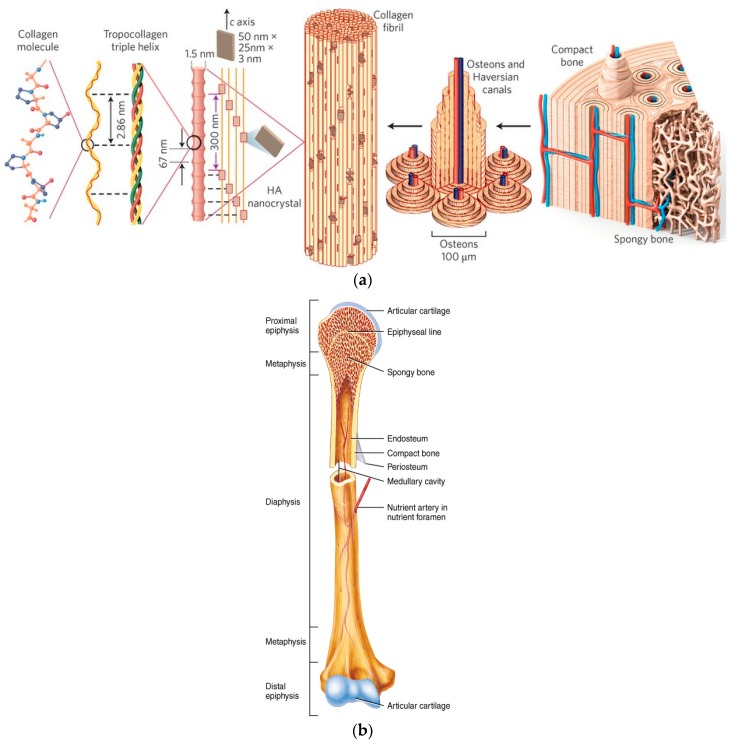
(**a**) Hierarchical structure of bone [88]. Reproduced with permission from Nature Publishing Group; (**b**) Typical structure of long bone [91]. Reproduced from Tortora and Derrickson, *Principles of Anatomy and Physiology*, 11th edition, © John Wiley & Sons, Inc.

Bone is the basic unit of the human skeletal system. Bone provides the framework for and bears the weight of the body, protects the vital organs, supports mechanical movement, hosts hematopoietic cells, and maintains iron homeostasis. Bone has a complex, varying arrangement of structures on broad length scales (Figure 1a), which together enables diverse mechanical, biological and chemical functions. It is a hierarchical, complex, functionally graded material (FGM), with inner cancellous and outer cortical bone. In addition, according to Wolff’s law [89,90], bone in a healthy person or animal will adapt to the loads under which it is placed. If loading on the bone increases, it will remodel itself over time to become stronger by first changing the internal architecture of the trabeculae, and then thickening the external cortical portion of the bone. In contrast, the loading on the bone decreases, the bone will become less dense (a process known as osteopenia) due to the lack of the stimulus required for continued remodelling. This might occur, for example, after insertion of an artificial joint (e.g., total hip replacement), due to stress shielding that results from the higher rigidity (Young’s modulus of elasticity) of the metal compared to bone.

It is important to understand the structural relationship between the various levels of hierarchical structural organization in order to understand the function of HAp within it. These are: (1) the macrostructure: cancellous versus cortical bone; (2) the microstructure and sub-microstructure: Haversian canal, osteons, lamellae; (3) the nanostructure: fibrillar collagen; and (4) the sub-nanostructure: molecular constituents of the mineral, collagen, and non-collagenous organic proteins.

On the macroscopic level, bones can have diverse shapes depending on their respective function [81]. Yet, bones are usually categorized into two types: cortical (or compact) bone, and cancellous (or trabecular) bone, see Figure 1b. Cortical bone forms the outer shell of most bones. These can reach a thickness of between several tenths of a millimetre (in vertebra) to several millimetres or even centimetres (in the mid-shaft of long bones). It is a fairly dense bone, with porosity in the order of 6% [81]. Cancellous bone usually forms inside of bones that are under compressive stress. The interconnecting framework of trabeculae is in a number of combinations, all following basic cellular structures: rod-rod, rod-plate, or plate-plate [75]. Trabecular bone has pores in the order of 80% [81]. The typical thickness of trabeculae is about 50–300 μm, with an orientation that depends on the load distribution in the bone [75]. When conducting *in vivo* study of osseointegration of uncoated and coated implants, one should take into account the reactivity of bone surrounding the implant. Namely, at the diaphysis (Figure 1b), native bone is in close contact with the implant. The metaphysis (Figure 1b), on the other hand, contains cancellous bone and is more reactive (e.g., it usually provides faster facture healing) [92].

In the microstructure of bone, mineralized collagen fibres form into planar arrangements called lamellae (3–7 μm wide) [75]. As seen in Figure 1a, in some cortical bone the lamellae wrap in concentric layers (3–8 lamellae) around a central canal, to form an osteon or a Harversian system. The osteons look like cylinders, and they are roughly parallel to the long axis of the bone. Other forms of cortical bone in which no such pattern can be distinguished are called woven bone [75]. On the other hand, the microstructure of trabecular bone has a different arrangement. It corresponds to a fibre texture, where all the mineral platelets are arranged parallel to a common direction (corresponding to the fibre direction of the collagen). This common direction exhibits some distribution and is defined roughly within ±30° [81].

In the nanostructure of the lamellae there are mineralized collagen fibril of about 100 nm in diameter. This is the basic building block of the bone material. The fibrils consist of an assembly of 300 nm long and 1.5 nm thick collagen molecules. The collagen (type I) is the primary organic component of the matrix. The collagen molecules are secreted by osteoblasts, and are then self-assembled. Apatite crystals occur within the discrete spaces in the collagen fibrils. The collagen in the fibrils limits the possible primary growth of the crystals, forcing them to be discrete and discontinuous. Crystals occur at regular intervals along the fibrils, with an approximate repeat distance of 67 nm [93], which corresponds to the distance by which adjacent collagen molecules are staggered. It is important to note that both the arrangement of the lamellae and the collagen fibres up to the nanometre scale enhance the isotropic properties found in bone, hinder crack propagation, and increases toughness [94].

The formation of the apatite in the extracellular space of the collagen is called “biomineralization”. It is important to note that the process differs depending on different factors such as stage (e.g., development, fracture healing), region, age, etc. The nucleation process in the bones is associated with interaction between anionic proteins and type I collagen fibrils that may provide the stereochemical orientation of negatively charged groups that is sufficient for HAp nucleation. Once the bone matrix is formed, a characteristic time course of 13 days will take place before the matrix starts to mineralize rapidly. This process is called primary mineralization, and within a few days, the matrix will mineralize up to 70%. The residual 30% of increase in mineral content lasts several years, and is called secondary mineralization [78]. Epitaxial considerations have been found to be of primary importance in biological mineralization, in understanding the formation of teeth and bones, as well as in pathological processes such as the development of urinary calculi [95].

With respect to the shape of bone mineral crystals, the majority of the studies describe them as plate-like in shape [78], but with a rather wide range of dimensions; the thickness of the platelets ranges from 1.5 to 9 nm, the length from 15 to 200 nm, and the width from 10 to 120 nm [96,97,98,99,100]. The apatite crystals are typically planar with respect to the *a*–*c* plane [98]. Their *c*-axis in a cortical bone is generally parallel to the bone axis [99], i.e., roughly parallel to the long axes of the collagen fibrils [101]. The size and shape of bone apatite crystals change with species, age, and disease state. For bone mineralization in a given species, the average crystal size is smallest at formation and increases to maturity, at which time there is a levelling of this growth process. Moreover, a single specimen contains a range of particle sizes and shapes, and different measurement techniques may yield different average values on polydisperse samples [97]. The conditions in the human body apparently limit the growth of HAp *in vivo*. The most effective inhibitors seem to be polyanions, particularly polyphosphates or polyphosphonates. Salivary peptides and proteins, such as statherin and praline-rich proteins (PRPs), respectively, are powerful inhibitors. These macromolecules appear to prevent the precipitation of CaP phases in saliva in spite of the supersaturation of this secretion with respect to HAp. The inhibiting mechanism was related to their adsorption onto the surface of apatite seeds [102]. Proteoglycans, even at low concentrations, can delay or prevent apatite formation. On the other hand, bone collagen is considered to be intimately involved in the nucleation of bone mineral [97]. It should also be noted that some investigations found needle-like or rod-like crystals [103,104], which has led to a long ongoing debate about the nature of the mineral particle shape. It was argued that the so-called rod-like or needle-like shape resulted from either a special observation angle of the crystals or a transformation caused by heat treatment [105,106,107,108]. A predominant needle-like impression was related to the side-on view of the particles, which has the strongest absorption contrast in the transmission electron microscope (TEM) [78]. After more differentiated image analysis, platelets with on-top view were identified as well [109]. An explanation for a plate-like mineral shape may be that crystal growth occurs via an OCP intermediate. OCP has nearly the same crystal structure as HAp, but contains an extra hydrated layer that may be responsible for the observed plate-shaped crystals in natural bone [110].

Due to the nanocrystalline nature of apatite in the bone, various diffraction techniques have not yet given much information on the fine structural details related to apatite nanocrystals. That is because assemblies of nanoparticles give only broad diffraction patterns, similar to ones from an amorphous material [96,111]. Knowledge of the crystal structure of biological apatites has been limited because of the absence of suitable single crystals for study [66]. Nevertheless, it was reported that the isolated crystals from natural bones were poorly crystalline apatite, similar to powdered intact bone from which they were originated [107]. Two different crystallographic structures have been proposed for biological apatites [40,66,112,113,114,115]: (1) hexagonal (not close packed), space group *P*6_3_/*m*, with lattice parameters *a* = *b* = 9.432 Å, *c* = 6.881 Å [112], and (2) monoclinic with lattice parameters *a* = 9.421 Å, *b* = 2*a*, *c* = 6.881 Å, γ = 120° [113]. These two structures share the same elements, with a stoichiometric Ca/P atom ratio of 1.67. The major difference between them is the orientation of the hydroxyl groups. In the hexagonal HAp, two adjacent hydroxyl groups point at the reverse direction, while in the monoclinic form—hydroxyl groups have the same direction in the same column, and an opposite direction among columns [114].

The chemical composition of bone is given in Table 1. Biological apatites deviate from the stoichiometric composition of HAp, Ca_10_(PO_4_)_6_(OH)_2_, and contain significant amounts of ion substitution impurities such as $Na^{+}$, $Mg^{2+}$, $K^{+}$, citrate, $HPO_{4}^{2-}$, carbonate ($CO_{3}^{2-}$), $Cl^{-}$, $F^{-}$, etc. [116]. Since the HAp bathes in aqueous biological fluids, the types and extent of these substitutions change over time [117]. In analysing the mineral substance by different techniques, such as Fourier transform infrared (FTIR) spectroscopy, some models even suggest that the nanocrystalline apatite is covered with a hydrated layer containing ions, such as Ca^2+^, ${\mathrm{HPO}}_{4}^{2-}$, ${\mathrm{CO}}_{3}^{2-}$, in different sites of the crystal, and can be considered as either OCP or DCPD phase. In most reports, the Ca/P atom ratio of biological apatite has been either lower than or close to that of stoichiometric HAp (Ca/P = 1.67) [66,118]. However, Ca/P > 1.67 has been reported too [103,106,119]. The unique chemical composition of biological apatite is reflected by: (1) the lack of anticipatory hydroxyl group, and (2) the existence of ${\mathrm{HPO}}_{4}^{2-}$ [84]. For hydroxyl group, it was reported that only a few percentage of the predicated concentration was detected in bone [120]. The presence of ${\mathrm{HPO}}_{4}^{2-}$ is ascribed to either ionic substitution, or to hydrolysis of ${\mathrm{PO}}_{4}^{3-}$ [84].

The resolution and chemical sensitivity of modern analytical techniques such as electron energy loss spectroscopy (EELS) and energy dispersive X-ray spectroscopy (EDS) in scanning transmission electron microscopy (STEM) are greatly limited by the susceptibility of biological minerals to electron-beam damage. Moreover, many physiological ions with low atomic number have unfavourable spectroscopic properties that can make quantification very difficult or impossible [122]. To overcome this limitation, Joester et al. have used atom probe tomography (APT) and showed that this advanced technique is well suited for distinguishing between geological fluorapatite and synthetic single crystals of fluorapatite, chlorapatite and HAp, based on their spectrometric fingerprint. Spectral analysis was then expanded to vertebrate bone and dentin as examples for apatite-mineralized tissues that contain a range of inorganic substituents and organic molecules. Finally, preliminary data demonstrated that APT captures the fibrous nature of the collagenous organic matrix and reveals additional detail regarding the chemical nanostructure of homophase and heterophase interfaces [122].

Bone cells are embedded in the solid matrix of this connective tissue. Bone has four major types of cells: osteoblasts, osteocytes, osteoclasts, and bone lining cells. Osteoblasts are bone forming cells that have only one nucleus. They are located along the bone surface and comprise 4%–6% of the total resident bone cells. The osteoblasts originate from the differentiation of osteogenic cells in the tissue that covers the outside of the bone, or the periosteum and the bone marrow. The synthesis of bone matrix by osteoblasts occurs in two main steps: deposition of organic matrix and its subsequent mineralization [123]. Once the osteoblast is finished working it is actually trapped inside of the bone once it hardens. When the osteoblast becomes trapped, it becomes known as an osteocyte. Thus, osteocytes are mature bone cells. They are dispersed in the bone matrix and supposedly act as stress sensors. Other osteoblasts remain on the top of the new bone and are used to protect the underlying bone, these have become known as lining cells. Bone lining cells have flat organelles so they can easily cover the bone without interfering with other cells functions. Osteoclasts are very large multinucleate cells that are responsible for the breakdown of bones (namely, matrix resorption).

**Figure 2:**
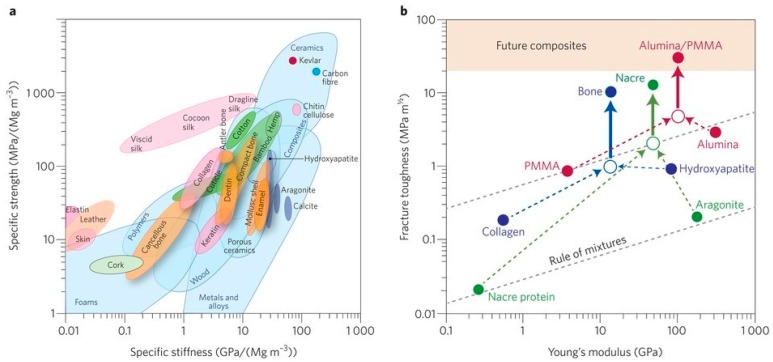
(**a**) Ashby chart of strength versus Young’s modules of elasticity (specific values) for natural and synthetic materials. Note values for collagen, hydroxyapatite (HAp), cancellous bone, compact bone and enamel; (**b**) Projections for natural and synthetic materials [88]. Reproduced with permission from Nature Publishing Group.

The mechanical properties and deformation of bone have been reviewed in many articles (see, for example, [74,75,78,81,88,124,125,126,127,128]). Bone is a composite material in which CaP is responsible for the mechanical durability, hardness, rigidity, and high resistance to compression, while collagen provides elasticity and resistance to tension. In the longitudinal direction, for example, cortical bone exhibits Young’s modulus of elasticity *E* = 7–30 GPa, tensile strength of 50–150 MPa, compressive strength of 167–193 MPa, 1%–3% strain to failure, and fracture toughness *K*_IC_ = 2–12 MPa·m^1/2^. In comparison, cancellous bone is characterized by *E* of only 50–500 MPa, tensile strength of 1.2–20 MPa, compressive strength of 1.9–10 MPa, 5%–7% strain to failure, and *K*_IC_ = 0.1 MPa·m^1/2^ [121,127,128]. Figure 2a shows an Ashby plot [88,124,125] of the specific values (i.e., normalized by density) of strength and stiffness (or Young’s modulus of elasticity) for both natural and synthetic materials. Many natural composite materials, as exemplified by bone, have toughness values that far exceed those of their constituents and their homogeneous mixtures (as indicated by the dashed lines in Figure 2b), and are able to sustain incipient cracking by utilizing extensive extrinsic toughening mechanisms. This results in much higher toughness for crack growth (closed circles above the solid arrows) than for crack initiation (open circles), and thus higher fracture toughness (solid arrows). The origins of fracture resistance in healthy human cortical bone can be conveniently separated into intrinsic mechanisms that promote ductility, and extrinsic mechanisms that act to “shield” a growing crack. The small-scale intrinsic and larger-scale extrinsic processes are coupled [88].

## 3. Transient Precursor Phases

Transient amorphous mineral phases have been detected in biomineral systems in different phyla of the animal kingdom [129,130]. A poorly ordered hydrated iron oxide phase, called ferrihydrite, was identified as a transient precursor mineral phase in the biomineralization forming the tooth of the chiton. After a few days, the ferrihydrite transforms into crystalline magnetite [131]. The formation of the skeleton of sea urchin made of calcite has been reported to begin with the initial deposition of amorphous calcium carbonate [132]. The presence of an abundant ACP phase has also been demonstrated in the newly formed zebrafish fin bony rays [133,134]. While the structure of bone is reasonably well defined, the process of the bone mineral formation remains controversial [80]. In solutions, ACP is readily converted to stable crystalline phases such as OCP or apatitic products. One biomineralization strategy that has received significant attention is mineralization via transient precursor phases [135].

Discussion about the possible precursor of apatite in bones arose in the 1970s, after the discovery of a precursor phase *in vitro* [136]. In the 1960s and the 1970s it was already proposed that ACP serves as a precursor to HAp *in vivo* [41,42,137,138,139,140,141]. It was argued that the initial ACP precursor undergo a solid-state transformation to poorly crystalline apatite [97]. Alternatively, it was suggested that the amorphous material observed in early mineralization is a paracrystalline mineral, i.e., it shows loss of long-range order as a result of lattice imperfections [142]. In 1972 it was shown that the first-formed phase *in vitro* is ACP [143]. This subsequently transforms into OCP, and finally into carbonate apatite. Glimcher reviewed the state of affairs in 1984 and concluded that whereas there was no evidence that ACP is a mature phase in bone, the possibility that it is a precursor phase in bone formation could not be excluded [136]. The transient ACP phase may conceivably be deposited directly inside the gap regions of collagen fibrils, but it may also be delivered as extrafibrillar particles [135]. This is consistent with the observation of collagen mineralization via a transient ACP precursor phase *in vitro* to produce aligned intrafibrillar carbonated apatite crystals [144].

The precipitation of a precursor solid phase from a CaP solution has been found to depend on the degree of its supersaturation [145]. An ACP precursor, approximating Ca_9_(PO_4_)_6_ in composition, forms under conditions of high supersaturation [42,146]. Unless stabilized in some way, this ACP would transform to thermodynamically more stable CaP phases. On the other hand, the first solid to form in low supersaturated solutions is the stoichiometric HAp, without any precursor phase. The pH value also affects the initial solid phase in the precipitation of calcium and phosphate ions. While OCP commonly forms at pH < 9.25, HAp preferentially forms at higher values of pH [147]. ACP is often the first-formed deposit *in vitro* at neutral pH and moderate supersaturation [148].

How does ACP transform to HAp at physiological pH? This has been described as a three-step process: (1) ACP dissolution; (2) a transient OCP solid phase reprecipitation through nucleation and growth; and (3) hydrolysis of the transient OCP phase into the thermodynamically more stable HAp by a topotactic reaction that is accompanied by calcium consumption from the surrounding solution and release of phosphate ions into the solution [148,149]. Based on the analysis of the measured precipitate induction time and the structure of the developing solid phase, it was proposed that OCP may be an intermediate in the conversion of ACP to apatitic calcium phosphate [150]. While simulating physiological conditions, Tung and Brown [151] used a titration method to study the conversion of high-concentration ACP slurry to HAp. The conversion kinetics indicated two processes: (1) conversion of ACP to an OCP-like intermediate, consuming acid; and (2) conversion of the OCP-like intermediate to HAp or, possibly, direct conversion of ACP to HAp, while consuming base. It was proposed that a stoichiometric HAp could be formed when there is no OCP-like intermediate phase, and a nonstoichiometric apatite product could be formed when an OCP-like intermediate phase is involved.

Watson and Robinson [152] were the first to observe the transient nature of ACP when kept in contact with its preparative medium. They found that electron diffraction patterns of ACP taken later in the precipitation reaction were no longer diffuse but resembled patterns of a poorly crystalline CDHA. Further investigations revealed that this amorphous-to-crystalline transition was not gradual but occurred rather precipitously. Initially, there is a period of a relative stability, where surfaces of the high-contrast spherules generally remain smooth and regular [153]. However, some changes occur with solid ACP during this time [154]. Afterwards, the transition follows a sigmoid evolution with the solid phase rapidly progressing from being barely crystalline to where the amorphous features disappear. Once the first crystals appear on the surface of the spherules, the transition proceeds rapidly to completion. Simultaneously, dramatic declines in ionic concentrations of calcium and orthophosphate ions occur in the mother solution [155]. The time it takes to reach this amorphous-to-crystalline boundary varies considerably with the preparation conditions, being particularly sensitive to temperature and pH [156,157]. Other publications also referred to the role of ACP in the formation of HAp [158,159,160]. A variety of proteins and ions have been proposed to be involved in the transformation of ACP to HAp [157,161,162,163,164,165,166,167,168,169,170].

The similarity between the structures of OCP and HAp has been proposed as providing geometrically favourable conditions for phase transformation from OCP to HAp [80]. OCP was argued to be a sensible precursor to HAp since they both share similar crystallographic planes, and OCP is less thermodynamically stable then apatite in physiological conditions [80]. It was suggested that the transformation is through epitaxial growth of HAp on the OCP surface, with an orientation of OCP (100)//HAp (100) and OCP (001)//HAp (001) [171,172,173]. However, Xin et al. [171] argued that there was no experimental evidence to support this proposed orientation. Tohda et al. [174] reported the presence of “modified OCP” as the initial enamel of porcine tooth germs. Crane et al. [175] used micro-Raman spectroscopy to discover evidence of OCP and other mineral species deposited during intra-membraneous mineralization.

OCP (but also DCPD) has been suggested as precursor for apatite formation by other researchers [176,177,178,179,180,181]. However, one may argue that the *in vivo* environment is complex and contains ions that might hinder the formation of OCP. For example, the presence of fluoride ions has been reported to favour the direct formation of HAp from solution, as OCP is hydrolysed when fluoride ions are present at concentrations as low as 0.05 ppm [182]. It should also be noted that recent developments in the field of glass science have improved our understanding of intermediate range order in amorphous materials, and suggest the interpretation of data in early works may need to be revised [82].

Eliaz et al. [183,184,185] reported several findings that may support the presence of a precursor phase (most likely OCP, although ACP was not excluded) in the process of electrocrystallization of HAp. Real-time, in situ electrochemical quartz crystal microbalance (EQCM) measurements revealed two phenomena during the early 11 min of deposition: (1) incubation time required for the local pH to increase; and (2) formation of a precursor phase with lower mass density and higher charge density. Analysis of the integrated intensities of the oxygen shake-up satellite peaks in the X-ray photoelectron spectroscopy (XPS), in combination with the determination of Ca/P and O/Ca atomic ratios, enabled to determine unambiguously the presence of OCP [185]. It had been reported that the integrated intensity (i.e., peak area) of the shake-up peaks is closely related to different functional groups such as O–H and P=O [186,187]. Shake-up peaks in XPS spectra, appearing at a higher binding energy than the main peak, are associated with excitation of a photoionized ion by the outgoing photoelectron, thus leaving the ion in a specific excited energy state a few eV above the ground state. Consequently, the kinetic energy of the emitted photoelectron is reduced. Eliaz et al. explained the formation of the OCP precursor phase by referring to the “Ostwald’s rule” [188], according to which the phase that nucleates first during phase transformation is not necessarily the thermodynamically most stable one, but that with free energy which is closest to the original state. Interestingly, after implanting rods electrodeposited with such CaP coatings in canine trabecular bone of dogs, Eliaz et al. [189] also found that during early stage mineralization (≤7 days), the Ca/P ratio in the mineralized tissue adjacent to the electrodeposited HAp coating resembled that in OCP, although DCPD or ACP could not be excluded.

The supersaturation of different CaP phases in solution is dependent on many parameters, such as the calcium and phosphate ion concentrations in solution, pH and temperature. In order to determine which phases are most likely to precipitate in a specific system, one should first examine which phases are thermodynamically possible. To this aim, dedicated software such as ChemEQL, AQUASIM, and PHREEQC is available and has been applied in studies of both CaP precipitation from solution and electrochemical deposition of CaP coatings [184,190,191,192,193,194,195,196,197,198,199,200]. PHREEQC not only can supply the answer to which phase is more supersaturated in solution, but also provides information on the ionic strength *I* and free ion activities product (IAP) with respect to different CaP phases. The saturation index (*SI*) indicates whether the mineral should dissolve, precipitate, or maintain equilibrium in the specific system. When *SI* = 0, the solution is in equilibrium (mineral reacts fast enough to maintain equilibrium). On the other hand, *SI* < 0 and *SI* > 0 indicate undersaturation (mineral should dissolve) and supersaturation (mineral should precipitate spontaneously), respectively. At low positive values of *SI*, a metastable zone may also exist. In this zone, although the solution is already supersaturated, the kinetics of precipitation is very slow [193]. The key equations in these thermodynamic calculations [184]:

(1) $$\log_{10}\gamma_{\pm }=-Az_{i}^{2}\sqrt{I}$$

where $\gamma_{\pm }$ is the mean ionic activity coefficient, *A* is a temperature-dependent constant, *z_i_* is the ionic charge of the aqueous species *i*, and *I* is the ionic strength of the solution, defined as

(2) $$I=\frac{1}{2}\overset{n}{\underset{i=1}{\sum }}c_{i}z_{i}^{2}$$

where *c_i_* is the analytical concentration of species *i*. The *SI* of a solution with respect to a precipitate phase is defined as

(3) $$SI=\log_{10}\left(\frac{IAP}{K_{sP}}\right)$$

where *K_sp_* is the thermodynamic solubility product. The definitions of the *IAP* and the temperature dependence of the solubility products of HAp, DCPD and OCP are given in [184].

**Figure 3:**
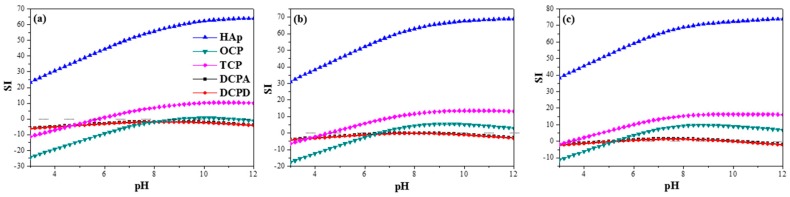
Thermodynamic calculations, using the PHREEQC software, that predict which of five different CaP phases may precipitate spontaneously in electrolyte solutions at different ion concentrations and pH values. In all cases the bath temperature is 37 °C: (**a**) X0.1 bath; (**b**) X10 bath; and (**c**) Nominal bath [191]. Reproduced with permission from Elsevier B.V.

Figure 3 [191] demonstrates the power of such calculations in the case of electrodeposition from electrolyte solution consisted of Ca(NO_3_)_2_ and (NH_4_)H_2_PO_4_ at 37 °C. The “Nominal” bath composition is 610 μM Ca(NO_3_)_2_ + 360 μM (NH_4_)H_2_PO_4_. The “X0.1” and “X10” compositions are simply a product by 0.1 or 10, respectively, of the above analytical concentrations. According to Figure 3, for all three baths the solution is most supersaturated with respect to HAp throughout the whole pH range. The extent of supersaturation increases as the pH is raised. HAp is thus expected to precipitate spontaneously from solution over the whole pH range in all three cases. TCP may also precipitate from solution within most of the pH range. As the bath becomes more concentrated, its possible precipitation extends to lower pH values (from 5.8 in Figure 3a to 3.6 in Figure 3c). At sufficiently high pH values, OCP may also form. The minimum pH value decreases from 9.2 in Figure 3a to 5.4 in Figure 3c. Although the initial pH in that study was 7.4, it is expected to rise in vicinity of the cathode during deposition, hence OCP may precipitate even from solution X0.1. Moreover, the samples were soaked in NaOH before electrodeposition. This pre-treatment has been found to increase the pH in vicinity of the cathode during electrodeposition [92]. Based on Figure 1, DCPA and DCPD cannot form in solutions X0.1 and Nominal, no matter the pH is, and will have a very small driving force for precipitation in solution X10 within the pH range 5.2–10. To conclude, the thermodynamic calculations predict the formation of HAp and OCP for the specific electrolyte solution composition, pH = 7.4, and *T* = 37 °C. Indeed, X-ray diffraction (XRD) and XPS analyses validated this prediction experimentally [191].

## 4. Dissolution and Reprecipitation as Bone

The core mechanism of CaP bioactivity is the partial dissolution and release of ionic products *in vivo* [49,87,201]. However, the dissolution rate of CaPs is mainly related to their chemical composition. Different values of *K_sp_* have been reported. In general, phases such as HAp, TCP and OCP do not dissolve easily *in vivo*. According to Table 3, OCP and TCP dissolve faster than HAp in body fluids. Yes, it should be noted that implants made of pure HAp often remain in the body for several years after implantation, thus its dissolution is still expected to be significant enough [49]. An example of the effect of different phases was given by Habibovic et al. [202], who compared the osseointegration and osteoconductive potential of porous Ti–6Al–4V, with and without OCP coating, with macro- and micro-porous biphasic calcium phosphate (BCP) ceramic in femoral defect of goat. It was found that both OCP-coated titanium implant and BCP ceramic performed better than the titanium alloy implant, yet, BCP ceramic showed higher bone amount 6 weeks after implantation, while after 12 weeks this difference was no longer significant.

Wang et al. [189] compared the osseointegration of uncoated Ti–6Al–4V implants, implants coated electrochemically with HAp (EDHA), and implants coated with HAp by the plasma spraying process (PSHA). PSHA had higher bone apposition ratio (BAR) than EDHA and uncoated titanium after 7 days; however, at 14 days after implantation, EDHA and PSHA exhibited similar BAR, much higher than that of uncoated Ti–6Al–4V. XRD tests showed that PSHA was less crystalline than EDHA. Therefore, the former is expected to be more soluble, both *in vitro* and *in vivo*. Direct current plasma atomic emission spectrometry (DCP-AES) solubility tests confirmed that the former was indeed more soluble *in vitro*. While PSHA reached saturation in distilled water in 2 days, the EDHA occasioned a very low Ca concentration even after 10 days. It was suggested that during the first 7 days, the EDHA coating made almost no contribution to bone apposition via ion release and reprecipitation, or by Ca signalling to osteoblasts [203,204]; thus it exhibited almost the same BAR as uncoated Ti–6Al–4V. In contrast, PSHA, with its partial amorphous content and consequently higher solubility *in vivo*, contributed a much higher local concentration of calcium and phosphorus ions, which could assist in and accelerate local mineralization of new bone or be involved in cell signalling. Nevertheless, the differing solubilities dictated only different short-term mineralization behaviours. By 14 days, the BAR of EDHA increased sharply and caught up with that of PSHA, suggesting that the lower dissolution rate of EDHA was already sufficient to catalyse the formation of new bone. A similar initial disparity and later catch-up was reported for annealed versus non-annealed PSHA coatings [205].

The process of dissolution/reprecipitation has been studied extensively by TEM [206,207]. These studies showed that the resorbability (in terms of dissolution *in vivo*) of BCP composed of β-TCP and HAp is dependent on the β-TCP/HAp ratio. It was further shown that the microcrystals formed had crystallographic properties and Ca/P ratio similar to that of bone apatite [207]. Moreover, the contact between the implanted biomineral and the bone did not involve a fibrous layer, yet, a linear dislocation existed at the junction of the new apatite crystals and the synthetic ceramic crystals [206].

It is believed that the dissolution process is directly linked to the bioactivity and new bone apposition on CaP ceramics. The partial dissolution causes an increase in the local concentration of calcium and phosphate ions, thereby increasing the degree of saturation in their microenviroment, resulting in the precipitation on the surface as biological apatite microcrystals that favour bone tissue apposition [49,207]. These microcrystals incorporate other ions (e.g., carbonate, magnesium) and organic macromolecules from biological fluids [207]. This surface precipitation may incorporate various proteins and growth factors (GFs) present in the microenviroment, which subsequently may promote cell attachment and function [49]. The new bone growth on the bioceramic surface forms a bridge between the host bone and the bioceramic. Eventually, this immature bone is remodelled [208].

The dissolution rate of the bioceramic affects the early stages of implantation and depends on several parameters, including cationic and anionic substitutions in CaP, the porosity in the CaP, and its particle size. Increased porosity generally enhances the surface area in contact with fluids, and thus leads to faster dissolution rates [49]. Moreover, CaP bioceramics are considered bioactive materials because they partially dissolve *in vivo*, either by cellular or extracellular activity, or both [207]. Cellular resorption usually occurs by macrophages and osteoclasts; this active cellular process is equivalent to bone remodelling [72]. Another parameter in which CaP dissolution is dependent on is the residual stress pattern on the implant [209]. Moreover, dissolution is also dependent on local acidity, fluid convection, and temperature [70]. It is important to note that adjusting the degradation rate to match the kinetics of bone formation is a big challenge in the CaP industry today [49].

Other than biomineralization in bone, CaP has been shown to induce ectopic bone formation after implantation in muscle of large animals [202,208,210,211,212,213,214]. For example, Klein et al. [208] demonstrated bone growth in porous HAp or BCP implanted intramuscularly in dogs. They showed that bone has grown in the pours of the implant, in the absence of GFs and bone marrow cells. In contrast, similar implants implanted subcutaneously in rats did not show bone formation. Although the effect of animal model and material preparation could not be excluded, it was suggested that physical and chemical factors may be responsible for the heterotopic bone formation [208]. A following research by the same group demonstrated that specimens implanted both subcutaneously and intramuscularly formed bone after 90 days in dogs and pigs, yet not in goats, rabbits, or rats after 120 days of observation [211]. Earlier periods of observation in specimens harvested from dogs showed that bone differentiation in the pore regions of the ceramics followed a complex process involving invasion of the fibrovascular connective tissues at day 15, appearance of polymorphic mesenchymal cells near the invading vasculature and at the interface with the ceramics at day 30, differentiation of osteoblasts and formation of bone matrix in direct contact with the surface of the ceramics at day 45, and finally remodelling of the fibrous connective tissue into an extensive amount of bone at days 60, 90 and 120. A following research demonstrated the importance of microporosity—no bone growth on CaP ceramic was observed after 180 d in its absence [212]. Regardless of dissolution-reprecipitation models, Wang et al. [215] recently emphasized the role that water itself has in structuring of bone apatite. By using solid-state nuclear magnetic resonance (NMR), wide-angle X-ray scattering and cryogenic TEM to characterize the structure and organization of crystalline and biomimetic apatite nanoparticles as well as intact bone samples, they showed that water orients apatite crystals through an ACP-like layer that coats the crystalline core of bone apatite.

Several models have been proposed to describe the dissolution process of CaP in acidic environment [209,216]. They take into account the electrolyte solution conditions (pH, composition, level of supersaturation, and hydrodynamics), bulk solid (chemical composition, solubility, and particle size), and surface of the apatite crystals (defects, adsorbed ions, “history”, and phase transformation). Ducheyne and Qiu [217] described a set of 11 successive reaction steps that take place at the interface between bioceramics and the surrounding biological environment: (1) dissolution of the bioceramic; (2) precipitation from solution onto the bioceramic; (3) ion exchange and structural rearrangement at the bioceramic/tissue interface; (4) interdiffusion from the surface boundary layer into the bioceramic; (5) solution-mediated effects on cellular activity; (6) deposition of either the mineral phase or the organic phase without integration into the bioceramic surface; (7) deposition with integration into the bioceramic; (8) chemotaxis to the bioceramic surface; (9) cell attachment and proliferation; (10) cell differentiation; (11) extracellular matrix (ECM) formation. All phenomena, collectively, lead to the gradual incorporation of a bioceramic implant into developing bone tissue. It should be noted that there are other descriptions of the events which take place at the bone/implant interface, that are focused on biochemical considerations [218,219]. The osteoconduction of bioactive, bioresorbable CaP coatings has been described [220,221] as follows: (1) The decrease in local pH leads to partial dissolution of the coating and subsequent calcium and phosphate ions release into the microenvironment; (2) The ions reprecipitate and incorporate into apatite crystals and form with collagen matrix; (3) The increased concentrations of calcium and phosphate ions stimulate chemotaxis. This supports the natural healing process. The bioactive CaP coating is only necessary until osseointegration progresses into the underlying metal (say, titanium) substrate. Once this occurs, the mineral component is absorbed.

## 5. Requirements from Calcium Phosphates for Medical Applications

Calcium phosphates are commonly used in medical applications in the form of cements, coatings, scaffolds, and paste. To function properly, a variety of properties may be required [32,209,222,223,224,225], some of which are listed in Table 2.

The core mechanism of bioactivity is the partial dissolution and release of ionic products *in vivo*, elevating the local concentrations of calcium and phosphate ions and precipitating a biological apatite on the surface of the ceramics [72]. All implantable materials must be biocompatible, meaning that they do not elicit local or systemic response of the living system or tissue. All CaP ceramics have been found to be biocompatible [72]. This is because of their abundance in the body in either dissolved or solid form [77]. For example, it was also found that HAp implantation showed no inflammation or foreign body response [208].

A critical problem that limits wider clinical application of CaPs is their mechanical properties [226]. The hip joints are subjected to an average load of up to three times body weight (3000 N); peak loads experienced during jumping can be as high as 10 times body weight. These stresses are repetitive and fluctuating depending on the nature of the activities, which can include standing, sitting, jogging, stretching and climbing. Therefore, all types of potential biomaterials and bioceramics must be durable within a wide variety of conditions [227]. Unfortunately, CaPs are brittle and have low impact resistance and relatively low tensile stress (6 to 10 MPa) [226]. The main reason for this is their porosity, which serves as preferred initiation sites for crack propagation. Yet, their compressive strength is fairly good, being higher than that of normal bone [226]. Therefore, CaP is used either as non-load bearing implants such as middle ear surgery, filling of bone defects in the oral cavity and skeleton, or as coating on dental and orthopedic metallic implants. The brittle nature of CaPs is related to their primary ionic bonds.

Osteoconduction and osseointegration involve support of cell adhesion/proliferation and integration of cells in the CaP [226,228]. Cell adhesion is influenced mainly by the CaP ability to adsorb ECM proteins (e.g., fibronectin). In the case of CaPs, this ability is strongly influenced by their surface roughness, percent of crystallinity, solubility, phase content, grain size, particle size, surface charge, and surface energy [228]. Osteoinduction is the ability of a material to recruit and induce progenitor cells to differentiate towards the osteoblastic linage [226,228]. Several studies suggested that CaP, in the absence of supplements, are osteoinductive [228]. However, osteoinduction depends on several properties of the CaP. For example, its surface chemistry and charge can influence protein adsorption to it, and in turn drive osteoblastic differentiation via cell-ECM interaction. Likewise, physical properties such as surface morphology can influence in the same manner [228].

Resorption is the process by which the bioceramic is absorbed in the body, either by cells (such as macrophages and osteoclasts) or by dissolution [72]. This ability is dependent on the phase content of the CaP, particle size, crystallinity and porosity [72,226]. Some phases may resorb fast and replace the coating or cement with bone, as will be discussed later. Increasing porosity greatly enhances the surface area in contact with body fluids, thus leading to faster dissolution rate [72]. Lattice defects are particularly involved in the process of dissolution, which can explain the large differences in solubility of different HAp scaffolds [72]. This trait is an important property, as cements and coatings could provide short-term biologically desired properties and then be replaced by new bone. The rate of bone substitution also depends on age, sex and general metabolic health of the recipient, as well as on the anatomic site [226]. Considering these factors, it may take 3 to 36 months for CaP to be replaced by bone [226]. The desired resorbability rate is the rate comparable to the formation of bone tissue (i.e., between a few months and a few years) [77].

Porosity of CaP is not important only for its mechanical and resorbability, but also for ingrowth of bone. In porous form, CaP can permit the ingrowth of bone tissue and cells. Therefore, CaP was traditionally macroporous, with pore diameter of ~100 μm [77]. Studies have shown that increasing the specific surface area and pore volume of biomaterials for tissue repair may greatly accelerate the kinetic process of biological apatite deposition and therefore enhance the bone formation [229].

The wettability (or hydrophilicity) of CaPs is extremely important since surface energy is an important factor in osteogenesis regulation. Generally, when the implant’s surface is positively charged, the surface becomes hydrophilic, and some plasma proteins essential for cell interaction adsorb to the surface [230]. High surface energy (hydrophilic) implants have been found to be associated with an enhanced fibroblast cell response. Aronov et al. [231] tuned the wettability of HAp (10° < θ < 100°, where θ is the contact angle) by an innovative post-treatment of exposure to low-energy electron irradiation and found that DNA tended to bind to surfaces with θ < 50°. The surface energy has also been shown to affect the bone cell maturation and differentiation [232] and the osseointegration [233]. It was also shown [234] that the cellular reaction is different for hydrophilic and hydrophobic implants, especially in the initial stages of wound healing. Surfaces with a higher surface energy exhibited more rapid cell activation and differentiation than those with lower surface energy. The adhesion and proliferation of osteoblasts have been correlated with substratum wettability, the cells exhibiting a strong preference for hydrophilic substrata [235,236]. Eliaz et al. [237] found that osteoblast progenitors derived from rats may be attached preferentially to a hydrophilic surface. In another study, Eliaz et al. [238] found that the very high hydrophilicity of their as-deposited HAp coating enhanced its bioactivity, as reflected by *in vitro* cell study (mouse marrow osteogenic cell line MBA-15, which expresses osteoblastic phenotype *in vitro* and forms bone *in vivo* was used).

A key factor for the successful fixation of cementless implants used for joint reconstruction is the establishment of a stable interface between the implant and bone [55]. There are four types of bioceramic-tissue attachment: (1) Morphological fixation, where dense, nonporous, nearly inert ceramics attach by bone growth into surface irregularities by cementing the device into the tissue or by press-fitting into a defect; (2) Biological fixation, where mechanical attachment occurs due to porous surface; (3) Bioactive fixation, where reactive surfaces (e.g., of HAp) form chemical bonding, thus minimizing the fibrous capsule formation; and (4) Dense, porous (or nonporous) resorbable bioceramics (e.g., TCP) are designed to be slowly replaced by bone.

Polymethyl methacrylate (PMMA) bone cement is widely used for hip implant fixation into the medullary canal of the femur (e.g., the Thompson prosthesis). However, it suffers from some major adverse effects. The elastic modulus of PMMA is 2700 MPa, much higher than that of human cancellous bone (50–500 MPa). Consequently, the bone is exposed to stress shielding, and the adjacent tissue might fracture. Thermal and chemical necrosis of the surrounding tissues might also occur. When the cement hardens, it might heat to as high as 96 °C; it has been claimed that this might cause cardiac arrest by neurogenic stimulation. The heat generated by the cement increases the pressure of air trapped in the femoral shaft and forces it through damaged sinusoids into extra-osseous veins. The absorption of the acrylic MMA monomer in the systemic blood system might cause pulmonary embolism, hypotension, and cardiac arrest. Consequently, the risk of death during operation when using PMMA is ~1%. Furthermore, cemented implants suffer from higher rates of loosening, bone loosening and infection, require higher surgeon expertise, and are more difficult for revision.

Morphological fixation by press-fit (e.g., the uncemented Austin-Moore hemiarthroplasty) suffers from its own drawbacks, including relatively poor outcomes in active patients and a marked potential for acetabular erosion. Moreover, such prostheses are closely dependent on the structure of the medullary canal, which is not uniform in different patient populations. If the medullary canal is too small, iatrogenic fracture might occur. On the other hand, if the canal is too wide, the fixation might be insufficient. Improper placement of the prosthesis and the resulting biomechanical disturbances within the hip joint, inadequate calcar seating, insufficient residual femoral neck length, insufficient metaphyseal fill, and errors in sizing the prosthesis are all associated with failure of this prosthesis. Press-fit prostheses often cause post-surgery pain due to local loading at the contacting points between the femoral stem component and the cortex. Insertion of uncemented prostheses involves the use of a reamer, which prepares the canal according to the size of the femoral component. Consequently, the adjacent femur is weakened, and the rate of iatrogenic fractures is increased. Finally, when revision is necessary, it is difficult to detach this prosthesis, to the extent that the femur might fracture as a result of bone ingrowth into the prosthesis.

In contrast to PMMA fixation, the elastic modulus of CaP cements (CPCs) is 180 MPa, similar to that of human cancellous bone; thus, CPCs are more effective in avoiding the stress shielding effect, as well as reducing secondary fracture of adjacent tissue. The CPC is highly osteoconductive, and is gradually replaced by new bone that can provide substantial improvement in the compressive strength of osteoporotic or fractured bone. However, CPC is prone to failure under shear loading, might not provide enough initial stiffness, and therefore progressive and repeated collapse might happen.

The use of CaPs in the form of cements poses some specific requirements. For example, the cement must set slowly enough to provide the surgeon sufficient time to implant, but fast enough to prevent delay in operation (deformation during setting time causes cracks) [239,240,241]. The setting time of many CaP cements in their virgin composition is between 15 and 22 min. This setting time is too long for some clinical applications, thus natural phosphates are sometimes added to reduce the setting time to 5–8 min [226]. Another requirement is for proper viscosity. In the clinics there are two kinds of cements—those applied by injection in the form of paste, and those applied and moulded by the surgeon; each requires a different degree of viscosity [239]. Currently, injection appears to be the preferred method between these two major options. Viscosity values in the range of 100–2000 Pa·s are generally considered adequate [239].

## 6. Individual Calcium Phosphate Phases and Their Properties

In this Section, the properties of CaP phases relevant to clinical use and biomedical applications are summarized. Here, the focus is on each, individual phase. The properties of composites and FGMs are summarized in Section 9 and Section 10, respectively.

**Figure 4:**
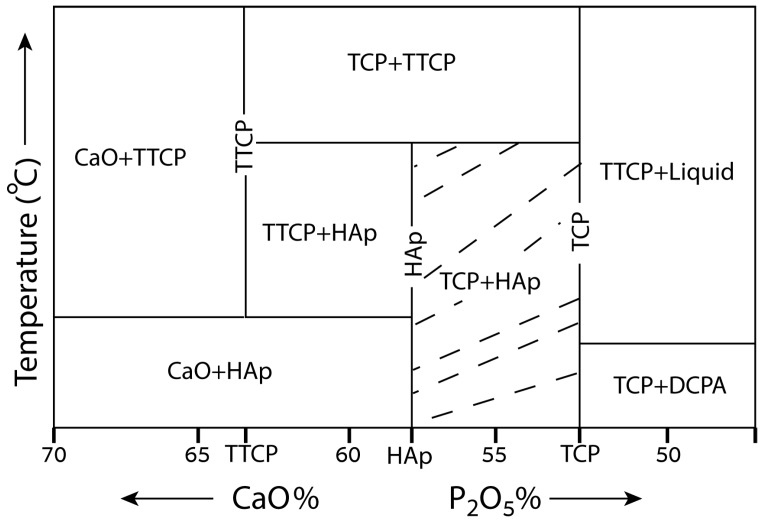
Equilibrium phase diagram of different calcium phosphates. The shaded region shows the phases of interest for biphasic calcium phosphate (BCP) formation (*T*_1_ = 1360 °C, *T*_2_ = 1475 °C) [245]. In this figure, TTCP—tetracalcium phosphate, CaO—calcium oxide. Reproduced with permission of Elsevier Ltd.

The solid-state phase diagram of the CaP system is relatively unexplored. One of the reasons could be the important role that metastable phases, water, and kinetics have in this system. Water, for example, is essential in defining the thermodynamically stable salts [242]. Nevertheless, few versions of this phase diagram are available [243,244,245]. Figure 4 shows one version [245]. The shaded region shows the area of BCP formation. BCP can be prepared by mechanical mixing of HAp and β-TCP, or by a precipitation method [246]. Solid-state reaction [247], microwave processing [245], and heating bovine cancellous bone with the addition of (NH_4_)_2_HPO_4_ [248] were also reported to prepare BCP.

Table 3 lists the major members of the CaP family that are of interest to biomedical applications, according to their Ca/P atomic ratio, their pH stability range in aqueous solutions at 25 °C, and their density. Table 4 lists their solubility, while Table 5 presents their crystallographic structure. It is evident from Table 4 that the lower the Ca/P atomic ratio is, the more acidic and water-soluble the CaP phase is [70]. The following subsections will review the main characteristics of each phase.

### 6.1. MCPM

MCPM is the most acidic and water-soluble CaP phase. It does not form in living organisms. Yet, it is used as a component of several self-hardening CaP cements [77,249,250,251] and as sealer in dentistry [252]. It is associated with the first acidic proton of H_3_PO_4_, and can be prepared by partial neutralization of the phosphoric acid with calcium hydroxide, followed by evaporation of water at low temperature, in acidic conditions [253]. The hydrated monocalcium phosphate crystallizes as platelets elongated along the *c*-axis of the triclinic structure [249]. MCPM-containing chewing gum was found to produce significantly greater saliva flow and lower salivary pH than a control gum [77,254]. MCPM is marked as food additive E341 and is often added to toothpastes. However, pure MCPM is not biocompatible with bone due to its acidity [77,255].

### 6.2. DCPA

The second acidity of phosphoric acid corresponds to a weak acid. The neutralization of two acidities of phosphoric acid with calcium hydroxide leads to dicalcium phosphates. Two crystalline forms exist: DCPD (also called Brushite by mineralogists) and DCPA (also called Monetite) [249]. DCPA is the anhydrous form of DCPD. It is less soluble than DCPD due to the absence of water inclusions. DCPA, like DCPD, can be crystallized from aqueous solutions, but at 100 °C. Unlike DCPD, DCPA occurs in neither normal nor pathological calcifications. It is used in calcium phosphate cements, sources of calcium and phosphate in nutritional supplements such as breakfast cereals, and toothpaste components [77,249,252,255].

### 6.3. DCPD

DCPD is the most easily synthesized CaP compound [77,249,252,255,256]. It is biocompatible, biodegradable, and osteoconductive, and can be converted into DCPA (pH < 6), OCP (pH ≈ 6–7), or pHAp (pH > 7). It is observed that DCPD can convert *in vivo* into either pHAp [257], or it will be degraded and replaced by bone. Brushite may form as an intermediary phase in pathological calcification occurring in slightly acidic media (for example, in dental calculi, urinary calculi, and urinary stones) [179,249,255]. DCPD has been proposed as an intermediate in both bone mineralization and dissolution of enamel in acids (dental erosion) [64,258,259]. In medicine, DCPD is used in CaP cements [260,261,262,263,264], and as an intermediate for tooth remineralization [255]. DCPD is added to toothpaste both for caries protection (in this case, it is coupled with F-containing compounds such as NaF and/or Na_2_PO_3_F), and as a gentle polishing agent [265,266,267,268]. When large amounts of DCPD are converted into pHAp *in vivo*, a severe inflammatory response can be observed due to the large amounts of acid that are released during this reaction [64]. DCPD crystals can be prepared simply by neutralization of phosphoric acid with calcium hydroxide at pH between 3 and 4 at room temperature. In general, DCPD can be obtained by double decomposition between calcium and phosphate containing solutions in slightly acidic media. It can also be formed by conversion of calcium phosphate salts, in acidic media, or by reaction of calcium salts such as calcium carbonate in acidic orthophosphate solutions [249]. DCPD crystals consist of CaPO_4_ chains arranged parallel to each other, while lattice water molecules are interlayered between them [255].

### 6.4. OCP

OCP is of a great biological importance because it is one of the stable components of human dental and urinary calculi [269,270,271]. It was Brown [172,272,273] who first suggested that OCP participates as the initial phase in enamel mineral formation and bone formation through subsequent precipitation and stepwise hydrolysis of OCP. Thus, OCP plays an important role in the *in vivo* formation of apatitic biominerals. A “central OCP inclusion”, also known as “central dark line”, is revealed by TEM in many biological apatites and in some synthetically precipitated HAp [274,275,276,277]. It has been explained by the inherent lattice mismatch between OCP and HAp. OCP has not been observed in vascular calcifications [255]. However, it has been suggested as a precursor phase to biological apatite found in natural and prosthetic heart valves [255,278,279]. In medicine, OCP is used for implantation into bone defects [271,280,281,282,283,284]. OCP has been used as a coating [249,285,286], a component of biocomposites [287], and self-setting formulations [288]. OCP coatings have been found to exhibit an osteoinductive behaviour [286]. OCP has been evaluated as a direct pulp capping material [288] and for alveolar ridge augmentation [287,289]. Investigations with rats revealed that implanted OCP could serve as a core for initiating bone formation and cause osteoinduction and osteoconduction in experimentally created cranial defects [252,290].

OCP has a remarkable structural similarity to HAp due to its layered structure involving apatitic and hydrated layers. The triclinic structure of OCP displays remarkable similarities to the hexagonal structure of HAp because the unit cell of OCP consists of apatitic layers, and apatitic layers alternate with hydrated layers parallel to the (100) face, while the hydrated layer contains lattice water and less densely packed calcium and phosphate ions [291]. Morphologically, OCP crystallizes as {100} blades of triclinic pinacoidal symmetry, elongated along the *a*-axis and bordered by the forms {010}, {001}, and {011} [291]. It is generally assumed that, in solutions, the hydrated layer of the (100) face is the layer most likely exposed to solution. The water content of OCP crystals is about 1/5 that of DCPD, and this is partly responsible for its lower solubility. The similarity in crystal structure between OCP and HAp is one reason that the epitaxial growth of these phases is often observed [291].

OCP is unstable relative to HAp and tends to hydrolyse according to the reaction [291]:

Ca_8_H_2_(PO_4_)_6_⋅5H_2_O + 2Ca^2+^ → Ca_10_(PO_4_)_6_(OH)_2_ + 4H^+^ (4)

The full hydrolysis of OCP into CDHA occurs within ~6 h [292]. Furthermore, OCP might be non-stoichiometric and be either Ca-deficient (Ca/P = 1.26) or include excessive calcium (up to Ca/P = 1.48) in the structure [293]. Most data concern OCP hydrolysate’s conversion into apatite. Most OCP preparations lead to a partly hydrolysed phase that contains an excess of $HPO_{4}^{2-}$ and $OH^{-}$ observable by FTIR and solid-state NMR [249].

### 6.5. α-TCP

The third acidity of phosphoric acid is very weak and, although $PO_{4}^{3-}$ exists only in a very small amount at pH lower than 11 (according to the speciation curves of phosphoric acid at 37 °C, p*K*_3_ = 12.023 [184]), TCP salts can precipitate due to their very low solubility. The term TCP is used here in its strict chemical meaning to designate phases with a chemical composition represented by Ca_3_(PO_4_)_2_ with a Ca/P ratio close to 1.5. Several different phases with a composition close to TCP exist. Crystalline TCP (α- and β-TCP) form only at high temperature; it is generally agreed that crystalline, pure, TCP cannot be obtained by direct precipitation from aqueous media [294]. α-TCP is usually prepared from β-TCP by heating at above 1125 °C and quenching to prevent the reverse transformation; thus, α-TCP may be considered a high-temperature phase of β-TCP. Although α-TCP and β-TCP have the same chemical composition, they differ in their solubility (Table 4) and crystal structure (Table 5). In the absence of humidity, both polymorphs of TCP are stable at room temperature; however, a density functional study [295] has shown that the stability of the β-TCP crystal lattice exceeds that of α-TCP. Therefore, of the two, α-TCP is more reactive in aqueous systems, has a higher specific energy, and can be hydrolysed in aqueous solutions to CDHAp [296,297]. Although α-TCP never occurs in biological calcifications, it is used as a component of CaP cements [256,260,261,262,263,264]. The major disadvantage for using pure α-TCP is its quick resorption rate—faster than the formation of a new bone—which limits its use in biomedical applications. The structure of α-TCP has been described in [298], while its surface and adsorption properties were described in [299].

### 6.6. β-TCP

β-TCP crystallizes in the rhombohedral space group *R*3*c*, and its unit cell contains twenty-one Ca_3_(PO_4_)_2_ formula units [300]. There are three types of crystallographically nonequivalent $PO_{4}^{3-}$ groups located at general points of the crystal, each type with different intra-tetrahedral bond lengths and angles [300]. Both HAp and β-TCP exhibit similar Raman spectra, which are dominated by the internal modes of the $PO_{4}^{3-}$ tetrahedral [291]. However, besides the presence of peaks associated with vibrations of the $OH^{-}$ group in the Raman spectrum of HAp, which are highly sensitive to sample crystallinity, other characteristic features, such as the width of the $PO_{4}^{3-}$ internal bands, can be used to distinguish between HAp and β-TCP [301].

β-TCP cannot be precipitated from aqueous solutions. Mg-stabilized β-TCP (Whitlockite) has been identified during pathological calcification, such as dental calculus formation and in renal stones, as well as in arthritic cartilage. However, β-TCP has not been observed in enamel, dentin, or bone [77,302]. β-TCP can be prepared at temperatures above 800 °C by thermal decomposition of CDHAp, or by solid-state interaction of acidic CaPs, e.g., DCPA, with a base, e.g., CaO. Nevertheless, β-TCP can be obtained at a relatively low temperature (150 °C) by precipitation in organic medium, such as ethylene glycol [303]. Apart from the chemical preparation routes, ion-substituted β-TCP can be prepared by calcining of bones [304]; such a type of β-TCP is occasionally called “bone ash”. In biomedical applications, β-TCP is used in CaP bone cements [46,305,306] and other types of bone substitution bioceramics [304,307,308], as well as in dentistry [309]. In combination with HAp, β-TCP forms BCP and is used as a bone-substitution bioceramic [77]. Pure β-TCP is added to some brands of toothpaste as a gentle polishing agent. β-TCP is also added as a dietary or mineral supplement to food and feed, where it is marked as E341 according to the European classification of food additives. A review of the toxicology of β-TCP and other CaPs as food ingredients is provided in [310]. β-TCP is considered to be both osteoconductive and osteoinductive, and due to its low interfacial energy with respect to apatite, it can provoke the precipitation of an apatite layer upon incubation in aqueous ionic solutions.

### 6.7. ACP

Amorphous calcium phosphates (ACPs) represent a special class of CaPs, having variable chemical composition but similar glass-like physical properties, in which there are neither translational nor orientational long-range order (LRO) of the atomic positions. Depending on the processing temperatures, ACPs are divided into two major groups: (1) low-temperature ACPs, prepared in aqueous solutions; and (2) high-temperature ACPs. Low-temperature ACPs are often encountered as a transient precursor phase during precipitation of other CaPs in aqueous systems (see Section 3). ACPs are thought to be formed at the beginning of the precipitation due to a lower surface energy than that of OCP and apatites [153]. The degree of amorphization of ACPs increases as the concentrations of $Ca^{2+}$ and $PO_{4}^{3-}$ and/or the pH of the electrolyte solution are increased. A continuous gentle agitation of as precipitated ACP in the mother solution, especially at elevated temperatures, results in a slow recrystallization and formation of more crystalline CaP, such as CDHA [64,66].

The chemical composition of ACPs strongly depends on the solution pH and the concentrations of the mixed solutions. For example, ACPs with Ca/P atomic ratios in the range of 1.18 (precipitated at pH = 6.6) to 1.53 (precipitated at pH = 11.7) [66], and even 2.5 [64,258,259] have been described. FTIR spectra of ACPs show broad featureless phosphate absorption bands [138]. Electron microscopy of freshly precipitated ACPs usually shows featureless, nearly spherical, particles with diameters in the range of 20 to 200 nm. It was proposed that the basic structural unit of precipitated ACPs is a 9.5 Å diameter, roughly spherical cluster of ions with the composition of Ca_9_(PO_4_)_6_ [41,42,66,137]. These clusters were found experimentally, first as nuclei during the crystallization of CDHA. A model was developed to describe the crystallization of HAp as a step-wise assembly of these units [311].

Biologically, ion-substituted ACPs (always containing ions of Na, Mg, carbonate and pyrophosphate) are found in soft-tissue pathological calcifications (e.g., heart valve calcifications of uremic patients) [250,260,312]. In medicine, ACPs are used in CaP cements [261,262,263], as bone substitution materials, and in other dental applications [159,313,314,315,316,317,318,319,320,321,322,323,324,325,326,327,328,329,330,331,332,333,334,335,336,337,338]. In the acidic oral environment, ACP-based biocomposites take advantage of the ability of ACPs to release calcium and phosphate ions, which may participate in enamel remineralization [315,316,317,318,321,324,339,340,341,342,343,344]. The ACP-containing biocomposites and hybrid biomaterials are used as anticariogenic and/or remineralizing agents (e.g., in chewing gums), sugar confections, tooth mousses, bleaching gels, mouth rinses, various drinks, or even in milk [252]. The ability of ACPs to release calcium, phosphate and other ions in aqueous environments is thought to contribute towards their osteoinduction [146,228]. However, this rapid release of ions from ACPs can cause perturbations in the local pH and negatively impact cell attachment/proliferation in the short term, and viability in the long term [228,345]. The inclusion of divalent cations such as Zn and ZrO can lower their dissolution rates, and the incorporation of Zn and Cu can impede their conversion to HAp [228,317,346].

### 6.8. CDHA

CDHA, sometimes referred to as pHA, can be easily prepared by simultaneous addition of calcium- and orthophosphate-containing solutions into boiling water, followed by boiling the suspension for several hours. During this time, the initially precipitated ACP is restructured and transformed into CDHA. Therefore, there are many similarities in the structure, properties and applications between ACP precipitated in alkaline solutions (pH > 8) and CDHA [77,255]. Besides, CDHA can be prepared by hydrolysis of α-TCP. CDHA crystals are poorly crystalline and of submicron dimensions [256]. On heating above 700 °C, dry CDHA with Ca/P = 1.5 will convert to β-TCP and that with 1.5 < Ca/P < 1.67 will convert into a mixture of HAp and β-TCP (i.e., to BCP) [77,255,347,348,349]. The variability in Ca/P molar ratio of CDHA has been explained by different models, such as surface adsorption, lattice substitution, and intercrystalline mixtures of HAp and OCP [350]. Due to a lack of stoichiometry, CDHA usually contains other ions [255,351]. The unit cell parameters of CDHA have not been fully determined yet [255]. Nevertheless, some useful information is available in [352,353,354,355,356,357,358]. As a first approximation, CDHA may be considered as HAp with some ions missing [359]. The more calcium is deficient, the more disorder and imperfections are in the CDHA structure [255,360].

Unsubstituted CDHA does not exist in biological systems. However, the ion-substituted CDHA (i.e., which contains $Na^{+}$, $K^{+}$, $Mg^{2+}$, $Sr^{2+}$ for $Ca^{2+}$; $CO_{3}^{2-}$ for $PO_{4}^{3-}$ or $HPO_{4}^{2-}$; $F^{-}$, $Cl^{-}$, $CO_{3}^{2-}$ for $OH^{-}$) with some water forms biological apatite [64,258,351]. Hence, CDHA is of interest for artificial bone substitutes [77,255]. All commercially available CaP cements (CPCs) have CDHA as a compound [256].

### 6.9. TTCP

TTCP is the most basic CaP. However, its solubility in water is higher than that of HAp. TTCP cannot be precipitated from aqueous solutions. It can be prepared only by a solid-state reaction above 1300 °C [255]. However, the reaction has to be carried out under dry atmosphere or vacuum in order to avoid, in the presence of water vapour, the decomposition of TTCP to HAp [249]. TTCP often appears as an unwanted byproduct in plasma-sprayed HAp coatings, where it is formed as a result of the thermal decomposition of HAp to a mixture of high-temperature phases of α-TCP, TTCP, and CaO [361]. TTCP is metastable: in both wet environments and aqueous solutions, it slowly hydrolyzes to HAp and calcium hydroxide. Consequently, TTCP is never found in biological calcifications [255]. TTCP is rarely used as a single component in dentistry [252]. However, it is used in combination with other CaPs, mainly with DCPA or DCPD, to form various self-setting cements [249,362,363,364,365,366,367,368], biocomposites [362,363,364,369], and root canal sealers [370]. Due to the alkaline pH generated by dissolution of TTCP in water, this phase transforms very easily into apatite [249].

### 6.10. HAp

Stoichiometric HAp has the chemical formula Ca_5_(PO_4_)_3_(OH). However, it is commonly written as Ca_10_(PO_4_)_6_(OH)_2_ (see Table 3) to denote that the hexagonal unit cell is comprised of two molecules [77,255]. In the International Union of Pure and Applied Chemistry (IUPAC) nomenclature, its name is pentacalcium hydroxide tris(phosphate) [70]. HAp is the second most stable and least soluble after fluorapatite (FAp) of all CaPs (see Table 4). Although not highly soluble, HAp surface provides nucleating sites for precipitation of apatite crystals in culture medium (typically, saturated with calcium and phosphate ions) and in body fluids [228]. The HAp derived either from natural sources or from synthetic sources is regarded as bioactive substance, since it forms a strong chemical bond with host bone tissue, and hence it is recognized as a good bone graft material. HAp is not only bioactive but also osteoconductive, non-toxic, non-immunogenic, and its structure is crystallographically similar to that of bone mineral with adequate amount of carbonate substitution [121].

**Figure 5:**
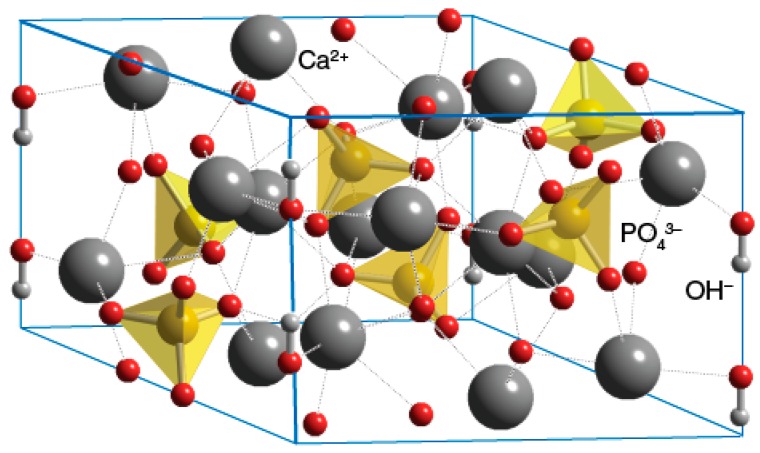
Unit cell of hexagonal HAp (space group *P*6_3_/*m*) [375]. Reproduced with permission from ChemTube3D, The University of Liverpool.

Chemically pure HAp crystallizes in the monoclinic space group *P*2_1_/*b* (see Table 5). However, at temperatures above ~250 °C, there is a monoclinic-to-hexagonal phase transition to HAp (space group *P*6_3_/*m* (176)) [70,173,371,372,373,374]. The hydroxide ions in hexagonal HAp are more disordered within each row than in the monoclinic form, pointing either upward or downward in the structure. This induces strains in the hexagonal lattice that are compensated for by substitutions or ion vacancies. These stabilize the hexagonal structure of HAp at ambient temperature [70]. Hence, hexagonal HAp is the common form in biology and medicine [70]. The structure of hexagonal HAp is illustrated in Figure 5. In this structure, the calcium ions can occupy two sites labelled as I and II. Calcium I sites are on the trigonal axis of the structure at (1/4, 3/4, 1/2 and 3/4, 1/4, 1/2 positions). The Ca II ions form equilateral triangles at *z* = 1/4 and *z* = 3/4, on the 6_3_ axis of the structure. These ions constitute part of the walls of ‘channels’ where the monovalent sites are located. They correspond to the narrowest part of the channels with a diameter of 0.27 nm for CaP apatites. At *z* = 1/2, the channels appear slightly larger (0.29 nm) and they are limited by a distorted hexagon of oxygens belonging to ${\mathrm{PO}}_{4}^{3-}$ anions. Owing to the existence of these channels, apatites have sometimes been compared to zeolithes; the channels appear, however, smaller than those generally found in zeolithes and they are mono-dimensional and obstructed by ions, which limit considerably the exchanges at low temperature and the trapping of molecules [66,249].

HAp is considered to be osteoconductive, but not osteoinductive; yet, these properties can be turned via ionic substitution [228]. For example, anionic substitution of carbonate for phosphate has been shown to increase HAp solubility (and bioactivity), while substitution of fluoride for hydroxide increases stability. Moreover, cationic substitutions such as magnesium in place of calcium can potentially have favourable biological effects by providing trace ions [228]. The structure of HAp was described in [40,173]. There are several JCPDS files for HAp (see Table 5) [376,377,378,379,380,381,382]. The difference between them is both in crystal orientation and in pairing to a crystallographic system. In regard to synthesis of HAp, it is important to note that producing pure stoichiometric HAp is a challenge, thus the lattice parameters might vary [173,374].

HAp is the common mineral found in vertebrate bones. It is also found in mammalian teeth, fish scales, and the mature teeth of some chiton species [110]. Moreover, in the early 1900s, XRD patterns found ground bone to be similar to geological HAp [110]. However, subsequent studies showed that the atomic Ca/P ratio in biomineralized tissues can vary significantly due to ion substitutions and vacancies, as described before. HAp is recognized as the final solid mineral of the bone. All other phases have been categorized as minor or precursor phases; they are acid stable and will convert to the thermodynamically stable HAp at high pH. Table 6 summarizes some of the key properties of HAp.

The strength of HAp was found to increase as the Ca/P atomic ratio was increased, reaching a maximum value at Ca/P ~1.67 (stoichiometric HAp) and decreasing suddenly when Ca/P > 1.67 [383]. Furthermore, the strength decreases almost exponentially with increased porosity. However, by changing the pore geometry, it is possible to influence the strength of porous bioceramics. It is also worth mentioning that porous HAp is considerably less fatigue resistant than dense HAp. A considerable anisotropy has been observed in the stress-strain behaviour of perfect HAp crystals [386]. The crystals appeared to be brittle for tension along the *z*-axis, with maximum stress of ~9.6 GPa at 10% strain. Furthermore, the structural analysis of the HAp crystal under various stages of tensile strain revealed that the deformation behaviour manifested itself mainly in the rotation of PO_4_ tetrahedrons, with concomitant movements of both the columnar and axial Ca ions [386]. The wear resistance, friction coefficient and hardness of dense HAp are comparable to those of dental enamel [374].

Unsintered HAp is usually poorly crystalline, and often non-stoichiometric, resembling CDHA. On the other hand, well-crystalline HAp can be prepared from aqueous solutions [387]. Pure, stoichiometric HAp never occurs in biological systems. However, due to the chemical and phase (and, sometimes, shape) similarities to the biological mineral in bone and teeth, clinical uses of HAp range from augmenting atrophic alveolar ridges to repairing long bone defects, ununited bone fractures, middle ear prostheses, spinal fusions, and craniofacial repair. It has also been used in dental surgery, biomolecular delivery, and drug delivery [121]. Synthetic HAp is widely used as a coating on orthopedic (e.g., hip joint) and dental implants [383,388,389,390,391,392,393,394]. HAp particles have also been inserted into postextraction alveolar sockets to maintain the alveolar ridge height [395]. HAp scaffolds and nanoparticles have been used also for controlled drug delivery [396,397,398,399]. Also, HA is added to some brands of toothpaste as a gentle polishing agent instead of calcium carbonate [400,401]. Nano-HAp particles were found to have an ability to infiltrate a demineralized collagen matrix of dentin. Afterwards, the infiltrated collagen matrix of dentin can provide a suitable scaffold for dentin remineralization, whereby the infiltrated HAp particles could act as seeds within the collagen matrix and, given the appropriate remineralizing environment, dentin remineralization may occur [402]. In addition, it was demonstrated that HAp nanorods could be self-assembled to form enamel-like structures [403]. Review of these and other applications is provided elsewhere [65,404,405,406,407].

## 7. Nano-CaP

Usually, when we refer to the nanoscale, we have in mind dimensional range from 0.1 nm to 100 nm. In comparison, a particle 100 nm to 1 µm in size will be referred to as submicron particle [408]. As described in Section 2, the HAp in bone is nanocrystalline. Moreover, nanoparticles (NPs) smaller than 100 nm have the highest reported efficacy with respect to cellular integration; it has been suggested that these NPs induce responses different from submicron structures, which could imply that nano-HAp, and possibly other nano-CaPs, could form biocompatible surfaces that integrate well with bone tissue [409,410,411].

Nanoscale HAp has superior functional properties over its microscale counterpart, particularly surface reactivity and ultrafine structure, which are the most imperative properties for tissue-graft interaction upon implantation. Its high surface area-to-volume ratio, superior chemical homogeneity, and microstructural uniformity result in enhanced bone integration and mechanical properties [412]. It has been shown that nano-HAp promotes enhanced osteoblast adhesion, differentiation and proliferation, osteointegration, and deposition of Ca-containing minerals on its surface, compared to conventional micro-HAp, which leads to enhanced formation of new bone tissue within a short period [413]. The conventional removal torque (RTQ) evaluation and gene expression in tissues around nanostructured CaP-coated implants have been compared to those of uncoated implants, using real-time reverse transcription (RT-PCR) [414]. At 2 weeks, the inflammatory response was suppressed and osteoprogenitor activity increased around the CaP-coated surface. However, at 4 weeks, progressive mineralization of the bone around the coated implant was observed, along with gradual resorption of the CaP coating. In another study [415], cellular and genetic characterization of nanocrystalline spherical HAp granules prepared by wet chemistry indicated that they may be attractive for bone repair.

Several studies have utilized NPs composed of calcium phosphosilicate, HAp, and composites of CaP and lipids or polymers to image with organic dyes and lanthanides, and to deliver oligonucleotides and a variety of drug molecules [416]. Calcium phosphosilicate NPs encapsulate and, therefore, provide *in vivo* protection for a variety of organic dyes and drug molecules [416]. HAp NPs have been shown to exhibit better fluorescence properties than their amorphous counterparts when doped with lanthanides because of rigid confinement of the lanthanide ions in the crystalline structure of these NPs [416]. Certain characteristics have been highlighted as requirements to ensure success for a NP system as a delivery agent, including colloidal stability in physiological conditions, small particle size (20–200 nm), lack of toxicity, encapsulation of active agent, targetability to cells of choice and effective clearance from the body [417]. HAp NPs have also been evaluated for potential transfer a green fluorescent protein (GFP) expressing plasmid into cell lines [418]. Subsequent *in vivo* infusion of these HAp NPs revealed no toxicity despite HAp uptake in the liver cells [419]. Hence, injectable CaP NPs hold great potential as delivery vehicles due to the ease with which proteins, drugs, and DNA can be attached. In addition, the availability of an agent that can be injected intravenously to the patient is a huge bonus as this easy procedure offers an opportunity to target systemic disease [419]. In a wish to develop better control of HAp nanocrystalline coatings on biomaterials, the interfacial interactions between calcined HAp nanocrystals and surface-modified substrates were investigated by measuring the adsorption behaviour and adhesion strength with QCM and atomic force microscope (AFM), respectively [420]. Rod-like HAp nanocrystals adsorbed preferentially onto anionic COOH-modified substrates compared to cationic NH_2_- or hydrophobic CH_3_-modified substrates. On the other hand, spherical nanocrystals adsorbed onto NH_2_- and COOH-modified substrates, which indicates that the surface properties of the HAp nanocrystals determined their adsorption behaviour.

In another study, nano-HAp was shown to be able to inhibit the growth of certain kinds of cancer cells, such as liver, throat and bone cancer cells, while having little side effect on normal cells [421]. The rate of HAp bonding to bone was demonstrated to be dependent not on the composition but on the release of calcium and phosphate ions from HAp, which determines the development of implant/bone interfacial strength [422]. The dissolution law of nano-HAp has been proven to be much different from that of conventional HAp. For nano-HAp, the dissolution is dominated by its particle size [393]. The particle-size effect is explained by the fact that small-sized particles of HAp may be degradable and stimulate bone ingrowth as they dissolve in the physiological environment [423].

**Figure 6:**
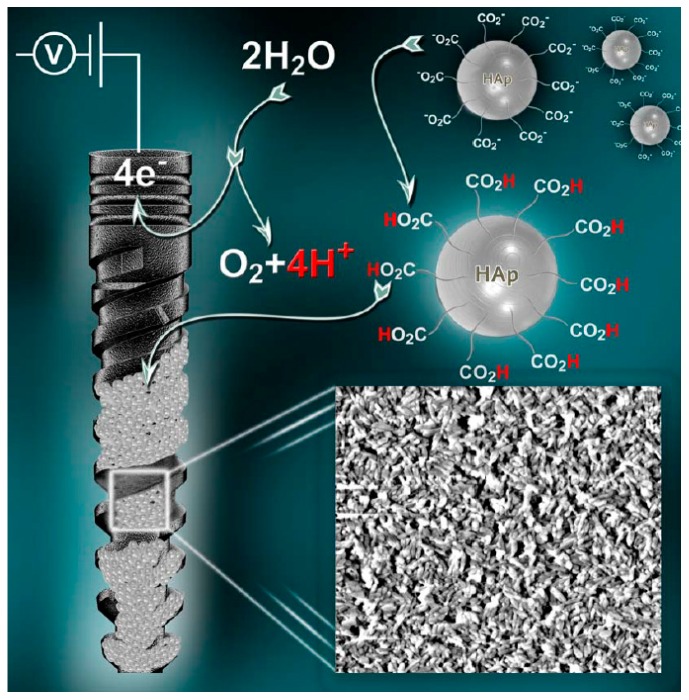
Schematic illustration of the novel approach for electrochemical deposition of pure HAp NPs for coating dental implants suggested by Mandler, Eliaz et al. in Reference s [424,425].

Electrodeposition of HAp can be carried out in two different manners, depending on the building blocks used in the process-ions or NPs. Starting with ionic species, i.e., calcium and phosphate ions, might form some undesired CaP phases in the coating. There is advantage in using NPs as the building blocks, thus dictating in advance the chemical composition and phase content of the resulting deposit and obtaining chemically uniform and predictable coatings. Moreover, nanoparticulate HAp possesses significant benefits, such as enhanced densification, improved fracture toughness, and increased osteoconductivity due to its high surface area. Mandler, Eliaz and co-workers [424,425] have recently presented a novel approach for electrochemical deposition of pure HAp NPs, e.g., for coating dental implants. The electrodeposition was successfully performed, using well-defined HAp NPs dispersed in aqueous solution using water soluble stabilizing agents, such as tri-sodium citrate (Cit) and sodium polyacrylate (PAA). Deposition produced high purity, single phase, HAp coating under both potentiostatic and galvanostatic conditions. The process is driven by applying positive potential, which oxidizes water, causing a reduction in the pH in vicinity of the implant surface. This results in the protonation of the carboxylic residues of the dispersants and diminishes the repulsion interactions among the NPs, thus driving irreversible aggregation of the particles. This novel process is illustrated schematically in Figure 6.

Considering all of the benefits described above, it is not surprising that during recent years, significant research effort has been devoted to nanostructure processing of HAp and its composites. The goal is to obtain structures with physical, mechanical, chemical, and biological properties better than their microscale counterparts and, at the same time, similar to natural bone mineral [57,121]. Processing of nano-CaPs has been reviewed in [426,427]. Some of the main processed for production of nano-HAp include solid state [428], wet chemical and sol-gel synthesis [429,430,431,432,433,434,435], hydrothermal [436,437], mechanochemical [438], pH shock wave [439], microwave processing [440], and sintering [441,442,443]. Different processes result in different shapes and sizes of NPs and, therefore, in different surface area. The crystal/aggregate size distribution and particle shape are known to affect their properties and, thus, their potential applications [444,445,446]. For example, the size and shape of the particles affect osteoblast proliferation, cellular activity and apoptosis, and osteogenic gene expression [444,447,448,449].

CaP NPs have been studied extensively as drug carriers. They have been evaluated as delivery vehicles for gene therapy [418], injectable carrier of various drugs [450], proteins [451], imaging purposes [419]. Iafisco et al. [452] demonstrated the use of CaP NPs as drug delivery systems for bone tumours. It should be kept in mind that NPs might introduce some toxicity risk *in vivo* through excess delivery of Ca^2+^ ions into cells [453].

## 8. Biphasic and Triphasic CaP Formulations

CaPs can form biphasic, triphasic, and polyphasic compositions, in which the individual components cannot be separated from each other [454]. The individual phases in such formulations are homogeneously and intimately ‘‘mixed’’ at the submicron level and, therefore, are strongly integrated with each other. Nevertheless, the presence of all individual phases is easily evident in the XRD pattern. As a rule of a thumb, the properties of biphasic, triphasic and polyphasic materials are between those of the constituent phases, and depend on the relative amounts of the ingredients. The Ca/P atomic ratios of BCPs typically fall between those for pure TCP and HAp. Thus, by changing the ratio between the more stable and the more soluble CaPs, it is possible to prepare formulations with adjustable properties.

Historically, the term biphasic calcium phosphate (BCP) was coined in 1986 [455] to describe a bioceramic that consisted of a mixture of HAp and β-TCP. Since then, BCP consisting of HAp and β-TCP has been the most studied among all known BCP formulations [454]. BCPs are a family of two-phase ceramics that combine the low solubility and osteoconductivity of HAp with the osteoinductivity of a more soluble phase such as TCP. A proper balance between the more stable CaP phase and the more soluble one is sought, so that the level of bioactivity, bioresorbability, osteoconductivity, and osteoinductivity can be adjusted [207,347,456]. BCPs may be produced by mixing HAp and TCP, or chemically by sintering CDHAs at high temperature to result in a mixture of two different phases.

To-date, it seems that only BCP formulations, and not triphasic or polyphasic formulations, are produced commercially (see Table 2 in [454]). These commercial BCPs are available as blocks, particulates (granules), and custom-designed shapes, such as wedges for tibial opening osteotomy, cones for spine and knee, and inserts for vertebral cage fusion [454]. They include different ratios of HAp/β-TCP, HAp/α-TCP, and more recently even β-TCP/α-TCP. Although triphasic HAp/β-TCP/α-TCP has been reported, it has not been commercialized yet, to the best of my knowledge. The BCP formulations are used as bone graft or bone substitute biomaterials for orthopedic, maxillofacial and dental applications under various trademarks [457]. They can be applied to large bone defects, in some load bearing areas, as customized pieces which will maintain their shape over long periods of time, in sinus floor elevation for dental implant placement, to fill dental root canals, etc. [252,454,457].

Arinzeh et al. [458] compared the kinetics of bone induction and the stimulation of osteogenic differentiation of human mesenchymal stem cells (hMSCs) for different HAp/β-TCP ratios (0, 20, 56, 63, 76 and 100 wt % HAp). The best results were obtained for 20%HAp–80%TCP. Silva et al. [459] described some of the *in vitro* phenomena regarding the effect of surface reactivity of BCP granules on human macrophages locomotion and secretion. Cells attached to BCP presented a higher intracellular free Ca^2+^ concentration compared with nonattached neighbours and secreted CaP particles into the medium. It was proposed that the secreted particles create a transition zone that allows further macrophage adhesion. He et al. [460] compared the *in vivo* response of calcium carbonate/phosphate-based glass (CC/PG) composite ceramic to that of porous BCP. Amirian et al. [461] fabricated a composite scaffold of gelatin (Gel)-pectin (Pec)-BCP for delivery of GFs. Bone morphogenetic protein-2 (BMP-2) and vascular endothelial growth factor (VEGF) were coated on the Gel-Pec-BCP surface to investigate of effect of them on bone healing. VEGF and BMP-2 loaded on Gel-Pec-BCP scaffold facilitated increased cell spreading and proliferation compared to Gel-Pec-BCP scaffolds. *In vivo* bone formation was greatest with Gel-Pec-BCP/BMP-2 scaffolds. Sadiasa et al. [462] coated simvastatin (SIM) drug incorporated poly(d,l-lactic-*co*-glycolide acid) (PLGA)/BCP composite on BCP/ZrO_2_ scaffold to enhance the mechanical and bioactive properties of the BCP/ZrO_2_ scaffold for bone engineering applications. The increase of PLGA concentration resulted in a lower release rate of SIM, which was claimed to enhance the performance of the scaffold *in vitro*.

## 9. Composite Calcium Phosphates

In Section 8, mixtures of different CaPs, not necessarily with clear microscopic interfaces, were reviewed. In Section 9, composite materials consisting of one or more CaP phase with other materials, either organic or inorganic, are described. The additional materials could be of biologic origin or synthetic, and they may be incorporated to enhance the mechanical properties or *in vivo* performance of the CaP-based material. FGMs will be reviewed in Section 10, while antibacterial CaPs will be reviewed in Section 11.

CaP coatings, cements and scaffold have long been integrated with both organic and inorganic materials for various reasons, such as control of the biodegradability and bioactivity, improvement of the mechanical properties or corrosion resistance, encapsulation of drugs or GFs, etc. A comprehensive review of calcium orthophosphate-containing biocomposites and hybrid biomaterials suitable for biomedical applications is given in [463]. Allo et al. [464] reviewed biodegradable nanocomposites and organic-inorganic hybrid biomaterials based on selective combinations of biodegradable polymers and bioactive inorganic materials, focusing specifically on nanocomposites based on nano-HAp and bioactive glass (BG) fillers in combination with biodegradable polyesters and their hybrid counterparts. Murugan and Ramakrishna [121] reviewed nanocomposites for bone grafting, focusing on HAp-based nancomposites (e.g., with collagen). Gremillard et al. [465] reviewed the degradation of bioceramics, including the clinically most widely used glass-ceramic, apatite-wollastonite (A-W), see Section 3.2.4 in [465]. Glass ceramics are made of a mixture of a glassy phase and crystalline precipitates. A–W glass ceramic is made of precipitates of apatite and wollastonite (CaO·SiO_2_) in a MgO–CaO–SiO_2_–P_2_O_5_ glass.

The use of CaP cements (CPCs), aimed at healing bone defects, is limited due to several reasons. Many properties can be improved by adding a polymeric phase, yet, the amount, type of polymer, etc., must be studied in order to tailor its effect on the end composite. The addition of polymers to cements have been used to alter their setting time, cohesion/washout resistance, injectability, macroporosity, mechanical properties, long-term degradation, drug eluting properties, and biological response [466]. Polymers can be added in the liquid phase as well as in powder phase, and may be either synthetic or natural. Examples for natural polymers are: alginate, chitin, chitosan, silk, hyluronate, cellulose, gelatin, soybean, albumen, collagen, and chondroitin sulfate. Synthetic polymers include polyethylene glycol (PEG), poly(ethyl) acrylate, polyesters and polyethers, polyacrylic acid (PAA), fibrin, PLGA, poly(glycolic acid) (PGA), polycaprolactone (PCL), poly-l-lactide acid (PLLA), armide fibres, polyamide fibres, etc. [466].

One of the problems of cements is their degradation rates that are un-matched with the growth of new bone [256], improper pore size and structure for bone ingrowth, and poor mechanical strength. Li et al. [467] studied a CaP cement composite with PLGA biodegradable polymer aimed to enhance the compressive mechanical strength and create controllable size pores. Ishikawa et al. [468] presented a composite of CaP with sodium alginate. This composite is aimed to overcome another problem of current cements—the decay of cement paste in contact with blood. Cao et al. [469] studied the mechanical reinforcement of injectable CaP cement reinforced with silk fibroin. It was found that the compressive strength was increased due to the combination with silk, while having no adverse effect on the injectability. Xu et al. [470] developed a layered CPC structure for moderate stress-bearing applications by combining a macroporous CPC layer that could accept tissue ingrowth with a strong, chitosan fibre-reinforced CPC layer. This functionally graded CPC enabled a relatively high strength and macroporosity to be simultaneously achieved.

Encapsulation in cements has also been shown. Ginebra et al. [471,472] reviewed the use of HAp and DCPD cements as drug delivery materials. Jain and Panchagnula [473] reviewed the applicability of various materials as bone fillers for Skeletal Drug Delivery Systems (SDDS). Roy et al. [474] showed a composite cement with PLGA aimed to encapsulate drugs. Zhang et al. [475] utilized a modified solid/oil/water emulsion solvent evaporation technique to prepare porous CaP composite cements containing simvastatin-loaded PLGA microspheres. The composite cements were found to effectively increase the cement’s biomechanical properties and absorption, and also improved the osteogenic activity through the simvastatin release. This composite scaffold provided a novel therapeutic approach for clinical treatment of bone defects. Yet, it is important to note that synthetic polymers, such as PLGA and PCL are often associated with unfavourable side effects, including inflammatory reactions, due to acidity of the degraded product [476]. Natural polymers are therefore a better choice as they induce minimal foreign body response (FBR). Other cements contain inorganic additives such as doped inorganic additives [477], carbon nano-tubes (CNTs) [478,479,480], and silicate-based cements [481,482,483]. Yet, those are still not very common. Li et al. [484] constructed a composite scaffold by combining mesoporous bioactive glass (MBG) and CPC. Recombinant human bone morphogenetic protein-2 (rhBMP-2) was facilely incorporated into this scaffold through a freeze-drying process. The resultant scaffold not only presented a hierarchical pore structure and a sufficient compressive strength, but also exhibited excellent drug delivery properties, presenting sustained release of rhBMP-2 for over 7 days. This composite scaffold presented a favourable effect on the proliferation and osteogenetic differentiation of bone marrow stromal cells (BMSCs). The incorporation of rhBMP-2 was found to induce a significant improvement of osteogenetic efficiency, especially in the early stage. Moreover, better biodegradability was obtained in the rhBMP-2 loaded MBG/CPC scaffold compared to the others. Verron et al. [485,486] reviewed the physical and chemical processes implicated in the preparation of drug-delivering CaPs. While their focus is on cements, coatings are referred to as well. Growth factors, antibiotics, antiosteoporotic drugs, chemotherapeutic drugs, and analgesic drugs were all discussed. CaPs have been laced with biological agents in order to induce osteoconduction and accelerate the healing process. Mostly, the biological agents incorporated are bioactive proteins, bioactive molecules, and GFs. Incorporation of GFs is important, providing the necessary signals for bone repair. These mainly include members of the BMP, VEGF, transforming growth factor (TGF), fibroblast growth factor (FGF), and insulin-like growth factor (IGFs) [485].

Blom et al. [487] studied the material properties and released characteristics of CPC incorporated with recombinant human transforming growth factor-β1 (rhTGF-β1). Cho et al. [488] studied the effect of platelet-rich plasma (PRP) on the osteoconduction of CPC and the bone strength of treated vertebra in an animal model. The activated platelets in PRP are known to release a high concentration of growth factors, such as platelet-derived growth factor (PDGF), TGF-β1 and insulin-like growth factor-1 (IGF-1), which play an important role in bone healing. The combination of CPC and PRP resulted in higher trabecular bone volume fraction, higher trabecular thickness, and higher bone mineral density (BMD), compared to either only CPC or PMMA. Yet, the authors explain that there had been a concern that the effect of PRP would be dependent on the species, and might show different results in humans. Other limitations that they raise deal with micro-computed tomography (µ-CT) analysis not being included in their study, as well as to a better biomechanical evaluation being required.

It should be noted that while GFs-containing CaPs continue to be the subject of extensive research, their clinical use is currently questionable. First, the improvement in osseointegration (of few percent) that can be achieved thanks to their addition to CaPs is considered insignificant by many orthopedists and dentists. More important, such GFs might have some dangerous side effects. The most famous case study is that of the Infuse bone graft (Medtronic, Memphis, TN, USA), which was once believed to be a game changer in bone surgery. This bone graft contained rhBMP-2 that promotes bone growth. It was approved by the US FDA in 2002 for use in lumbar (lower back) spinal repair surgeries, in 2004 for tibia repairs, and in 2007 for dental procedures. However, a growing number of individuals have suffered injury as a result of receiving this graft and subsequently filed lawsuits against Medtronic. This graft has been linked to unwanted bone growth (ectopic bone growth), bone and nerve injury, adverse back and leg pain events, bone resorption, implant displacement, infection, retrograde ejaculation in men, urinary retention, radiculitis, and possibly even an increased cancer risk. Consequently, already on 1 July 2008, the US FDA issued a Public Health Notification with the title “life-threatening complications associated with recombinant human bone morphogenetic protein in cervical spine fusion”. More details on the case study of Infuse are given elsewhere [489,490].

Qiao et al. [491] synthesized alginate-chitosan microencapsulated mouse osteoblast MC3T3-E1 cells in CPC. The novel injectable CPC-AC-cell construct was found promising for bone tissue engineering applications. Romeo et al. [492] synthesized two kinds of functionalized nanostructured hybrid microspheres, based on the bridged silsesquioxane family, by employing the sol-gel method via self-assembly of two different organic-inorganic bridged monomers. The architecture reached at molecular level allowed the incorporation of acetylsalicylic acid (ASA) as an anti-inflammatory model drug. The functionalized microspheres were proposed as delivery systems into CPCs, in order to slow down the characteristic drug-delivery kinetics.

CaP composite scaffolds have also been developed. The two main objectives of composite scaffolds are mechanical stability of the implant and encapsulation of various biological agents and drugs. In this case too polymers, both natural and synthetic, are vastly used. For example, Nouri-Felekori et al. [493] developed a composite scaffold of CaP-gelatin. Mixed forms of CaP particles, in the shape of whiskers or spherulites, were incorporated in the gelatin, thus influencing the mechanical properties of the implant. Another way to tailor mechanical properties such as brittleness of the CaP implant is by introducing collagen into the scaffold [494]. The implant is then strengthened partly owing to the energy dissipation that occurs through the covalent bonds formed between the collagen molecular chains in the scaffold [495,496]. This composite may also have a positive effect on bioactivity. For example, Li et al. [497] studied the influence of such a composite on adipose-derived stem cells. They showed that the composite resulted in better proliferation of the cells and osteogenesis-promoting effects. Ryu et al. [498] synthesized a natural bone-like peptide/hydroxyapatite nanocomposite with multi-level hierarchical structures. The organic matrix was prepared by coating self-assembled diphenylalanine nanowires with polymerized dopamine. Upon incubation in a simulated body fluid (SBF), polydopamine-coated peptide nanowires were uniformly mineralized with *c*-axis-oriented HAp nanocrystals, as observed in mineralized collagen fibres in natural bones. It was found that both the metal-ion binding ability and layered structure of polydopamine were responsible for the controlled heteroepitaxial growth of HAp nanocrystals along polydopamine-coated peptide nanowires. This nanocomposite could be readily hybridized with osteoblast cells at a higher hierarchical level. It was nontoxic and enabled efficient adhesion and proliferation of osteoblastic cells by guiding filopoidal extension. Li et al. [499] synthesized discrete high-aspect-ratio platelets of HAp and DCPD and combined these platelets with amyloid fibrils, as candidates to replace collagen, to generate a new class of hybrid nanocomposites, which show several bone-mimetic features. Hadisi et al. [500] presented bioactive nanobiocomposite scaffolds based on silk fibroin nanofiber-porous starch for potential bone tissue regeneration. Incorporation of silk fibroin nanofibers into the starch hydrogel was found to improve cell viability, proliferation, and attachment. Zhang et al. [501] fabricated macroporous CaP-chitosan composite scaffolds and evaluated them for use in bone tissue engineering. HAp scaffolds nesting chitosan sponges showed significantly higher alkaline phosphatase (ALP) level and osteocalcin (OC) production during the 11-day culture period, compared with chitosan scaffolds incorporated with HAp powders. The addition of CaP glass increased the ALP and OC levels of MG63 cells. The Hap-matrix composite scaffolds was claimed to enhance the phenotype expression of MG63 cells, in comparison with chitosan-matrix scaffolds. Zhang and Zhang [502] also fabricated macroporous chitosan scaffolds reinforced by either β-TCP or CaP invert glass. Both the compressive modulus and yield strength of the scaffolds were greatly improved. Reinforcement with β-TCP was found preferable w.r.t. apatite formation in SBF. Kozłowska and Sionkowska [503] studied the effects of different crosslinking methods on the properties of collagen-CaP composite materials. Collagen scaffolds with high porosity were prepared by the freeze-drying technique. CaP was incorporated by immersing the samples in CaP solution. It was concluded that 2% collagen concentration and crosslinking with carbodiimide (EDC/NHS) is the most promising combination for scaffolds in bone tissue engineering. Li et al. [504] fabricated a linearly graded, bonelike CaP coating on a nonwoven mat of electrospun nanofibers. Two biocompatible and biodegradable polymers were studied as fibres: PLGA and PCL. To improve the hydrophilicity of these two polymers and activate the surface for CaP deposition, their surface was modified by plasma treatment and/or gelatin coating. The CaP was deposited from 10 times concentrated SBF. Simon et al. [505] studied the *in vitro* cytotoxicity of zirconia-ACP filler, a copolymer matrix derived from the polymerization of a resin system, and the corresponding ACP composite. No adverse response regarding cell morphology and/or viability was observed with ACP composites compared to the unfilled copolymers or to the commercial adhesives. Maeda et al. [506] demonstrated bone healing by means of a sterilisable and osteogenic molecule-eluting implant system, in which a smoothened agonist (SAG) and a helioxanthin derivative (TH) were loaded onto tetrapod-shaped CaP granules (Tetrabone). Ethylene oxide gas (EOG) sterilization did not affect the osteogenic activity of the SAG- and TH-loaded Tetrabones. Okumura et al. [507] increased bone formation when incorporating bone marrow cells in coralline HAp.

Drug encapsulation is also widely used in complexed CaP scaffolds. For example, Zhang et al. [508] evaluated the encapsulation of BMP- and VEGF-loaded PLGA-CaP composite. Yaylaoğlu et al. [509] used CaP-gelatin composite for controlled drug release. Antibiotics has also been encapsulated in other polymers such as PCL, PLGA, and chitosan [510,511]. Zhang and Kataoka [512] reviewed some CaP-based nano/bio-composites as carriers for cellular delivery of therapeutic (i.e., DNA, siRNA, and proteins) and/or diagnostic agents, focusing on the PEGylated CaP delivery systems. Lin et al. [513] reviewed biomimetic coatings, including as carriers of growth factors and antibiotics (see Section 3 in [513]).

Coatings of CaP have also been shown to benefit from incorporation of various materials, both organic and inorganic. These often are used for corrosion control as well as improved bioactivity and encapsulation. For example, Ren et al. [514] studied CaP glass/MgF_2_ double layered composite coating for improved corrosion resistance of magnesium alloy. Such silica-free CaP glasses offer great potential for biomedical applications due to their good bioactivity and biocompatibility [515]. Huang et al. [516] fabricated by electrodeposition gelatin-containing and strontium-doped CaP composite coating in order to improve the corrosion resistance and bioactivity. Osteoblast *in vitro* tests demonstrated that the composite coating better enhanced the biocompatibility of Ti than CaP coating. CaP/zirconia composite has been shown to improve the corrosion resistance of magnesium alloys [517]. Su et al. [518] deposited DCPD and HAp coatings on HAp-Mg composites using a simple conversion coating method and a subsequent alkali post-treatment, respectively. The goal was to enhance the corrosion resistance and further develop the surface bioactivity of the composites to meet specific requirements of bone tissue engineering applications. The incorporation of zirconia has also been shown to form favourable mechanical and bioactive properties [519]. Incorporation of other inorganic materials, for example calcium titanate, has also been shown to improve mechanical strength as well as bioactivity [520]. Similarly to scaffolds and cements, coatings are also vastly combined with polymers. For example, Fan et al. [521] studied CaP-collagen composite coating that has shown to be highly bioactive. Liu et al. [522] studied the dissolution rate and mechanical strength of bovine serum albumin (BSA)-containing coatings as a function of protein concentration. Leonor et al. [523] studied the effects of BSA and α-amylase incorporation on CaP coatings. De Jonge et al. [524] found that the osteogenic potential of Ti can be stimulated by incorporation of enzyme alkaline phosphatase (ALP) in electrospray deposited CaP coatings.

Encapsulation of antibacterial agents and growth factors in coatings is also very common. Oyane et al. [525] demonstrated a composite coating consisted of CaP and ethylene-vinyl alcohol copolymer along with antibacterial agents such as lactoferrin, tetracycline and gatifloxacin. The antibacterial agents were immobilized on the copolymer and integrated into the CaP. The activity against *E. coli* and *S. Aureus* were demonstrated. Zhou et al. [526] synthesized ACP nanospheres by a co-precipitation method and used them to prepare CaP-poly(lactic acid) (CaP-PLA) composite. The as-prepared CaP-PLA composite was used to coat Ta plates and porous scaffolds. CaP-PLA coated Ta plates with BSA were prepared and used for the investigation of BSA release *in vitro*. VEGF and TGF-containing CaP-PLA coated porous Ta scaffolds were implanted in rabbit subchondral bone for defect repair.

Various articles have reported CaP coatings reinforced with graphene, CNT, or other carbons. Li et al. [527] fabricated graphene oxide (GO)-HAp coatings by cathodic electrophoretic deposition (EPD). The addition of GO into the HAp coating reduced the surface cracks, increased the coating adhesion strength and the corrosion resistance in SBF, and improved the biocompatibility *in vitro*. Janković et al. [528] also used EPD to obtain uniform bioactive HAp-graphene coating with improved mechanical strength and favourable corrosion stability in SBF. The composite coating was classified as non-cytotoxic when tested against healthy peripheral blood mononuclear cells (PBMC). Santos et al. [529] reported the one-step fabrication of a coating on ultrahigh purity magnesium using a parallel nano assembling process. The multifunctional biodegradable surface was obtained by adding HAp NPs to GO. By adjusting the relative contents of HAp and GO, the wettability could be tailored. The composite coating induced apatite formation. Liu et al. [530] used vacuum cold spray to fabricate composites HAp/graphene-nanosheet coatings. The addition of graphene significantly enhanced the fracture toughness and elastic modulus of the HAp-based coatings. Gao et al. [531] proposed a biomimetic (biomineralization) method for preparation of HAp/GO hybrid coating on AZ91 Mg alloy. GO was found to greatly promote nucleation and crystallization for rapid HAp growth, forming uniform and dense HAp/GO hybrid coating comprised by flake-like HAp crystals with Ca/P = 1.65. The corrosion current density of treated Mg alloys was decreased by one order of magnitude as compared to untreated Mg. Zanin et al. [532] applied electrodeposition to form globular nano-HAp on reduced graphene oxide (rGO). The carboxyl (carboxylic acid)/carboxylate functional groups attached directly to the rGO after oxygen plasma treatment were found essential to accelerate the OH^−^ formation and the deposition of globular nano-HAp crystals. The nano-HAp/rGO composites were shown to be an appropriate surface for MSC adhesion with active formation of membrane projections. Later, Zanin et al. [533] studied nano-HAp deposited onto vertically aligned multi-walled CNT scaffolds by electrodeposition and soaking in a SBF. The attachment of oxygen functional groups was found to be crucial for nano-HAp nucleation during electrodeposition. The purpose of this composite was to enhance the mechanical properties and to increase adhesion of osteoblasts while obtaining HAp which is more similar to the biological HAp than that obtained by other deposition techniques. Metoki et al. [192] reported a crystalline, needle-like nano-β-TCP electrodeposited on rGO nanosheets. The rGO was grown on CNT, as a composite biomaterial, in a one-step process, followed by electrodeposition of nano-CaP. It was speculated that the carboxyl (carboxylic acid)/carboxylate functional groups attached directly to the rGO are essential in accelerating OH^−^ formation and deposition of needle-like nano-CaP crystals. This composite presented an excellent *in vitro* biomineralization after soaking in SBF. In other studied too, HAp was deposited on graphene sheets [534], GO sheets [535], or functionalized graphene nanosheets [536,537,538]. Usually, the nanocomposites are synthesized as nanoparticles in solutions and should be separated by centrifugation. It is also difficult to control the microstructure of HAp crystals [539,540]. For flexible membranes containing HAp and polymers, on one hand, organic solvents have typically been used [541], which might be harmful to the cells and host tissues. Moreover, the ceramics are easy to be covered with polymers, restricting their exposure to the seeded osteogenic cells and decreasing the bioactivity of the composite scaffolds [542].

The use of CNTs, graphene and other carbon nanostructures for implants should be treated with caution. Yang and Webster [543] reviewed the biological responses to and toxicity of nanoscale implant materials. On one hand, carbon nanostructures are attractive for medical implant applications due to their extraordinary electrical, antiwear, and mechanical properties, and the capacity to promote the regeneration of various tissues and reduce immunological responses. On the other hand, health risks and toxicological data of these newly emerging carbon nanomaterials are not completely known. Different cell lines exposed to single-walled CNT (SWCNT) have demonstrated dose- and time-dependent apoptosis and inhibition of cell proliferation. Similar dose- and time-dependent increases in cell viability have been observed with multi-walled CNT (MWCNT). High concentrations (0.6 mg/mL) of MWCNT have also been reported to induce immune and inflammatory gene over-expression. Surface modification can reduce such high toxicity. A few groups have reported that surface modified, hydrophobic MWCNTs were less toxic than hydroxyl- or carboxyl-coated MWCNTs in the concentration range of 0.002–0.2 mg/mL. Many studies have attributed the cytotoxicity of CNTs to the internalization of NPs. CNT and other NPs have been shown to produce free radicals or cause oxidative stress, which further results in lipid peroxidation, DNA damage, cell membrane and cytoskeleton disruption, protein oxidation, and eventually, apoptosis or cell injury. A few groups have attributed CNT cytotoxicity to trace amounts of catalysts (e.g., Fe, Pt, and Y) remnant from manufacturing of these nanomaterials. Nanodiamond has also been emerged as a promising material for bioanalytical, drug delivery, and orthopedic implant applications. Most of the recent studies on nanodiamond have demonstrated low toxicity to various cell types and little production of reactive oxygen species [543].

## 10. Functionally Graded Calcium Phosphates

Functionally graded materials (FGMs) are materials that exhibit either chemical composition gradient or structural gradient within them [544]. This allows obtaining properties that cannot be achieved otherwise, for example exhibiting good biocompatibility—a surface governed property, with good mechanical strength—a bulk governed property. In the case of CaP coatings on metal implants, it can also reduce the failure rate due to delamination of the coating over time as a result of mismatch between the thermal expansion coefficients of the substrate and of the coating.

Biological structures lend insight into design concepts of new materials. When we consider the biological material, we often observe a number of design principles that are not usually used in traditional materials processing. One feature of biomaterials is the formation of hierarchical structures, such as described earlier. Furthermore, continuous changes of tissue composition and structure are vastly known in biology. For example, bone changes density from a stiff cortical bone outside to a trabecular cancellous bone inside. This functional gradation has been utilized by the body, and has been adopted as an approach for implant modification in the last two decades.

Pore-graded CaP scaffolds are example of FGMs. They are designed to meet both biological and mechanical requirements [545,546]. The idea is to have an outer layer of the scaffold designed to provide access for cells, blood vessels, and enhance bone formation, while the inner ceramic should improve mechanical strength [547]. For example, Werner et al. [548] demonstrated a pore-graded CaP scaffold that exhibited bending strength of approximately 50% higher than HAp scaffold with the same pore volume fraction but without gradient structure. Moreover, the scaffold was tested with osteoblast-like cells that formed a confluent layer on top of the scaffold as well as penetration into the pores [548]. Another use for such a scaffold is in the field of bioresorbable bone substitution, where graded implant porosity can grant a guided degradation progress and cell ingrowth [547]. For example, Schiller et al. [549] developed biodegradable functionally graded skull implants made of three layers. The implant basis was made of polylactide and CaP/calcium carbonate, while the inside consisted of macroporous and faster degradable PLA/calcium carbonate to allow ingrowth of bone cells. The outer layer consisted of slower degrading material to ensure mechanical stability as well as protection. Wehmöller et al. [550,551] developed and studied the mechanical properties of a functionally graded implant aimed to substitute the function of the skull for geometry and for protection of the brain. The implant consisted of polylactide/ACP as mechanically stable external structure and of polylactide/calcium carbonate as porous internal structure for the ingrowth of bone. *In vitro* bone tissue engineering also requires a functionally graded approach. Such an implant has to interact with osteoblasts as well as osteoclasts in order to initiate the remodeling of the scaffold. Therefore, such a composited scaffold must have graded pore distribution in order to control cellular activity [547]. An example is demonstrated by Linder et al. [552] who synthesized a β-TCP graded implant designed for *in vivo* degradation using a lost-wax casting technique.

FGMs may also have compositional change throughout the material. For example, CaP coatings on titanium could be made functionally graded system, in order to provide gradient of bioactivity and good mechanical strength [553,554]. Functionally graded CaP coating can be designed so that the top coated layer can provide CaP such as TCP or ACP for accelerated bone formation, while the layer underneath is a dense HAp layer with lower resorption rate and stronger bonding to the implant’s surface [555,556,557,558,559,560,561]. Some of these coatings can be considered as functionally gradient implants because the decomposition of HAp to TCP leads to BCP formation. Moreover, the decomposition of HAp in the presence of TiO_2_ leads to the formation of CaTiO_3_ impurity, and therefore to high adhesion strength [562]. Roy et al. [563] deposited TCP on commercially pure (CP) Ti by the Laser Engineering Net Shaping (LENS™) process, a directed energy deposition additive manufacturing process developed by Optomec (Albuquerque, NM, USA). A LENS™ 750 system, a laser power of 500 W, a scan speed of 15 mm/s, and a powder feed rate of 13 g/min were used to form a Ti–HAp composite layer on the Ti substrate. Pure HAp layer was created on top of the Ti–HAp composite layer. Ti-HAp composite coatings prepared using LENS™ were further deposited with a top layer of pure HAp using radio frequency (RF) induction-plasma spraying (PS) process. Phase analysis by XRD indicated phase transformation of HAp to β-TCP in the LENS™-processed coating. In LENS™-processed multilayer coatings, a compositionally graded nature was successfully achieved, but with severe cracking and a consequent decrease in the flexural strength of the coating. To obtain a structurally stable coating with a composition gradient across the coating thickness, a pure HAp layer was sprayed on top of the LENS™-processed single layer coatings using the PS process. The PS HAp coatings were strongly adherent to the LENS™–TCP coatings, with adhesive bond strength of 21 MPa. Based on *in vitro* tests, cellular activity reached a maximum in the case of LENS™-PS HAp coating. Marković et al. [564] fabricated nanostructured functionally graded HAp/Hap + β-TCP(BCP) by sintering. Farnoush et al. [565] formed functionally graded HAp-TiO_2_ nanostructured composite coating on Ti–6Al–4V substrate via electrophoretic deposition. Kumar and Wang [566] measured the elastic modulus and hardness of functionally graded HAp/Ti and HAp/α-TCP/Ti coatings. Cattini et al. [567] fabricated various bioactive glass/HAp coatings by the suspension plasma spraying (SPS) technique. Their microstructure, scratch resistance, and apatite-forming ability in a SBF were compared. The functional coatings included: (i) composite coating with randomly distributed constituent phases; (ii) duplex coating with glass top layer onto HAp layer; and (iii) graded coating with a gradual changing composition, starting from pure HAp at the interface with the metal substrate up to pure glass on the surface. The graded coating provided the best compromise between mechanical reliability and apatite-forming ability in SBF.

Functionally graded coatings can also assist in antibacterial activity. For example, Ag on coralline HAp has been shown to form a FGM [568]. Manjubala et al. [562] showed a functionally graded CaP scaffold composed of HAp, TCP, TiO_2_ and Ag_2_O in an attempt to improve the scaffold’s mechanical stability and antibacterial activity. Bai et al. [569] deposited a series of functionally graded HAp (FGHA) coatings incorporated with various percentages of silver for antibacterial components, using ion beam-assisted deposition.

Functionally graded CaP can also be utilized for mimicking interfaces, such as bone-ligament [504,570,571] or bone-cartilage [572]. Erisken et al. [572], for example, manufactured a graded scaffold made of PCL/β-TCP using a twin-screw extrusion for mimicking the bone-cartilage interface. Using this hybrid method, they were able to tailor a graded scaffold with β-TCP content of 0–15 wt %. Four weeks after seeding cells into the scaffold, they observed markers akin to the type of variations observed in typical bone-cartilage interface. Another example is the formation of such gradient of mineral for the formation of tendon-bone interface. Li et al. [504] formed such a scaffold with altered stiffness that further influenced the activity of preosteoblast cells. Another study [573] implanted a biphasic PLGA/CaP construct into mini-pigs for 6 months. Histology revealed excellent bone integration and a tidemark noted between cartilage and bone, with the chondral phase of all samples producing mineralization near the tidemark. However, integration with surrounding cartilage tissue was poor.

## 11. Antibacterial Calcium Phosphates

One of the major concerns of all implants is the risk of infection [574], which is estimated in the range of 0.5%–5.0% for total hip arthroplasty [575]. Similarly, in the field of dentistry, a condition well known as “peri-implantitis” is found to be common [576]. It is mainly caused by infection around the implant, which leads to loss of supporting circumferential bone, causing its failure [576]. Although infections associated with prosthetic joints occur less frequently than aseptic loosening failures, they represent the most devastating complication. Implant-associated infections occur either by direct inoculation into the surgical wound during surgery or immediately thereafter during the first post-operative days (perioperative infection), by microbial spread through blood from a distant focus of infection (haematogenous infection), by direct or lymphogenic spreading from an adjacent infectious focus, or as a result of penetrating trauma (contiguous infection). About one-third of the infections develop within 3 months, another third develop within 1 year, and the remainder develop more than 1 year after surgery. The Gram-positive *S. aureus* (~2/3 of chronic osteomyelitis cases) and *S. epidermis* are the most common pathogens. These germs are part of the natural skin flora of humans and occur ubiquitously. Removal and replacement of the prosthesis are usually required to eradicate the infection. Thus, bacterial infections pose a significant clinical and financial burden in both diagnosis and treatment. They tend to serious relapses, cause trauma to the patient, and might impose disability and life threat [577,578,579,580,581,582,583,584,585,586]. Most infections are due to contaminations adhering to the implant surface during surgery and involve the formation of a biofilm [587]. This biofilm covers the implant surface and protects the bacteria from environmental attacks and systemic antibiotic [588]. Most of the infections develop from an early contamination that occurs during the operation or in the first few days after surgery. Events such as these, which become symptomatic or anyway manifest shortly following surgery, within 3 months of implantation, have been referred to as “early” infections [589].

To be used as a drug carrier, the potential substance must have the ability to incorporate a bioactive agent either physically or chemically, retain it until reaching the specific target site, be gradually degraded, and deliver the active agent in a controlled manner over time [590]. All these criteria are well met by CaPs and their composites. In order to make the implant antibacterial, various antibiotics have been incorporated in CaPs [510,511,525], for example, gentamicin [591,592,593,594,595,596], ibuprofen [597,598], cephalothin [593], amoxicillin [593], tobramycin [593], zoledronate [599], aspirin [600], flomoxef sodium [601], tetracycline [602,603], vancomycin [604,605], streptomycin [606], etc. Tetracylines have a broad spectrum of antibiotic action and, therefore, are attractive candidates.

Rajesh et al. [594], for example, studied pulsed laser deposition (PLD) of HAp on titania nanotubes containing gentamicin. The drug was inserted through dipping and vacuum drying after the formation of the coating. Luginbuehl et al. [603] added tetracycline antibiotics into different polymer solutions and sprayed them onto TCP-coated surfaces. They showed cumulative release of antibiotics over an extended period (up to 70 days). Radin et al. [604] loaded CaP coatings with vancomycin by immersion. This loading showed effective release and inhibition for the first 24 h. Baro et al. [595] mixed gentamicin with PLA and CaP paste to form a CaP powder containing antibiotics. The powder was pressed onto implant surfaces, which resulted in long release durations (up to 12 weeks). Fu et al. [606] demonstrated a dual step electrochemical deposition, whereby they first deposited HAp, and then streptomycin on top of it. It was shown that the loading was more substantial than physical adsorption of the drug. Moreover, 80% of the drug was released during the first 24 h. Gentamicin is an antibiotic of the aminoglycoside family, which is extensively used in the context of CaPs. Therefore, antibacterial implants containing gentamicin have been prevalently studied by numerous research groups using a variety of coatings, cements, scaffold, and loading methods. The topic of antibiotics-incorporated CaPs has been reviewed in details elsewhere [485,486,513,607,608].

There are different approaches for the incorporation of antibiotics in the CaP, including in situ deposition [596], mixing powders during synthesis of scaffolds and pressed coatings [595], absorption in microspheres during CaP synthesis [609], covalent protein immobilization in microspheres [610], dip-coating [611], co-precipitation [611], etc. The carriers include, among others, chitosan [609] and gelatin [612]. CaP coatings have been applied most commonly by PS technology. One prominent drawback of this process is the inability to incorporate organic compounds, such as antibiotics, during the coating process due to the extremely high processing temperatures [613]. Therefore, in order to incorporate antibiotics into such coatings, a post-treatment has been implemented, usually by physical absorption [613]. For example, Stigter et al. [614,615] incorporated a variety of antibiotics into biomimetically prepared carbonated HAp coating using an immersion technique. They showed that some antibiotics were better incorporated, depending on their chemical structure, based on release studies. Moreover, they showed that the release rate differed between the antibiotics, reaching only one-day release for gentamicin.

Despite different studies with positive results, no commercial antibiotics-releasing CaP coating is already marketed, to the best of our knowledge. This may be either due to the high temperatures involved in some of the HAp deposition technologies, or due to the low porosity of many implants, which limits the antibiotic load onto their surface. In addition, stabilization of the HAp coating for prolonged drug release has often been sought by addition of PLLA, PLGA, or lipids, but these might have an adverse effect on the properties of the HAp surface *in vivo*. It should be noted that unlike coatings, there are already several commercial antibiotics-eluting CaP cements, mainly of European companies.

Physical absorption of antibiotics and other molecules onto the surface of CaPs limits the amount loaded and release kinetics. Antibiotic loading by a dipping method leads to a burst release of the antibiotics, such that more than 80%–90% of the antibiotics are released from the CaP coating within the first 60 min [605]. Therefore, it is better to develop a method whereby the coating is carried out at significantly lower temperature and the drug is added continuously during the deposition.

Altomare et al. [586] reported the in situ electrophoretic deposition of CaP with antibiotics. In that study, fairly high cathodic current (20 mA/cm^2^) was applied for 240 s at room temperature. The authors report an effective incorporation of gentamicin without any major impact to the morphology, density and structure of the brushite coating. Furthermore, they claim that the coating showed antibacterial efficacy on different streptococcal strains, using an inhibition zone. Yet, the amount of antibiotics present in the coating was not tested, and no release studies were made. Thomas et al. [616] demonstrated an innovative in situ electrodeposition of gentamicin-loaded chitosan NPs along with CaP, at low potential and current. The coating consisted of both ACP and β-TCP. A high drug loading into the coating (up to 42 wt %) and a controlled release of the drug over two days were demonstrated. Increasing the bath temperature did not result in an increase of the amount of gentamicin being deposited. Yet, it changed the internal pore size inside the coating, which may influence the *in vivo* behavior of the coating. While this has been an important step towards a good drug eluting coating, the antibiotic release was too short. A local inhibition release profile should exhibit a high initial burst in order to respond to the elevated risk of infection post-surgery, yet, it must also follow a sustained release for inhibiting the occurrence of latent infection [617]. Moreover, the amount of antibiotic released was 40% of the drug loaded, and the deposition time was relatively long (2 h). This prolonged deposition time does not suit the industry, and no mechanism was discussed for the partial release. Hence, Thomas et al. [618] next presented a novel, fast (30 min) and efficient, in situ galvanostatic (*i* = 0.6 mA/cm^2^) electrodeposition of CaP with chitosan NPs containing antibiotics. The deposited layer of OCP and DCPA contained a large amount of gentamicin, which was released gradually over a period of 15 days. In addition, both the cytotoxicity and biomineralization of the coating were studied, and the coating was proven to be non-cytotoxic and highly biomimetic. Mandler, Eliaz and co-workers [424,425] have recently presented a novel approach for electrochemical deposition of pure HAp NPs. These NPs can be pre-loaded with antibiotics (unpublished data), thus engineering a drug-release coating in a fairly simple way.

Using antibiotics as the antibacterial agent raises the concern that the balance of germs in the body will be affected, and that antibiotics-resistant bacteria will develop. The combination of different drugs in the implant poses a greater risk. Lower immunity of sick patients coupled with the escalating problem of antibiotic-resistant pathogens has driven increased rates of infection in hospital and surgical environments. Consequently, in particular in the USA it is becoming harder to obtain new regulatory approvals of antibiotics-releasing implants. One, potentially attractive alternative, is to incorporate antibacterial ions and NPs in the CaP.

For example, silver has long been known for its antibacterial properties [569,575,576,619,620,621,622,623,624,625,626,627,628,629,630,631,632,633,634,635,636,637,638,639,640], when it is in its ionic (Ag^+^) and not elemental form. Silver-based antibacterial surfaces must release silver ions directly into the pathogenic environment to be effective. Silver and most silver compounds have an oligodynamic effect and are toxic for bacteria, algae, and fungi *in vitro*. Among the elements that have this effect, silver is the least toxic for humans. Silver ions have antibacterial properties for a few reasons: (1) They can interfere with cell DNA and affect their ability to procreate; (2) They can inhibit enzymes involved with respiration, essentially suffocating the bacteria cells; and (3) They can react with sensitive thiol groups on bacterial proteins to destroy normal biological activity of the protein. The multi-modal activity also makes it difficult for bacteria to develop resistance in the same way they do to specific antibiotic medications. Silver exhibits low toxicity in the human body. Silver is absorbed into the human body and enters the systemic circulation as a protein complex to be eliminated by the liver and kidneys. Silver metabolism is modulated by induction and binding to metallothioneins. This complex mitigates the cellular toxicity of silver and contributes to tissue repair. Though toxicity of silver is low, the human body has no biological use for silver, and when inhaled, ingested, injected, or applied topically, silver will accumulate irreversibly in the body, particularly in the skin, and chronic use combined with exposure to sunlight can result in a disfiguring condition known as argyria in which the skin becomes blue or blue-gray. While argyria is usually limited to skin discoloration, there are isolated reports of more serious neurologic, renal, or hepatic complications caused by ingesting colloidal silver. Some people are allergic to silver, and the use of treatments and medical devices containing silver is contraindicated for such people. Bone toxicity is not widely recognized in the safety evaluation of silver and silver-containing products, but there are strong indications from *in vitro* models that Ag^+^ interacts with and binds to the HAp complex and can displace calcium and magnesium ions. Other research has demonstrated that Ag^+^ induces calcium release from the sarcoplasmic reticulum in skeletal muscle by acting on the calcium-release channels and calcium-pump mechanisms, presumably through oxidizing sulphydryl groups. Although this suggests that bone and possibly cartilage are vulnerable to prolonged release of Ag^+^ used as an antibiotic in bone cements, orthopedic pins, dental devices, and so forth, this has not been established so far [619,620]. While there are several startup companies in Europe that have developed silver coatings on medical devices, some orthopedists and dentists have raised concerns regarding the possible adverse effects of silver and the transport of silver ions in the human body to the lymph nodes. Other claims are that amalgams are being less used not only because of mercury, but also because of silver, and that silver does not provide absolute resistance against germs (e.g., infections are often found underneath dental fillings).

Other antibacterial ions and NPs can be incorporated in CaPs instead of silver, for example ZnO NPs [641,642]. However, Yang and Webster [543] reported that ZnO NPs have apparent toxicological effects. These NPs have been shown to produce free radicals or cause oxidative stress, which further results in lipid peroxidation, DNA damage, cell membrane and cytoskeleton disruption, protein oxidation, and eventually, apoptosis or cell injury. Nevertheless, detailed mechanisms of reactive oxygen species (ROS) generation by exposure to NPs remain unclear [543].

## 12. The Effect of Sterilization

All implants must be sterilized before implantation in the human body to avoid subsequent infections. The sterilization technique assigned must have minimum adverse effect on the material [643]. Among the methods used to sterilize implants, steam sterilization (autoclaving), dry oven, ethylene oxide (EtO), and isopropanol diluted in water (60–90 vol %), with or without ethanol, are the simplest, cheapest and most commonly available [643]. Yet, these have shown to degrade various CaP phases. Other sterilization methods include gamma irradiation, laser irradiation, ultraviolet (UV) irradiation, and plasma cleaning [644,645,646,647,648]. For different kinds of biomaterials and implants the sterilization technique may be different. Steam sterilization is one of the most common methods employed in the biomedical field because it is non-toxic and relatively easy to control [649,650,651]. It has also been widely applied to CaPs [652,653].

Several studies have shown the influence of the sterilization conditions on the properties and stability of various CaP phases at different conditions. Li et al. [654] studied the influence of steam sterilization on the physiochemical properties of porous CaPs. These are more susceptible to environmental damage than dense structures due to their large surface area and abundant active sites. Various phases were studied, including β-TCP, HAp and BCP. It was found that steam sterilization affects the different phases differently, and depending on the phase content. The morphology and mechanical strength of β-TCP were altered, and BCP showed significant changes in morphology, phase composition and dissolubility. HAp showed increased solubility and mechanical strength. Dorozhkin et al. [649] investigated the effect of steam sterilization on DCPD, CDHA and BCP in suspension. It was found that sterilization resulted in dehydration of DCPD and hydration of calcium oxide incorporated into the BCP. Moreover, the pH of the solutions changed significantly. Yet, only minor changes were found in the case of CDHA. Santos et al. [655] investigated the effect of steam sterilization on HAp NPs. NPs prepared by different processes were differed in size, shape and physicochemical properties. The sterilization modified markedly the shape, size and aggregation state of NPs produced by wet chemical synthesis. In contrast, NPs produced by hydrothermal synthesis showed only minor changes in chemical composition.

Other sterilization techniques have been applied to CaPs as well. Ethylene oxide gas sterilization might leave residues of EtO within the pores of the CaP [656], despite the fact that it has been used for CaP [506]. Gamma irradiation has been widely used in the case of CaPs. For example, Miyamoto et al. [657] sterilized TTCP/DCPA-based CaP cement powder by exposure to 20 kGy gamma irradiation. Lebugle et al. [658] sterilized a powder mixture of CaP, dextran and methotrexate, using 32 kGy gamma ray. Lakstein et al. [92] vacuum bagged and sterilized HAp-coated Ti–6Al–4V rods, using a dose of 30 kGy (3.0 Mrad). Gamma irradiation was preferred over EtO gas because the latter might lead to coating detachment and requires a long rest time for degassing. Wang et al. [656] studied the effect of gamma irradiation on TTCP/DCPA-based CaP cement. The setting time, compressive strength, phase conversion rate and morphology of the cement were all related to the dosage of gamma ray sterilization. The best dosage was 30 kGy, which led to slightly longer setting time, the highest phase conversion rate, and the highest compressive strength. Suwanprateeb et al. [647] showed that the use of gamma irradiation yields increased creep resistance that is associated with the formation of crosslinks and an increase in crystallinity.

Several studies also compared the effect of different sterilization techniques on CaPs. Takechi et al. [646] studied the effect of steam, dry heat, EtO gas, and gamma irradiation sterilizations on the setting and mechanical properties of CPC. In the case of steam sterilization, the powder aggregated before setting-time measurements. On the other hand, when the powder was sterilized by dry heat or EtO gas, the setting time was prolonged significantly and the tensile strength values of the cement decreased significantly. Accordingly, the following experiments focused on gamma sterilization. The setting time of the CPC was retarded and the tensile strength decreased when the gamma irradiation dose was increased, although no compositional change was found. Morejόn-Alonso et al. [643] also studied the effect of different sterilization methods on the physical, chemical and mechanical properties of CPC. The cement was composed of CDHA, OCP and β-TCP. Partial decomposition of the OCP was observed after steam sterilization, dry heat, and EtO sterilizations. In addition, the mechanical strength decreased in the following order: EtO, dry heat, and steam sterilization. Several compositional and microstructural changes were detected after dry heat and steam sterilization. Ethylene oxide sterilization had lesser effect on the chemical composition and strength than dry heat and autoclaving. Zahraoui and Sharrock [659] developed polyester copolymers, including lactic acid moieties, and studied the effect of sterilization on the physico-chemical properties of derived bone biomaterials. Chitosan solutions showed a dramatic decrease in viscosity after 25-kGy gamma sterilization. Aqueous copolylactic solutions also showed that hydrolysis occurs to liberate monomers after 25-kGy gamma sterilization. Heat sterilization also degraded chitosan solutions. However, apatite-copolylactic solids could be steam-sterilized without deterioration. Ultrafiltration was applied to prepare aqueous copolylactic solutions without polymer hydrolysis.

## 13. *In Vitro* and *In Vivo* Tests

Numerous *in vitro* and *in vivo* studies have been carried out with CaPs. In the previous Sections, references were already given to some of these *in vitro* [237,238,413,459,491,498,504,505,510,516,524,527,548,550,551,563,571] and *in vivo* [148,189,207,210,212,213,214,261,264,305,333,398,409,419,460,491,508,595,642,652] studies. Therefore, the purpose of this Section is not to try reviewing them, but rather focus on some of the major general principles, in our opinion.

*In vitro* tests are commonly used to characterize the bioactivity, biomineralization, cell proliferation and differentiation, protein adsorption, cell toxicity, drug delivery, corrosion behaviour, etc. The re-precipitation of bone and the corrosion of metal and CaP-coated implants are often tested *in vitro* in SBF [660,661,662,663,664,665,666,667]. Compositions of selected SBFs are listed in Table 7. As can be noted in this table, the composition of SBF was varied over the years to better match that of human blood plasma. The original SBF, used by Kokubo et al. [663] and Hench et al. [664] lacks the $SO_{4}^{2-}$ anion. A revised SBF (r-SBF) was proposed to adjust the ionic concentrations of $Cl^{-}$ and $HCO_{3}^{-}$ [662]. Another improved SBF (n-SBF) reduced the $Cl^{-}$ ion concentration to the level of human blood plasma, leaving the $HCO_{3}^{-}$ ion concentration equal to that of the corrected SBF (c-SBF) [665]. It is also common to use more concentrated SBFs (e.g., five times the concentration of SBF) whose goal is to accelerate biomineralization. It should be noted that the solution concentration (therefore, the solution supersaturation and crystallization kinetics) strongly influences the structural properties of the CaP precipitate formed. Therefore, more precise simulations of the human body fluids are obtained with un-concentrated SBFs.

*In vitro* testing with cell cultures mostly gives very specific answers. Since there is no general standard for these tests, for example, type of cells, passage number of the cells, culture medium, the use of extracted cells vs. cell lines, etc., the results are hardly comparable. Although some conclusions can be drawn from such experiments, saving animal lives and costs, one should note that *in vivo* cell behaviour might be different than that *in vivo* since many factors govern the cells behaviour in the body. For example, *in vivo* there are many cell types cooperating together by hormonal signalling. Furthermore, the turnover of the interstitial fluid plays an important role for the accumulation or dilution of drugs/ionic concentrations. One should also take into account that the surface tested *in vitro* may not be the one present *in vivo*. Moreover, on surgical insertion of the implant, the pH of the body fluid in vicinity of the implant drops from the normal value of 7.4 to 5.5, and in the course of 10 to 15 days regains neutrality [667].

*In vivo* testing in animal models is extremely important in order to understand the processes occurring in a living system. The spectrum of animals used for CaP testing is rather small, and includes mainly rats, rabbits, dogs, goats and sheep [49]. Usually, only one animal model is used to prove a hypothesis. The various animal models are usually used for different purposes. Rat or mice models are usually used for subcutaneous examination of an implant [494,668], while rabbit models are the easiest way to examine a coating’s interaction with femoral bone [92,460,669]. On the other hand, large animal models such as dog, sheep and goats are used to verify the practicability of the implants closer to the real clinical situation. Usually, the implants are then inserted in either the femur, tibia, or mandible bones [670]. Dogs are usually used as a model of dental implants [671]. Though some work has been done on ectopic sites (as described earlier), most studies focus on the biological response of the living bone to CaP.

Although *in vivo* tests are prevalent, the parameters of experiment are not standardized. For example, implantation times has varied significantly. Whereas in earlier experiments the implantation time was considered to be as long as months, and preferentially years [672,673], more recent experiments consider mainly short-term tests [202,667,669]. The most common time range in current literature is between one week or less to several months after implantation. The site of implantation also varies. The implants are then characterized by different techniques, such as histological sections and pullout tests [92,674]. While pullout tests have one purpose—to determine the bonding strength between the newly formed bone and the implant, histology can be used for many purposes, such as measurement of the new bone area (NBA), bone apposition ratio (BAR), labelling of various cells, etc. [92,202,460,669]. Moreover, other parameters can be examined in the living tissue before sacrifice, such as cell migration to the implant’s surface or drug release [669].

The properties of CaPs, in particular surface properties, affect the cascade of biological processes (protein adsorption, cell adhesion, and cell differentiation). These include surface roughness, Ca/P ratio, level of crystallinity, solubility, surface charge/energy, grain/particle size, etc. These have been reviewed in [228,252,675,676]. Protein adsorption, for example, is affected by surface roughness, microporosity, surface charge, ionic environment, and solubility, among others. Surface roughness has a size-dependent effect: features (namely, average roughness, *R*_a_, and grain size) smaller than 100 nm appear to promote protein adsorption better than features larger than 100 nm. High microporosity favours protein adsorption due to high specific surface area of the CaPs. Adsorption appears to be insensitive to pore sizes for diameters larger than 20 nm. Protein adsorption is influenced by electrostatic interactions between the protein and the surface, as well as by the charge and structural stability of individual proteins. Crystalline and stable CaPs appear to influence adsorption via charged sites and structural rearrangement of proteins, while amorphous and soluble CaPs influence adsorption by causing changes in local pH and ion concentration. Cell adhesion is affected, among others, by surface roughness, surface charge, solubility, and crystallinity. Cell adhesion is thought to be disrupted on surfaces that exhibit nano- or submicron-scale roughness. However, the specific effects of roughness (both at the nano- and micron-scale) on cell adhesion are inconclusive due to contradicting data in the literature. High crystallinity and low solubility appears to favour cell adhesion. CAPs also influence cell behaviour by modulating the ionic environment and changing the concentration of ions in solution via adsorption/leaching mechanisms. Finally, cell adhesion seems to be facilitated by the direct adsorption of negatively charged cell-adhesive proteins on positively charged surfaces (e.g., cationic calcium sites on CaPs) [228]. Some further *in vitro* [462,673,677,678,679,680,681,682,683,684,685] and *in vivo* [211,680,686,687,688] results are available elsewhere.

Eliaz et al. [237,238] summarized some aspects of the interaction of HAp coatings with bone-forming cells. Baier et al. [689,690,691] reported that high surface energy (hydrophilic) metal implants were associated with an enhanced fibroblast cell response. Aronov et al. [231] tuned the wettability of HAp (10° < θ < 100°) by an innovative post-treatment of exposure to low-energy electron irradiation and found that DNA tended to bind to surfaces with θ < 50°. In contrast, BSA protein, which contains hydrophobic domains, bound preferentially to surfaces with θ < 50°. The surface energy has also been shown to affect the bone cell maturation and differentiation [232] and the osseointegration [233]. Eriksson et al. [234] found that the cellular reaction was different for hydrophilic and hydrophobic implants, especially in the initial stages of wound healing. Surfaces with a higher surface energy showed more rapid cell activation and differentiation than those with lower surface energy. Eliaz et al. [237] observed that osteoblast progenitors seemed to attach preferentially to HAp post-treated to θ = 30° or 90°, although this tendency was not statistically significant. A preferential attachment to HAp surface post-treated to 30° was observed when MBA-15 osteogenic cell line was used instead of osteoblast progenitors derived from rats. The adhesion and proliferation of osteoblasts have been correlated with substratum wettability, the cells exhibiting a strong preference for hydrophilic substrata [235]. Such a preference was also observed by Eliaz et al. [238]. It should be borne in mind, however, that when relating the relative biological interaction with surface energy, there may be an optimal biocompatibility zone with respect to the critical surface tension (or surface free energy) [692]. It should also be noted that the conversion of contact angles to surface energies is not straightforward in the case of porous surfaces, like the certain HAp coatings and scaffolds. In this case, models such as those proposed by Wenzel and by Cassie and Baxter must be considered [692,693,694].

**Figure 7:**
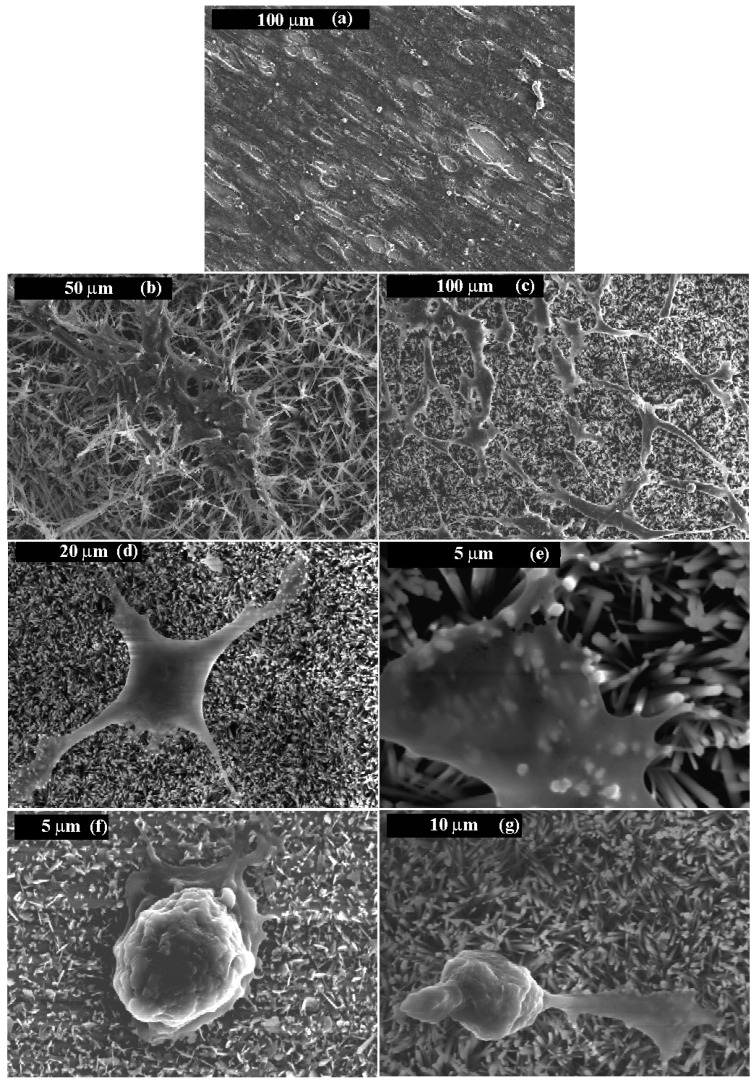
SEM images of MBA-15 osteogenic cells on surfaces of: (**a**) Gr-Ti; (**b**,**f**) HAp4.2; and (**c**–**e**,**g**) HAp6.0 [238]. Reproduced with permission from Elsevier Ltd.

Eliaz et al. [238] compared the viability of MBA-15 cells on HAp electrodeposited either at pH = 4.2 (HAp4.2) or at pH = 6.0 (HAp6.0) to that on ground Ti (Gr-Ti). Figure 7 demonstrates the typical cell coverage and cell morphology on each of the three surfaces. The area covered with cells is larger on HAp6.0 than on HAp4.2, implying that the former is more bioactive. On Gr-Ti (Figure 7a), a confluent cell layer covers the surface, masking most of the grinding grooves. The cells are flattened and well spread, forming network, with almost no filopodia and lamellipodia visible. A correlation is observed between the orientation of the grooves and the orientation of the cells, a phenomenon known as “contact guidance” [695,696]. The cells spread much less on HAp4.2 and mostly have a stellate shape (Figure 7b). Some cells retain the ellipsoid shape of the nucleus, with a diameter of ~7 μm, while beginning to form circular lamellipodia and spidery filopodia (Figure 7f). Fewer rounded cells are observed on HAp6.0 (Figure 7g) compared to HAp4.2. Instead, the cells are highly stretched on top of needles of the HAp coating and exhibit many focal contacts (Figure 7c–e). At high magnifications (Figure 7), the cytoplasmic membrane seems to be very thin, bridging over the coating protrusions, except where filopodia penetrate into pores and grasp the needles to assist in stretching and morphological changes of the cell.

Eliaz et al. [238] related the difference between HAp6.0 and HAp4.2 to the findings that the HAp6.0 coating was both richer in Ca and more crystalline than HAp4.2. It is well known that the crystallinity of a biomaterial surface affects specific cell responses such as the organization of cytoskeleton filaments and cell proliferation mechanisms. Spreading of osteoblasts, for example, has been reported to be faster on more crystalline surfaces, mainly due to the development of a more organized cytoskeleton [697]. During cell culture, extracellular matrix (and other) proteins adsorb onto the biomaterial surface and help in the subsequent cell attachment. In the case of osteoblast culture in serum, fibronectin and vitronectin are two important proteins that affect the cell attachment and spreading [698,699]. It has been reported that calcium ions in HAp, which form sites of positive charge, aid the adhesion of these two proteins, thus promoting the attachment of osteoblasts [700,701]. Other researchers have also reported the positive effect of calcium ions on cell growth [702,703], in contrast to the inhibitory effect of phosphate ions on cell activity [704].

Cell motility is generally associated with the protrusion of two types of actin-rich structures, namely lamellipodia and filopodia, at the leading edge of migrating or spreading cells [705]. These focal contacts serve as coordination sites between cell adhesion and motility; enhanced focal adhesion is associated with reduced cell motility. The elongation and reorientation of filopodia may be determined by guidance cues from the environmental signalling, e.g., the biomaterial surface structure [706,707]. From Figure 7 it is evident that the osteoblastic cells had more enhanced motility on HAp6.0 than on Gr-Ti. The morphology of flattened cells with numerous filopodia is similar to that reported elsewhere for osteoblasts on CaP [708,709]. The phenomenon reflected, for example, by anchorage of actin fibres at the periphery of artificial pillars where focal contacts form and stretch from one pillar to the next was termed ‘‘topographical compensation” or ‘‘gap guidance” [710]. Regarding the behaviour of cells on Gr-Ti, it is well known that, in response to microgrooved surfaces, cells may elongate in the direction of the grooves and be travel-guided by them. This phenomenon has become known as ‘‘contact guidance” [695,696,711,712,713]. The groove depth was shown to be much more important than the spacing between grooves in determining cell alignment, which increased with depth [711].

With respect to the effect of surface roughness, the latter is commonly expressed in terms of amplitude parameters such as the mean roughness *R*_a_ and the root-mean-square roughness *Z*_rms_ [38]. However, Eliaz et al. [238] found that the use of these amplitude parameters to characterize porous coatings resulted in inconsistent, misleading conclusions. Two alternative texture parameters were found more reliable. The developed interfacial area ratio *S*_dr_ reflects the additional surface area contributed by the texture compared to a totally flat sampling plane. This hybrid parameter can be useful in applications involving surface coatings and adhesion, or when considering surfaces used with lubricants and other fluids. The core fluid retention index *S*_ci_ is derived from the bearing area analysis of the complete three-dimensional surface. It is a measure, relative to *Z*_rms_, of the volume of fluid that the surface would support from 5% to 80% of the bearing area [714]. The effect of roughness on cell attachment has been demonstrated [236,695,698,711,715,716]. It was shown that the surface roughness must be within the scale of the cell to be perceived by the cell, and within this limit, rougher surfaces support the expression of a more differentiated osteoblastic phenotype based on increased alkaline phosphatase activity and osteocalcin production [715]. It was also reported that increased surface roughness, both at the micrometre and at the nanometre levels, without changes in surface chemistry, could promote functions of osteoblasts, leading to new bone synthesis [716].

Eliaz et al. [92,189] have summarized different aspects related to *in vivo* studies of CaP-coated implants. Regarding the implantation period, it has been argued that healing periods longer than 4 weeks do not further increase the quantity of bone ingrowth into implants with porous surfaces [717]. On the other hand, while HAp-coated implants inserted into cortical bone have been reported to achieve their maximum bone apposition 4 weeks post-implantation, uncoated surfaces were found to increase their bone apposition ratios until 12 weeks [718]. Hacking et al. [719] also argued that 12-week implantation has a clinical value. Therefore, in order to allow for complete osseointegration of both coated and uncoated implants, a comparative *in vivo* study should be carried out for at least 12-week implantation. Second, histomorphometric measurements on SEM- backscattered electron (BSE) images provide high contrast for differentiating between the various components. Thus, they have been claimed to provide more accurate quantitative analysis compared to either light microscope images of stained samples or micro-radiographic images [92,720,721].

Quantitatively, the osseointegration is usually expressed in terms of the bone apposition ratio (BAR; also known as the apposition index, AI) [92,189,720,722,723,724,725] and the new bone area (NBA) [726,727,728]. The BAR represents the length of the direct implant surface/bone contact divided by the length of the outer circumference of the implant. The NBA, on the other hand, represents the percentage of new bone within a distance of either 0.5 or 1 mm from the implant surface at the diaphysis and metaphysis, respectively. In order to calculate it, rings are created, with the implant perimeter being the inner surface of the ring. The sum of pixels that represent new bone is calculated and divided by the total number of pixels inside the ring. NBA measurements are less common in the literature than BAR measurements, possibly because the former might be more complicated to do accurately [92].

Two of the major factors that affect the osseointegration and the interfacial strength between an implant and bone are the surface chemistry and roughness. But which of the two factors is more important with respect to HAp coatings? Hacking et al. [724] referred to this question, using a canine femoral intramedullary implant model. Grit-blasted CP-Ti implants were compared to HAp-coated implants, as well as to implants first coated with HAp and then recoated with a very thin titanium film that preserved the topography of the HAp coating but masked the chemistry of HAp. Twelve weeks after implantation, the BAR averaged 23% for the GB-CP-Ti implants, 73.6% for the HAp-coated implants, and 59.1% for the HAp-Ti-coated implants. The interfacial strength of the recoated implants was about 80% of that achieved when HAp was exposed. Thus, the investigators argued that surface roughness was a larger contributor to interface strength than was the presence of the HAp chemistry. However, it should be borne in mind that while surface roughness is undoubtedly a factor in determining the interfacial strength when an implant is in intimate contact with bone, implants are often surrounded by gaps, so that micromotion occurs after implantation. Under these circumstances, the osteoconductive nature of the HAp coating is probably a key factor in promoting the intimate bone apposition required for the achievement of interface stability.

**Figure 8:**
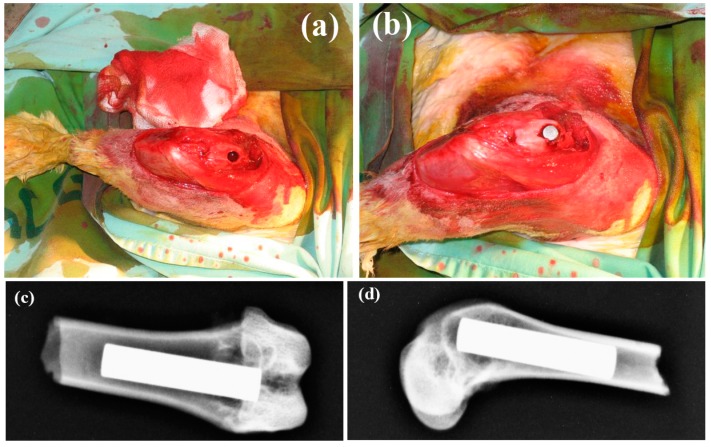
A widened hole in the medullary canal of the distal femur of a rabbit before (**a**) and after (**b**) press fitting of the implant; (**c**,**d**) Radiographs of the right distal femur of a rabbit (**c**—Anterior-Posterior, AP, **d**—Lateral, LAT). The implant is press fitted into the medullar canal, within both the metaphysis and diaphysis [92]. Reproduced with permission from Elsevier Ltd.

One of the important parameters in animal studies is the implant model. The non-weight-bearing intramedullary implant model better simulates the clinical implant site than the weight-bearing transcortical model [92,720,726]. Yet, the transcortical model is more common in the literature, simply because it is simpler for the surgeon. To illustrate this, Figure 8a shows the reamed intramedullary canal of a mature New Zealand white rabbit, while Figure 8b shows the press-fitted coated Ti–6Al–4V rod. The distal portion of the implant is within the metaphysis, while the proximal portion is within the diaphysis (Figure 8c).

HAp-related bone formation is believed to begin with surface dissolution of the HAp, which releases calcium and phosphate ions into the space around the implant. Reprecipitation of carbonated apatite then occurs on the coating surface [729]. The HAp binds serum proteins and cellular integrin receptors, allowing osteoblastic cells to bind to the surface [205,730]. Bone formation follows at both the bone and the coating surfaces [389]. Bone ongrowth develops more rapidly on coatings with low crystallinity because the initial dissolution and release of calcium ions is faster than those associated with coatings of high crystallinity [205,731]. It has also been shown that rough surfaces exhibit stronger interfaces with bone than do smooth surfaces, in both humans and animals, as long as the interface is bone ongrowth [732,733].

**Figure 9:**
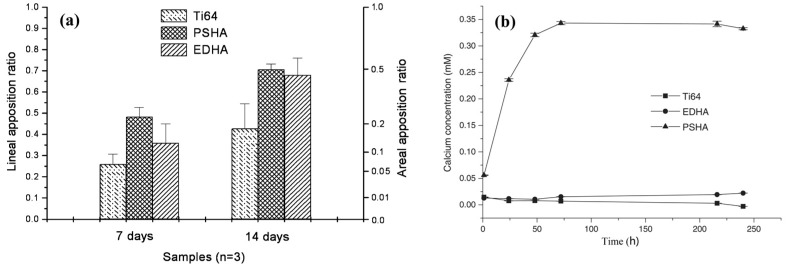
(**a**) Average bone apposition ratio (BAR) after 7 and 14 days of implantation. The error bars are standard deviations (*n* = 3); (**b**) Aqueous solubilities of plasma-sprayed hydroxyapatite (PSHA) and electrochemically deposited hydroxyapatite (EDHA) in deionized water at room temperature. Bare Ti–6Al–4V alloy serves as a reference (*n* = 1; error bars are standard deviations from three measurements) [189]. Reproduced with permission from Elsevier Ltd.

Finally, with respect to the effect of solubility, Lakstein et al. [92] related the enhanced osseointegration of Ti–6Al–4V implant electrodeposited with CaP after soaking in NaOH to the higher content of the OCP phase in this coating and the associated increase in the solubility of this coating *in vivo*. Wang et al. [189] implanted three different implants: bare Ti–6Al–4V alloy, Ti–6Al–4V alloy coated with plasma-sprayed hydroxyapatite (PSHA), and Ti–6Al–4V alloy coated with electrochemically deposited hydroxyapatite (EDHA), into canine trabecular bone for 6 h, 7 days, and 14 days, respectively. Figure 9a shows the average BAR for each type of implant after 7 and 14 days. EDHA shows a low BAR after 7 days, intermediate between the corresponding values for bare Ti–6Al–4V and PSHA implants. However, after 14 days, the EDHA bone apposition ratio increases markedly, to that observed for PSHA, and much more than that for bare Ti–6Al–4V. It was hypothesized that the initial low BAR may be attributable to the low solubility of EDHA *in vivo*. On the other hand, PSHA, with its partial amorphous content and consequently higher solubility *in vivo*, contributes a much higher local concentration of calcium and phosphorus ions, which could assist in and accelerate local mineralization of new bone or be involved in cell signalling. Nevertheless, the differing solubilities dictate only different short-term mineralization behaviours. By 14 days, the BAR of EDHA increases sharply and catches up with that of PSHA, suggesting that the lower dissolution rate of EDHA is already sufficient to catalyse the formation of new bone. In order to support the hypothesis of the solubility effect, the solubilities of the three types of implants were assessed *in vitro* by immersing then in water at room temperature for up to 240 h. The calcium concentration was then measured by a DCP-AES. Figure 9b shows the results of these solubility tests. It is evident that PSHA dissolves much more readily than EDHA. While the former reaches saturation in distilled water in 2 days, the latter occasions a very low Ca concentration even after 10 days.

## 14. CaP Coating Technologies

In the previous Sections, many references for CaP coatings were given. These could be monolithic, composite, or FGM. A key factor for successful fixation of cementless implants used for joint reconstruction is the establishment of a stable interface between the implant and bone. Coating of the implant with osteoconductive HAp is a well-known method for achieving such fixation [55]. The coating can improve the biocompatibility of orthopedic and dental implants by blocking the diffusion of poisonous elements from the metal into the body, as well as reducing the friction coefficient between the implant and its biological surroundings [734]. HAp is capable of enhancing bone growth across a gap around an implant in both stable and unstable mechanical conditions, and even converting a motion-induced fibrous membrane into a bony anchorage [735,736].

Synthetic CaP coatings can be prepared by a variety of processes [49,737,738], such as plasma spraying (PS) [49,389,739,740,741,742], high-velocity oxygen-fuel (HVOF) thermal spraying [743], sputter coating [744], radio-frequency magnetron sputtering [745], pulsed laser deposition (PLD) [746,747], ion-beam deposition [748,749], frit enamelling [49], hot isostatic pressing (HIP) [750], metallo-organic chemical vapour deposition (CVD) [751], derivation from sol-gels [752,753], electrophoretic deposition (EPD) [754,755,756,757], chemical deposition [758,759], and electrodeposition (ED) [92,184,185,189,191,238,425,618,760,761,762,763,764,765,766]. Some of these processes will be described below.

Thermal spray is defined as a process in which the coatings are applied by means of special systems through which melted or molten spray material is propelled at high speed onto a cleaned and prepared component surface. PS is a subgroup of thermal spray techniques. In this process, a high-frequency arc is ignited between an anode and a tungsten cathode. The gas flowing through between the electrodes (namely, He, H_2_, N_2_ or mixtures) is ionized so that a plasma plume develops. The temperature within the plume can reach as high as ~16,000 °C. The spray material is injected as a powder outside of the gun nozzle into the plasma plume, where it is melted and hurled by the gas onto the substrate surface. The molten and semi-molten particles rapidly solidify on impact against the substrate, and an integral layer is built-up by the overlaying of many particles. For specialized applications, a variant of the process is to plasma spray in a controlled, low pressure atmosphere. In contrast to coating in air (atmospheric plasma spraying, or APS), the melted particles oxidize far less with vacuum plasma spraying (VPS), resulting in coatings of considerably higher quality. When HAp is deposited onto Ti–6Al–4V, a chemical reaction with titanium dioxide at 1000 °C forms CaTiO_3_ and TCP. The CaTiO_3_ acts as a bond layer, thus obtaining a relatively high bond strength (as high as ~60 MPa). Some droplets may not completely melt before impacting the surface due to particle size, velocity and temperature distributions within the stream of particles and this results in embedded particles in the lamellar structure. HAp plasma sprayed under ambient conditions partially transforms to TCP and calcium oxide. This transformation can be avoided by keeping the plasma gas at a low hydrogen/argon ratio during the PS process. PS is characterized by high deposition rates, coating thicknesses most often in the range of 30–200 μm, and low cost.

Today, PS is still the most common technology used commercially for coating implants with CaP. However, PS suffers from several drawbacks. First, while β-TCP is formed at 1200 °C, phase transformation into TTCP occurs at *T* > 1400 °C. Therefore, PS coatings typically consist of several phases. Second, the thermal expansion coefficients of HAp and TCP (11–15 × 10^−6^ cm/(cm·K)) are larger than those of titanium-based alloys (8–10 × 10^−6^ cm/(cm·K)). Consequently, it is not easy to obtain good CaP coatings on metals by processes that involve high temperatures [767]. On cooling a HAp film that was deposited at high temperature, the crystalline HAp is constrained and is not allowed to shrink. As a result, the cooled film is under tensile stress. Conventional PS HAp films suffer from poor adhesion because these tensile stresses within the HAp film have a greater tendency to initiate cracks and cause film delamination. Even HAp films prepared by room temperature deposition or with post-deposition annealing may possess a combination of residual stresses from lattice misfit, coefficient of thermal expansion, and defect sources. However, these stresses seem not to play a significant role in reducing the coating’s adhesion to the substrate. Third, control of crystallinity, phase composition and surface morphology are hard to control. Fourth, PS is a line-of-sight technique. Long-term clinical experience has uncovered several shortcomings [49]. First, poor coating-substrate adherence might cause delamination after implantation due to high residual stresses in the coating and low resistance to shear and tensile stresses. Second, the phase composition produced causes the coating to release HAp particles into the intermediate space, causing an inflammatory and osteolysis process in the surrounding tissue, and accelerating wear processes of the acetabular component [49].

Some commercially available implants have solution-deposited biomimetic coating, in which the coating is nucleated and grown on the prosthesis in solutions [768,769,770,771]. In this case, the substrate is immersed in SBF solution (Table 7) under near-physiological conditions. A biomimetic coating is then nucleated and grows as bone-like crystals. Coatings of different structures can be generated under different ionic conditions and temperatures [49]. The biomimetic coating process is simple to preform, cost-effective, and can be applied even to heat-sensitive, non-conductive and porous materials of large dimensions and with complex surface geometries. Another advantage of this method is the ability to incorporate biologically active molecules that can be co-precipitated with the inorganic components. However, the coating process takes days, and the structure of the coating might also be influenced by the time of coating.

Another possible process for depositing CaPs is EPD. This is a process in which charged particles in a dispersion are migrated under electrical field toward the substrate electrode [772]. The method uses low-cost equipment, is easy to set up, is able to coat substrates with complex geometries, has high deposition rates. In addition, a high degree of control on the coating is enabled by regulating the deposition conditions, as well as the ceramic powder size and shape [773]. EPD is a cheaper method than CVD, sol-gel deposition and sputtering for producing films with a wide range of thicknesses, from less than 1 μm to more than 100 μm thick [774]. The growing interest in EPD stems from a variety of reasons, such as the high possibility for stoichiometric deposition, high degree of purity of material, which is not easy to achieve by the previous mentioned methods, and the fact that the deposition uniformity does not depend on the geometry of the substrate. However, limitations of this technique include low adhesion strength, low density, and cracking of the coating due to post-deposition sintering [754]. EPD of HAp requires a very stable suspension in order to obtain decent coating [775]. It can be conducted at room temperature, or even lower, which avoids problems related to formation of amorphous phases. The nature of the bond is more metallurgical than mechanical, thus HAp coatings by EPD are expected to have improved adhesion strength as compared to thermal sprayed techniques. However, a major drawback is the presence of porosity, which might later on lead to corrosion and delamination of the coating due to penetration of body fluids into the interface between the coating and the substrate. Post-deposition high-temperature sintering can be utilized to minimize the porosity and increase the coating density. Unfortunately, cracks in the coating can form during high-temperature sintering due to the difference in the thermal expansion coefficients and large reduction of the pore volume between the titanium and HAp [776]. In addition, since this process involves the application of high voltage to the substrate, the HAp NPs must be calcined before deposition in order to evaporate adsorbed water.

Ion-beam assisted deposition (IBAD) is a vacuum deposition process based on the combination of ion-beam bombardment and physical vapour deposition (PVD). The substrate and target are placed in a vacuum chamber where the coating is then deposited in a cold plasma atmosphere. There are various ion-bean techniques, including ion implantation, ion sputtering, ion planting, and ion-beam dynamic mixing, all of which produce thin, homogeneous, adherent CaP coatings. Many parameters can influence the composition, mechanical, chemical, and structural properties of the coating formed by the IBAD process. The most important parameters in IBAD are the evaporation rate or sputtering rate, coating material, ion species, ion-beam current density, and ions energy [777]. IBAD has the ability to prepare bio-coatings with considerably higher adhesion strength as compared to traditional coating methods. The high adhesion strength is the result of interaction between the substrate and coating atoms, assisted by ion bombardment. This results in an atomic intermixed zone at the substrate/coating interface [777]. Ohtsuka et al. [778] first used 50 keV $Ca^{+}$ implantation into Ti, followed by $Ca^{+}$ IBAD to deposit HAp on Ti substrate, forming coating with strong adhesion. Cui et al. [779] used $Ar^{+}$ IBAD to form highly adhesive HAp coating on titanium alloy. Generally speaking, IBAD is a promising technique for creating thin, defect-free CaP coatings [49].

In PLD, also known as laser ablation, the intense radiation of the laser is used to vaporize and deposit thin films [49]. The high power laser provides the energy source to melt and vaporize materials from a target. Owing to the high power density of a focalized pulsed laser, the ablated material forms a plasma plume consisting of a collection of highly excited molecules, atoms, ions, and electrons, which expand in a vacuum or a gas environment, transporting the material that condenses on a substrate. Pulsed lasers with high repetition rates allow growth of a thin film of the desired material. Preparing HAp thin films by PLD allow accurate control of HAp growth parameters at low deposition temperatures and the ability to produce highly crystalline HAp coatings [780,781]. Controlled thickness and homogeneous composition are easily achieved [49]. The lower thickness of the coatings produced by this method also lowers the risk of delamination. In addition, PLD does not require pre-treatment of the implant’s surface before deposition. However, the morphology of the coating follows that of the substrate, thus requiring a texturing step before coating [49]. The adhesion strength of HAp coating on metals depends on the microstructure of the substrate, the surface chemistry, and the PLD process parameters, such as laser power density and substrate temperature [697,782,783]. The main limitation of PLD is the splashing of the nanoparticulates on the film [784]. Furthermore, this is a line-of-sight technique, and the coating thickness is usually 0.05–5 μm.

Sol-gel coating is one of the simplest techniques to manufacture thin films, which can produce almost any single- or multi-component oxide layer on glass or metals [785,786]. Sol-gel coating can produce various materials with special functions, such as electronic, magnetic or special chemical functions [787]. The sol-gel process involves the formation of solid materials, mainly inorganic non-metallic, from solution. This can be a solution of monomeric, oligomeric, polymeric or colloidal precursors [788]. The sol-gel process consists of five stages [788,789]: (1) Producing homogenous solution of the sol-gel precursors; (2) Forming sol by adding suitable regent, e.g., water for oxide ceramics; (3) Changing the sol to gel by poly-condensation; (4) Shaping the gel according the final preferred shape, e.g., thin film, fibre, etc.; (5) Sintering the shaped gel to obtain the desired ceramic material. The sol-gel process has several advantages, such as excellent adhesion, good corrosion resistance, high purity material production, low sintering temperatures (200–600 °C), process simplicity, and ability to coat complex shapes [790,791]. However, the sol-gel technique has disadvantages, such as high permeability, low wear-resistance, difficult porosity control, and expensive raw materials, which have limited its utilization in industry [790]. The coating thickness is typically less than 1 μm. The sol-gel is a process which can be carried out at room temperature, and therefore, structural complication of HAp instability at high temperatures is avoided [392]. Gross et al. [792] noted that the production of sol-gel HAp coatings on titanium using alkoxides precursors requires strict control of the firing and aging temperatures due to phase transformation of the substrate. Moreover, HAp deposition by sol-gel process requires extremely stringent processing parameters, such as duration and calcining temperature, chemical composition of the precursors, and type of substrate, which significantly influence on the crystalline phase of the HAp, the adhesion strength, and the biocompatibility of the coated substrate [791,793,794].

Since the early 1990s, much interest in ED of CaP [795,796,797,798,799,800,801,802,803,804] has evolved due to: (1) The low temperatures involved, which enable the formation of highly crystalline deposits with low solubility in body fluids and low residual stresses; (2) The ability to coat porous, geometrically complex or non-line-of-sight surfaces; (3) The ability to control the thickness, composition, and microstructure of the deposit; (4) The possible improvement of the substrate/coating bond strength; (5) The availability and low cost of equipment; (6) The ability to incorporate biological matter in the coating during its processing. ED of CaP from ionic solution is usually done in metastable solutions supersaturated with respect to one or more CaP phases. The deposition is occurring on the surface either galvanostatically or potentiostatically by reduction of water on the cathode, where the substrate is placed, and deprotonation of phosphoric acid in solution. This enables the pH to rise in vicinity to the cathode, which promotes precipitation on the substrate of the phases thermodynamically favourable for precipitation from solution. Remaining issues with ED CaP coatings are reproducibility of results in scale-up, low deposition rate (as much as 3 h may be required to form sufficiently thick coating), and some adhesion problems depending on surface preparation.

The deprotonation of dihydrogen phosphate $H_{2}PO_{4}^{-}$ to $HPO_{4}^{2-}$ serves as an important biological buffer system, which operates in the internal fluid of all cells, stabilizing the pH at around 7.21 (in mammals, pH = 6.9–7.4). Thus, the speciation curves of phosphoric acid may aid, at least to some extent, in understanding the effect of pH on the electrochemical deposition of CaPs [184]. At 37 °C, the dissociation constants of phosphoric acid are [184]:

(5) $$H_{3}PO_{4}\to H_{2}PO_{4}^{-}+H^{+}\;pK_{1}=2.207$$

(6) $$H_{2}PO_{4}^{-}\to HPO_{4}^{2-}+H^{+}\;pK_{2}=7.182$$

(7) $$HPO_{4}^{2-}\to PO_{4}^{3-}+H^{+}\;pK_{3}=12.023$$

**Figure 10:**
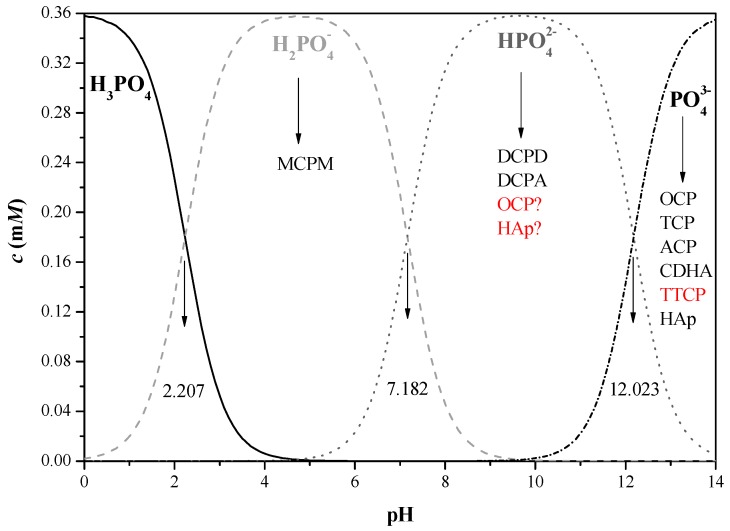
The distribution of phosphate species as a function of pH at 37 °C, 0.36 mM total analytical concentration of phosphate, free hydrogen concentration of 10^−6^ M. The values of the three dissociation constants are marked, along with the CaP phases that are likely to form from each species. As the pH increases, the Ca/P ratio in the solid phase increases, and the solubility of this phase decreases [49], both *in vitro* and *in vivo*.

With the aid of a chemical equilibrium code, for example, the speciation curves shown in Figure 10 can be constructed. This figure shows, for example, that when depositing from an electrolyte solution at pH = 6.0, both $H_{2}PO_{4}^{-}$ (majority) and $HPO_{4}^{2-}$ exist in the solution [765], hence both can participate in the precipitation of a CaP coating. When increasing the pH to 7.4, $HPO_{4}^{2-}$ becomes the major species in solution. In order to allow reactions that consume $PO_{4}^{3-}$, the local pH must first increase significantly [185,765]. In Figure 10, TTCP is marked in red under $PO_{4}^{3-}$ because it cannot be precipitated from aqueous solutions (see Section 6.9). OCP and HAp are marked in red with a question mark under $HPO_{4}^{2-}$ because many publications wrongly use the term orthophosphate with respect to formation of HAp and other CaPs, whereas this term specifically refers to $PO_{4}^{3-}$. Furthermore, from Section 6 one may mistakenly conclude that OCP, TCP, ACP, CDHA and HAp are all associated with the “third acidity of phosphoric acid” (namely, with $PO_{4}^{3-}$). However, from Equations (8) and (10)–(12) it is evident that both OCP and HAp can form in chemical reactions that involve either $PO_{4}^{3-}$ or $HPO_{4}^{2-}$ as a reactant. Finally, the columns of phases in Figure 10 are arranged in a descending order of solubility (see Section 6). Note that only orthophosphate salts are usually considered because metaphosphates and pyrophosphates hydrolyse in body fluids. It has been suggested that ED of CaPs is much different than that of metals and alloys. The former may have several routes, such as acid-base reactions that are facilitated by the local pH increase (as a result of cathodic polarization), precipitation in solution following the decrease in the solubility of the apatite phase in accordance with the solubility isotherms [176] as the local pH is increased (as a result of cathodic polarization), or precipitation in solution due to direct production of sufficient phosphate ions by electrochemical reactions at sufficiently cathodic potentials. Based on calculations of the limiting current densities of different possible cathodic reactions [765] and calculation of a remarkably high number of electrons transferred in the reaction [185] it was concluded that electrolysis of water plays a key role in the consumption of electrons and rapid increase of the local pH. Some of the possible chemical and electrochemical reactions leading to formation of CaPs in solution are listed below:

(8) $$5Ca^{2+}+3PO_{4}^{3-}+{\left(OH\right)}^{-}\to \left[HAp\right]$$

(9) $$5CaHPO_{4}+H_{2}O\to \left[HAp\right]+2H_{3}PO_{4}$$

(10) $$5Ca^{2+}+3HPO_{4}^{2-}+H_{2}O\to \left[HAp\right]+4H^{+}$$

(11) $$8Ca^{2+}+6PO_{4}^{3-}+2H^{+}+5H_{2}O\to 2\left[OCP\right]$$

(12) $$8Ca^{2+}+6HPO_{4}^{2-}+5H_{2}O\to 2\left[OCP\right]+4H^{+}$$

(13) $$H_{2}PO_{4}^{-}+{\left(OH\right)}_{ads}^{-}\to PO_{4}^{3-}+H_{2}O$$

(14) $$2H_{2}PO_{4}^{-}+2e^{-}\to 2HPO_{4}^{2-}+H_{2}$$

(15) $$H_{2}PO_{4}^{-}+2e^{-}\to PO_{4}^{3-}+H_{2}$$

**Figure 11:**
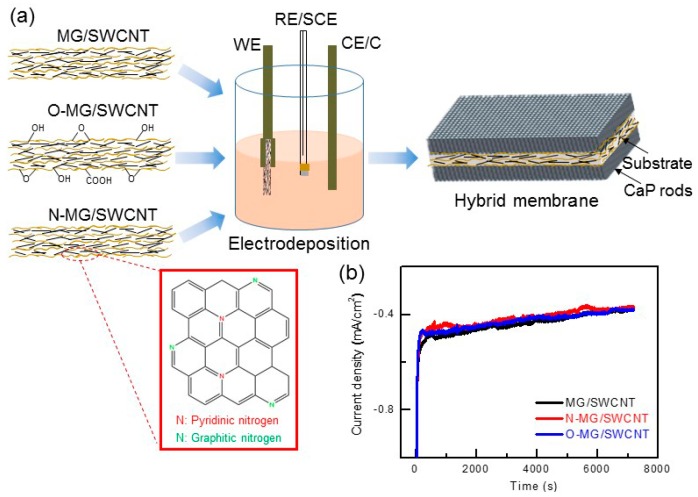
(**a**) Schematic diagram for the synthesis of CaP coating on flexible carbon-based membranes by electrodeposition; (**b**) Current transients of three types of membranes during the deposition process [805]. Reproduced with permission from Wiley-VCH Verlag GmbH and Co. KGaA, Weinheim.

Zhang et al. [805] synthesized well-aligned biomimetic hexagonal needles of HAp on flexible, freestanding mesoporous graphene (MG)/SWCNT hybrid membranes by a simple, low-cost, and environment friendly electrochemical process. The chemical composition and surface morphology of the HAp coating resembles those of biological apatite. Nitrogen doping and oxygen plasma etching of the MG/SWCNT membranes increased the density of nucleation sites and yielded more uniform coatings with smaller HAp needles. The coated membranes showed excellent biocompatibility and bioactivity *in vitro* based on proliferation test of human fetal osteoblast (hFOB) osteoprogenitor human cells. They also showed significantly better biomineralization *in vitro*. Nitrogen-doped hybrid membranes gave the best results. It was claimed that the hybrid membranes hold great promise in biomedical applications such as patches and strips for spine fusion, bone repair, and restoration of tooth enamel. However, for this to happen, concerns related to the implantation of nano-carbons should first be resolved. Figure 11a shows the schematic diagram of the ED of the hybrid membranes. The MG/SWCNT hybrid substrate was prepared using a soft-templated strategy with subsequent vacuum filtration method. Nitrogen doping of the membranes was conducted in urea solution by hydrothermal method. Oxygen plasma was performed to introduce hydrophilic oxygen-containing functional groups on the surface of MG/SWCNT. The typical deposition current density curves at constant potential of −1.4 V are shown in Figure 11b. It can be seen that in all three cases the initial cathodic charging current decayed rapidly and attained a steady-state current within a few minutes. Moreover, during deposition the current density dropped slightly due to the increasing electrical resistance of the hybrid membranes. Yet, no significant difference was observed between the current transients of the three membranes. This suggests that the kinetics of deposition was not affected by the doping of the membrane and that the deposition mechanism and the electrochemical reactions involved may be similar.

There are many reasons for pre-treating a metallic implant before coating with CaP; for example, to increase the adhesion strength, control the corrosion rate, increase nucleation kinetics, control the structure and porosity of the coating, improve the bioactivity and osseointegration of the coating, etc. Therefore, different pre-treatment practices have been implemented for CaP coatings. As discussed earlier, the adhesion of the CaP coating to the implant’s surface is a very important parameter, which affects and performance and longevity of the implant. Adhesion failures result in delamination of the coating and loosening of the implant, thus leading to mechanical failure and bone resorption. The US FDA as well as ASTM and ISO standards require an adhesion strength of at least 15 MPa [806,807,808,809,810,811,812,813,814]. In order to achieve this value, pre-treatment of the metal surface is sometimes required.

**Figure 12:**
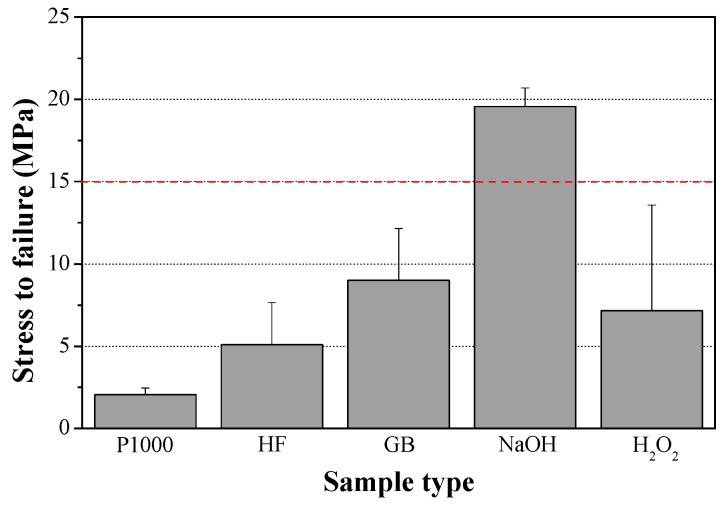
The tensile stress to failure of ED HAp-coated samples with different pre-treatments during adhesion test. The data is presented in terms of mean + standard error of the mean (*n* = 3). The red dash line defines the minimum adhesion strength required by the US FDA for coating adhesion strength in orthopedic and dental endosseous implants [237]. Reproduced with permission from Springer Science+Business Media, LLC.

Eliaz et al. [237] studied the effect of various combinations of mechanical and chemical surface treatments on the adhesion strength of electrochemically deposited HAp coating on CP-Ti. These surface pre-treatments included: (1) mechanical grinding down to P1000; (2) grinding to P1000 followed by chemical etching in HNO_3_/HF solution; (3) grinding to P1000 followed by etching in HNO_3_/HF solution and then grit blast; (4) grinding to P1000 followed by etching in HNO_3_/HF solution, grit blast and soaking in a stirred solution of 5 M NaOH at 60 °C for approximately 24 h, followed by heat treatment for 1 h at 600 °C and overnight cooling in the furnace; (5) grinding to P1000 followed by etching in HNO_3_/HF solution, grit blast and soaking in a 5 M H_2_O_2_ solution, followed by heat treatment for 24 h at 60 °C. The tensile stress to failure of each type of pre-treated samples is shown in Figure 12. It is evident that pre-treatments series #4 yields the highest adhesion strength to the HAp coating, and the only one that satisfies the FDA requirement. The stress to failure increased as the surface roughness of the substrate was increased by pre-treatments. This finding is supported by the work of de Senna et al. [815], who evaluated the effect of different surface finishes on the adhesion of EPD HAp on Ti. Piveteasu et al. [816] found that an increase of the substrate roughness improves the adhesion of a sol-gel made CaP coating to titanium. It was also found that a smaller coating thickness is associated with higher adhesion strength.

In another article, Eliaz et al. [238] demonstrated the effects of both surface preparation and surface post-treatment by exposure to electron beam on the surface morphology, contact angle and the interaction with bone-forming cells of ED HAp coating on CP Ti. One of the pre-treatments, in Ref. [238] too, was soaking in NaOH. Kokubo et al. [817,818] reported that soaking in NaOH followed by heat treatment significantly increases the bond strength of bone to uncoated titanium rods in rabbit femora. Lakstein et al. [92] reported enhanced osseointegration of ED HAp on Ti–6Al–4V alloy due to a pre-treatment consisted of soaking in NaOH without subsequent heat treatment. In addition, that coating exhibited reduced occurrence of delamination compared to the commercial PS HAp coating. It was suggested [796,817,819,820,821,822] that after soaking in the alkaline NaOH solution, a hydrated titanium oxide gel layer containing Na^+^ ions is formed on the surface. A complementary heat treatment then dehydrates and densifies this layer, transforming it to amorphous sodium titanate (NaHTiO_3_) with a porous network structure. This phase is a precursor for amorphous calcium titanate, which induces nucleation of amorphous calcium phosphate, and then HAp. It was reported [796] that NaOH treatment prior to ED in a modified SBF resulted in both a denser and a more uniform DCPD/HAp coating. It was speculated that the porous network of the titanium surface formed after the NaOH pre-treatment provided more favourable sites for the nucleation of CaP. In regard to soaking in hydrogen peroxide (H_2_O_2_), it was suggested [795,823,824] that this pre-treatment of titanium substrates results in formation of a relatively thick porous oxide layer on the titanium surface. In an aqueous medium, $OH^{-}$ bonds to the Ti cation in TiO_2_, forming Ti–OH groups, which may be either acidic or basic, depending on the pH of the electrolyte. Application of cathodic potential results in a relatively high concentration of $OH^{-}$ ions in the vicinity of the cathode surface, thus locally increasing the pH and providing better conditions for the nucleation and growth of HAp.

**Figure 13:**
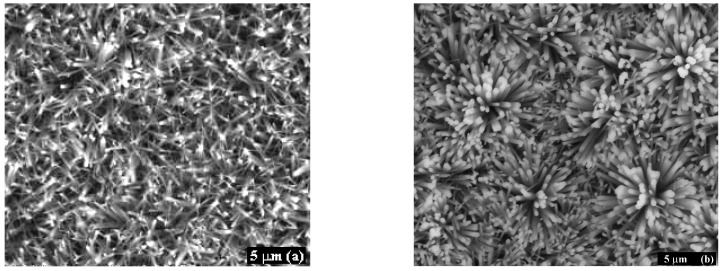

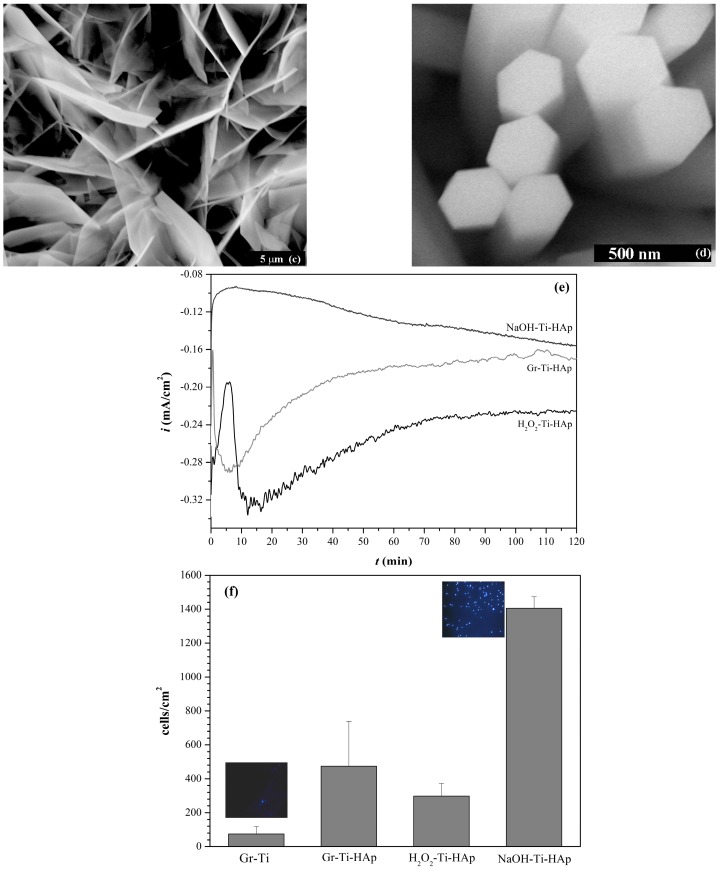
(**a**–**c**) SEM images revealing the typical surface morphologies of electrochemically deposited hydroxyapatite (ED Hap) on ground Ti, Ti soaked in NaOH, and Ti soaked in H_2_O_2_, respectively; (**d**) High-magnification image of (**b**), which reveals the hexagonal cross-section of the bars; (**e**) The typical current density transients during potentiostatic deposition of HAp on the three types of substrate; (**f**) Cell density on different surfaces (partial population). The data are presented as mean ± standard deviation. Inset: Two typical fluorescent images of cell nuclei (Hoechst staining) on Gr-Ti vs. NaOH-Ti-HAp [238]. Reproduced with permission from Elsevier Ltd.

Surface pre-treatments can also affect the surface morphology and other physical properties of the CaP coating. Figure 13a–d [238] reveals that ED HAp coatings on either mechanically ground CP Ti substrate or substrate soaked in NaOH grinding before ED and soaking in NaOH before Gr-Ti-HAp and NaOH-Ti-HAp consist of needles (or whiskers). In the case of NaOH pre-treatment (Figure 13b), these needles are arranged in aggregates with a more distinct preferred orientation. SEM image at higher magnification (Figure 13d) reveals that the needles are actually prismatic hexagonal bars, approximately 300 nm in diameter. Often, the outer shape of a crystal, as observed by electron microscopy, is related to the point group symmetry to which the crystal belongs. Thus, it is likely that each bar in Figure 13d is a single crystal of HAp. The HAp coating on CP Ti pre-treated in H_2_O_2_ exhibits a platelet morphology (Figure 13c), but the size of each platelet is much larger and the visual porosity level seems to be significantly higher. Figure 13e shows that different surface pre-treatments result in different current density transients during potentiostatic deposition of HAp, which can be associated with the different HAp crystal shapes and sizes. Each treated substrate was also associated with different surface roughness and wettability. Figure 13f shows the digital cell (mouse osteogenic cell line MBA-15) counts per area unit (partial population). Two typical fluorescence images of the cell nuclei (Hoechst staining) are also included. The highest number of cells is counted on HAp coating electrodeposited on NaOH-soaked titanium, while the lowest number is counted on HAp electrodeposited on ground Ti.

Tanahashi and Matsuda [825] studied the effect of self-assembled monolayers (SAMs) on the growth of biomimetic CaP coatings. It was found that while some chemical head groups, such as phosphate and carboxylic groups, increase the nucleation process, others (e.g., methyl group) inhibit it. Metoki et al. [766,826] studied the effect of chemical surface modification of titanium alloy substrates by SAMs on ED CaP coatings. It was discovered that not only is the nucleation and growth of the coating different, but also the morphology of the coating is substantially altered. Waterman et al. [771] studied the effect of Mg(OH)_2_ pre-treatment for different time periods and additives on the corrosion rate of CaP-coated Mg alloy. It was discovered that the oxide layer formed during pre-treatment affects the amount of defects present in the coating that follows. Longer pre-treatment times induced cracks and defects in the coating layer, which led to higher corrosion rates. On the other hand, addition of calcium and phosphate ions to the solution promoted additional nucleation of CaP and led to increased corrosion resistance.

Post-treatments after coating are also applied in many cases, for example to convert CaP phases with lower Ca/P ratio to HAp. While there are many methods for preparation of CaP coatings, there are only two post-treatments that are used commercially: sintering [754,757,776,827,828] and soaking in an alkaline solution [829,830,831]. These post-treatments often take several hours. Heat treatment, usually at 600–800 °C for 1 h, transforms the water trapped in the coating during deposition processes such as sputtering, ED, EPD and PS into ${\mathrm{OH}}^{-}$ ions, thus stabilizing the crystalline structure [49]. Shirdhar et al. [832] investigated the influence of those two post-treatments on Co-Cr-Mo alloy EPD with CaP coating. XRD analysis confirmed that the majority of the DCPD phases initially deposited on the surface transformed to crystalline HAp after either sintering or alkaline post-treatment. However, a higher percentage of crystallinity of HAp was observed in the case of sintering. The two also showed different surface morphologies: the sintering post-treatment yielded flake-like morphology which was claimed to be beneficial for the improvement of osseointegration, while the alkaline post-treatment yielded chrysanthemum-like structures. It was also shown that the corrosion resistance of the sintered coating was higher. Lee et al. [833] studied sintered PS coating. Sintering was found leading to recrystallization of ACP and its conversion into HAp. No significant difference was observed between the adhesion strength of treated and untreated coatings. Moreover, the coating showed better corrosion resistance after sintering. Caulier et al. [834] confirmed *in vivo* that heat-treated PS HAp implant had less reduction in coating thickness since the dissolution rate of the coating decreased.

While sintering is a good way to convert low CaP phases to crystalline HAp and minimize porosity by increasing the coating density, it is not applicable in some cases. For example, in magnesium alloys, sintering post-treatment is not possible due to the low melting point of the substrate [832]. As an alternative, alkaline post-treatment is often used. This usually takes a few hours and involves immersion in a strong base solution (NaOH) heated to 80 °C. For example, Su et al. [518] demonstrated the conversion from DCPD to HAp on different magnesium alloys using 1 M solution of NaOH at 80 °C for 2 h. The same process of alkali solution conversion was reported for other metals as well [801]. This conversion process is known to occur because of the high stability of the HAp in alkaline environment. Recently, Lin et al. [803] suggested a post-treatment comprised of lower concentration NaOH solution with an addition of Na_3_C_6_H_8_O_7_ at 85 °C for 5 h. HAp coating with hierarchical structure was reported. It was postulated that citrate plays a role in the preservation of shape and size of the original crystals during the phase transition.

Several studies have reported that using fluoride-modified alkaline solution may enhance the conversion of DCPD to HAp. For example, Su et al. [835] converted CaP deposited through phosphating method to HAp using different post-treatment solutions. NaF was inserted into the alkaline solution, and the effect of the pH and the pre-treatment time were evaluated. The best corrosion resistance was achieved after treatment at pH = 12 for 2 h. Suge et al. [836] investigated the effect of fluoride concentration during post-treatment on the phase content of the coating. It was found that the Ca/P ratio was higher in the presence of NaF, and that the CaP gradually changed from DCPD to HAp as the concentration of NaF was increased.

### US FDA and International Standards Requirements

CaP coatings on orthopedic and dental endosseous implants must satisfy a variety of requirements of the US FDA as well as ASTM and ISO standards [806,807,808,809,810,811,812,813,814]. These are summarized in Table 8. While the tolerance of chemical composition is fairly small, the level of crystallinity of PS coatings may vary within a fairly wide range [389], Table 8.

Several brief comments are given here on several experimental techniques commonly used. First, the XRD JCPDS files typically refer to powders and not to coatings and thin films. When comparing the measured *d* values to those in the standard files, it is not always possible to index the reflections based on a deviation smaller than ±0.01 Å. Shift in the measured reflections may be associated, for example, with the nonpowder character of electrodeposited samples, different processing techniques, distortions in the unit cell dimensions, etc. [184].

CaPs are complex structures capable of enclosing many substitutions and vacancies, which may be poorly crystallized. Therefore, their study by spectroscopic techniques might not provide full information on the fine structure, for example, the presence and location of functional groups such as ${\mathrm{CO}}_{3}^{2-}$, ${\mathrm{HPO}}_{4}^{2-}$, or ${\mathrm{OH}}^{-}$. Vibrational spectroscopies bring, in addition to structural identification, this valuable information. They may also supply information on the orientation of molecular species and crystals. Several techniques involve transition between vibrational levels. Yet, most commonly used are the FTIR and Raman spectroscopies [49,72,249]. Theoretically, there are four vibration modes (*ν*_1_–*ν*_4_) present for the ${\mathrm{PO}}_{4}^{3-}$ ion. The *ν*_4_ (asymmetric bending) bands at 520–660 cm^−1^, as well as the *ν*_1_ (symmetric stretching) and *ν*_3_ (asymmetric stretching) spectral bands positioned at 900–1200 cm^−1^, are useful in identifying the structure of apatites. The *ν*_2_ band of ${\mathrm{PO}}_{4}^{3-}$ is positioned at around 469–473 cm^−1^. A small absorption peak at ~525 cm^−1^ may be assigned to the bending mode of ${\mathrm{HPO}}_{4}^{2-}$, which is characteristic of crystalline acid phosphate, thus indicating the presence of OCP. A libration band at ~630 cm^−1^ is typical of structural ${\mathrm{OH}}^{-}$ in HAp. The O–H stretching vibration is unique for HAp, and its intensity is considerably weaker compared to the strong P–O stretching vibration because of the stoichiometry of HAp. Bands at 858 and 915 cm^−1^ may be attributed to the P–(OH) stretching mode of the acid orthophosphates groups. Bands in this region are characteristic of OCP and are useful in identifying its presence in mixtures with HAp. A band in the region ~950–980 cm^−1^ results from the *ν*_1_ stretching vibration of ${\mathrm{PO}}_{4}^{3-}$. The *ν*_3_ band of ${\mathrm{PO}}_{4}^{3-}$ has mostly been indexed in the range 1040–1125 cm^−1^. A peak at ~1192 cm^−1^ is also believed to arise from hydrogen-bonded OH between O_3_PO–H–OPO_3_ groups of ${\mathrm{HPO}}_{4}^{2-}$ ions. *ν*_3_-bands at 1370–1650 cm^−1^ are attributed to the presence of adsorbed (surface) ${\mathrm{CO}}_{3}^{2-}$. The origin of this carbonate could be the atmosphere (e.g., in the IR chamber). Peaks around 2350 cm^−1^ are indicative of absorbed ${\mathrm{CO}}_{3}^{2-}$. It is well-known that the carbonate ion may substitute for either the hydroxyl or the phosphate ions in HAp [184]. The CO_3_-for-PO_4_ substitution was shown to cause reduction in crystallinity and change in the shape of crystals from acicular to rod-shape, and then to equiaxed flat crystals [67]. A band at ~2362 cm^−1^ is characteristic of calcium-deficient apatite, and corresponds to an interphosphate oxygen bond. A broad band in the range 2800–3600 cm^−1^ is attributed to the stretching vibrations of hydrogen-bonded adsorbed water and hydroxyl ions. Structural ${\mathrm{OH}}^{-}$ stretching peak at ~3572 cm^−1^ is typical of HAp (and does not appear, for example, in the FTIR spectrum of OCP). On the other hand, an extended noise at ~3600–3900 cm^−1^ may indicate on a rather wet (i.e., not just hydrated) sample [184].

Informative description of coating adhesion tests and a MATLAB program developed for counting stained cells in light microscope images is given in [237]. Informative description of EQCM and XPS measurements is provided in [185]. EDS analysis is often performed to determine the atomic Ca/P ratio in an attempt to determine the phase content. However, the use of this technique for this purpose might lead to wrong conclusions [185]. For example, a comparative study has concluded that the Ca/P ratio obtained by SEM-EDS might involve a 7% deviation [837]. Based on our own experience, the deviation might be even larger.

The size and shape of CaP crystals and aggregates play a critical role in their applications. Reference [838] summarizes the state-of-the-art for the synthesis of CaP crystals with controlled sizes, from the nanoscale to the macroscale (up to centimetre size), and their diverse shapes, including the 0-D shapes of particles and spheres; the 1-D shapes of rods, fibres, wires, and whiskers; the 2-D shapes of sheets, disks, plates, belts, ribbons, and flakes; and the 3-D shapes of porous, hollow, and biomimetic structures similar to biological bone and tooth. In addition, possible directions of future research and development in this field, such as the detailed mechanisms behind the size and shape control in various strategies, the importance of theoretical simulation, self-assembly, biomineralization and sacrificial precursor strategies in the fabrication of biomimetic bone-like and enamel-like CaP materials are proposed. The outcome of several synthesis methods was also summarized. SEM and TEM were the key tools in assembling a large variety of images of crystals. For example, hexagonal fluoroapatite crystals were grown by a double-diffusion technique under controlled conditions (pH = 7.4, 37 °C, constant ion concentrations, 7 days) [77].

## 15. Clinical and Industrial Applications

One of the main applications of CaPs is for cementless hip implants [255,463]. Coating the stem’s surface with CaP can allow for bone-bone bonding and osteoconduction of the implant. This technique is sometimes called interface bioactive bone cement (IBBC) [72]. This approach is also applied on dental implants. Another wide use of CaP is as bone fillers of defects, used in reconstruction surgery. In this case, HAp is proved to be most efficient as it is not resorbable, binds to the bone physicochemically, and is mechanically strong enough [72]. CPCs have shown promise in bone replacement in oral surgery and craniofacial applications [226,256,839]. Another use of CPC is in osteonecrotic sites in the body that encourage bone growth [226]. CaP scaffolds, either porous or dense, are used for various applications, such as alveolar ridge augmentation, immediate tooth replacement, maxillofacial reconstruction, burr-hole buttons, etc. Other examples include orbital implants, increment of the hearing ossicles, spine fusion and repair of bone defects [242]. In order to permit growth of new bone into defects, a suitable bioresorbable material should fill these defects. Otherwise, ingrowth of fibrous tissue might prevent bone formation within the defects. Scaffolds can also appear as FGMs. Bone grafts are also proposed as non-hardening injectable CaPs and as pastes [840]. These generally consist of a mixture of CaP powder, particles or granules, and a highly viscous hydrogel [347]. Self-setting formulations of CaP pastes are used for minimally invasive surgery. Some are even able to form porous bioceramics. Other applications for CaP in dentistry also include toothpaste and chewing gum [252]. CaPs were found to promote a partial remineralization of a demineralized enamel, as well as have whitening effect and reduce tooth sensitivity [400]. CaPs are also used as carriers for nucleic acids (DNA or RNA) into nuclei of living cells for gene therapy [841]. Table 9 lists some trademarked CaPs. It should be noted that compounds with Ca/P < 1 are not suitable for implantation due to their high solubility and acidity.

**Figure 14:**
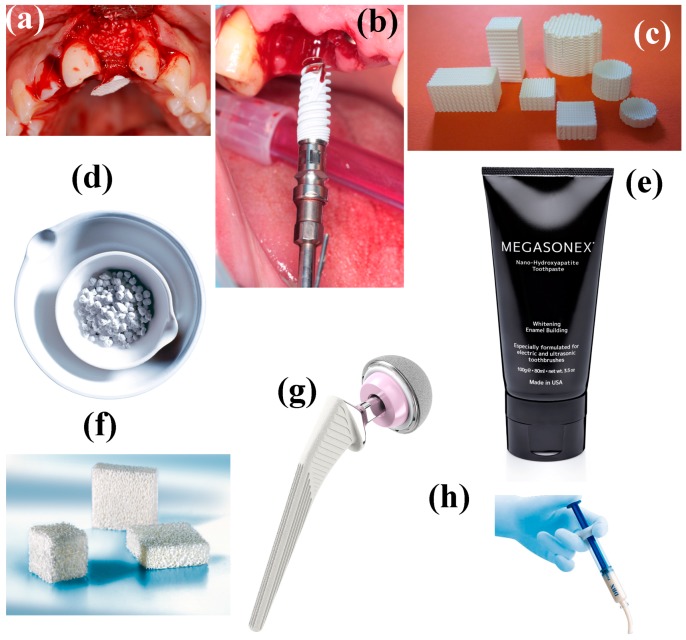
Various applications and forms of commercially available CaP-related products. (**a**) Bone augmentation after extraction of the left central incisro tooth. Courtesy Dr. Eyal Tarazi, DMD, Caesarea, Israel; (**b**) Coated dental implant. Reproduced with permission from SGS Dental Implant System [842]; (**c**) Augmentos^®^ 3D Scaffold bone substitute material [843] for filling or reconstructing non-load-bearing bone defects or for filling bone defects that are sufficiently stabilized by appropriate means. This seems to be the first 3D-printed CaP cement. The extrusion printing process does not involve any heat treatment steps. Reproduced with permission from InnoTERE GmbH; (**d**) Calcibon^®^ self-setting cement granules consisted of α-TCP, CaHPO_4_, CaCO_3_ and HAp [844]; (**e**) Megasonex^®^ Nano-Hydroxyapatite Toothpaste [845]. This is the world’s first nano-HAp toothpaste designed specifically for electric and ultrasonic toothbrushes. Nano-HAp helps to safely remineralize enamel (potentially reversing early stage tooth decay, white spot caries) and encrusts harmful bacteria (helping to prevent plaque formation). Other ingredients include tetrasodium pyrophosphate (prevents plaque from sticking), sorbitol, xylitol, mica, titanium oxide, citric acid, sodium carboxymethylcellulose, sodium saccharin, glycerin and silica. This toothpaste is free of fluoride and undesirable foaming agents such as sodium lauryl sulfate (SLS). Reproduced with permission from Goldspire Group, Ltd. The first toothpaste containing synthetic HAp as an alternative to fluoride for the remineralization and reparation of tooth enamel, BioRepair^®^ [846], appeared in Europe in 2006. The biomimetic zinc HAp (named microRepair^®^) is intended to protect the teeth by creating a new layer of synthetic enamel around the tooth instead of hardening the existing layer with fluoride that chemically changes it into fluorapatite; (**f**) Osteovit^®^ xenograft bone substitute [847]. Reproduced with permission © B. Braun Melsungen AG; (**g**) DePuy Synthes CORAIL^®^ cementless hip prosthesis for total hip arthroplasty [848]; (**h**) DePuy Synthes DBX™ Material bone graft substitute composed of demineralized bone matrix (DBM) from human donors in a sodium hyaluronate carrier [849].

CaPs are available in various physical forms: powders, particles, granules, dense blocks, porous scaffolds, injectable formulations, self-setting cements and concretes, implant coatings and composite component of different origin (natural, biological, or synthetic). Bone grafts are also proposed as non-hardening pastes (or “putty”). Usually, the latter materials consist of a mixture of CaP granules and a “glue”, typically a highly viscous hydrogel. Custom-designed shapes like wedges for tibial opening osteotomy, cones for spine and knee, and inserts for vertebral cage fusion, are also available. Figure 14 shows some of the current products on market.

## 16. The Future of Calcium Phosphates

Although CaPs have been used in the clinics for more than three decades, some issues that limit their use have not been resolved yet. Some of the major issues seem to be:

(1) *Dental community mistrust*. Interfacial failure of past dental implants coated with PS CaP has left a mark on CaP coatings. When first bursting into the dental industry as coatings, some PS CaP coatings failed within several months or years [850,851]. The cause has been attributed to the extensive dissolution of the coating as well as to its delamination [850,852]. These problems have since been eliminated; yet, many in the dental community have lost their faith in these coatings. A major campaign or sponsored Health Maintenance Organization (HMO) implants may reinstate CaP coatings to dental implants.
(2) *Short-term infection*. As described earlier, most infections occur due to bacteria adhering to the implant’s surface during implantation, causing a biofilm to form on it prior to implantation [587,853]. In general, infections can be classified based on their time of onset. Most of the infections develop from an early contamination that occurs during the operation or in the first few days after surgery. Events such as these, which become symptomatic or anyway manifest shortly following surgery, within three months of implantation, have been referred to as “early” infections [589]. These infections can be prevented by CaP incorporated with either a drug release system or anti-fouling agents. Anti-fouling or on-demand drug release systems, if designed well, can also be used to prevent long-term infections. This is a goal marked by many companies today.
(3) *Long recovery time*. CaPs are very good osteoconductive agents. Yet, the recovery period of implants integrated with CaPs is not immediate, unlike other techniques. For example, PMMA fixation of hip implants is immediate, and the patient may apply weight on the implantation site almost immediately after the operation. CaP coatings, and as such so are the scaffolds and cements, need time to allow good osseointegration, and thus extend the recovery period of patients. Encouraging faster integration, or somehow allowing for a bridge to such, may increase the use of CaP products.
(4) *Long-term issues with implants*. Both long-term infection and resorption of the surrounding bone (e.g., due to stress shielding) introduce serious problems. Sensors allowing the doctor, or even the patient, to monitor the environment of the implant’s surface may allow earlier intervention, especially if such intervention can activate dormant agents within the coating.
(5) *Mechanical strength of scaffolds/cements for tissue engineering*. Biodegradable scaffolds/cements are very limited in use because of poor mechanical stability, and limited promotion of vascularization. Composites of CaPs with biodegradable metals, e.g., magnesium, and incorporation of GFs could solve this intricate problem and increase the use of CaP products in reconstructive surgery. However, as described in Section 9, the inclusion of GFs is not in favour nowadays due to health safety issues.
(6) *Bone/cartilage, bone mineral/collagen and bone/tendon interfaces*. While many efforts are focused on the issues described above, not enough attention is focused on bone interfaces. A better understanding of the biological systems is needed. For example, the bonding mechanism between the bone mineral and collagen remains unclear [854]. It is also unclear whether a rapid repair that is elicited by the new generation of bioceramics results from the enhancement of mineralization *per se* or whether there is a more complex signalling process involving proteins in collagen. If we were able to understand the fundamentals of bone response to specific ions and the signals they activate, then we could design better bioceramics for the future [854]. From application standpoint, CaP-based FGMs may very well address these issues and become the golden standard in CaP implants.
(7) *Transient precursor phases*. As described in Section 3, ACP, DCPD and OCP have been suggested as transient precursor phases. However, there is yet no consensus in the scientific community regarding the prevalence of these phases and the exact mechanisms of biomineralization relevant to human bone remodelling. High-resolution, in situ structural and chemical studies of human bone formation and remodelling may be possible one day and clarify this old scientific debate. The outcomes of such studies may aid in developing better bone substitutes and CaP-based coatings.

According to Anderson [855], since 2000 the focus of biomaterials has been on the *bio* side. For CaPs this means a shift of the focus from osteoconduction to osteoinduction, e.g., by fabrication of scaffolds with controlled three-dimensional porous structures and development of novel ion-substituted CaPs with increased biological activity. In the future, the composition, microstructure and molecular surface chemistry of various types of CaPs will be tailored to match the specific biological and metabolic requirements of tissues or disease states [854,856].

New strategies, possibly based on self-assembling and/or nanofabrication [857], have to be developed for successful fabrication of load-bearing bone graft substitutes [854]. In addition, a new generation of gene-activating CaP-based scaffolds tailored for specific patients and disease states may be developed in the future. CaPs may become more common for carrying nucleic acids (DNA or RNA) into nuclei of living cells for gene therapy. Possibly, bioactive stimuli will be used to activate genes in a preventative treatment to maintain the health of aging tissues [854]. We believe that this area has great development potential.

Wegst et al. [88] discussed future directions in bioinspired materials. Additive manufacturing (AM) was proposed as an ideal way to assemble, on demand, structures modelled after natural materials. However, in order for this to happen, several challenges have to be overcome first. First, the range of materials that can be processed by AM ought to be broadened. The high thermal stability of ceramics in part hinders the use of techniques that involve melting or in situ sintering. Therefore, most ceramic AM technologies require an ‘ink’, typically a colloidal suspension in water or other solvent, or a wax containing ceramic particles. Furthermore, the parts usually require additional thermal treatments for consolidation. A further complication is that bioinspired materials are usually hybrids that combine dissimilar materials (for example, a CaP ceramic and a collagen polymer)—something that may be difficult to construct using a single technique [88]. Second, it is necessary to combine the precision required to print nanoscale features with the fabrication of large-scale components. This combination is currently not feasible by any existing technology. For example, continuous extrusion of sol-gel ceramic inks, two-photon polymerization (nanolithography) and inkjet printing allow construction of materials with fine features, but not large structures. In contrast, technologies such as robocasting using colloidal ceramic inks, 3D printing, or stereolithography can allow large-scale manufacturing, yet their ultimate feature resolution is of the order of tens or hundreds of micrometres. Despite these difficulties, there has been some progress [88]. Monolithic, multilayer, and chemically gradient CaP coatings have been cladded on various metal substrates relevant to implants by means of the Optomec’s LENS™ directed energy deposition AM process [563,858,859,860,861].

**Figure 15:**
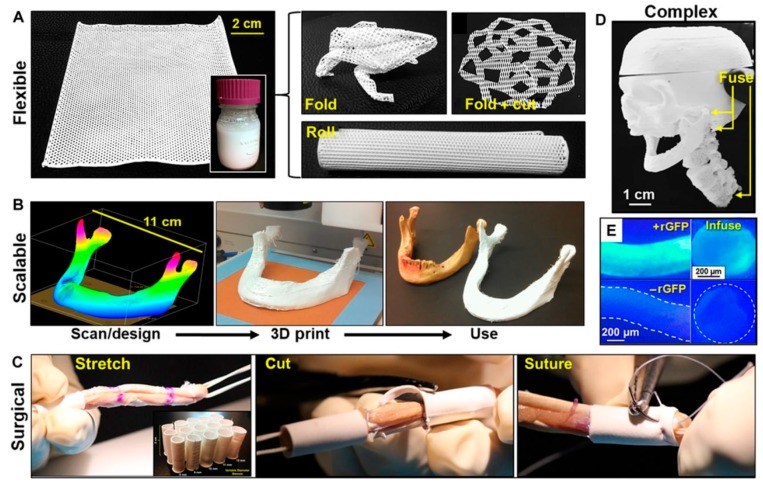
Versatility, scalability, and manipulation of 3D-printed hyperelastic “bone” (HB). (**A**) Easy to synthesize volumes (~100 mL shown) of liquid-based HB inks (inset) can be 3D-printed into a variety of structures: 3D-printed 12 × 12 cm HAp-poly(d,l-lactic-*co*-glycolide acid) (PLGA) sheet comprising three layers, which can be manipulated in a variety of ways, including rolling, folding, and cutting. Origami methods may be used to create complex folded structures, whereas Kirigami methods can produce complex structures from strategic folding and cutting; (**B**) Full-scale, anatomically correct parts, such as a human mandible, comprising >250 layers, can be designed, 3D-printed from HAp-PLGA, and washed to rapidly produce a ready-to-implant object. Final image shows 3D-printed mandible next to an adult cadaveric human mandible; (**C**) Photograph series illustrating that custom-sized HAp-PLGA sleeves can be snuggly stretched around, cut, and sutured to a soft tissue, such as human cadaveric tendon, facilitating arthroscopic ACL repair and replacement surgery; (**D**) Independently 3D-printed HAp-PLGA miniature-scale versions of a human skull, skull cap, mandible, and upper thoracic seamlessly fused together to create highly complex structures by using HB ink applied to points of contact; (**E**) Black light-illuminated optical photographs of the outside and internal cross-sections of HAp-PLGA fibre with (top) and without (bottom) incorporated recombinant green fluorescent protein (rGFP) [862]. Reproduced with permission from The American Association for the Advancement of Science.

Recently, Jakus et al. [862] manufactured a novel, synthetic osteoregenerative biomaterial, which they called hyperelastic “bone” (HB). This materials, which consists of 90 wt % HAp and 10 wt % PCL or PLGA, could be rapidly 3D-printed (up to 275 cm^3^/h) at room temperature from extruded liquid inks. The resulting 3D-printed HB exhibited elastic mechanical properties, was highly absorbent, supported cell viability and proliferation *in vitro*, and induced osteogenic differentiation of bone marrow-derived hMSCs without any osteoinducing factors in the medium. *In vivo*, HB did not elicit a negative immune response, became vascularized, quickly integrated with surrounding tissues, and rapidly ossified and supported new bone growth without the need for added biological factors. 3D-printed solid HB structures comprising many hundreds of layers were prepared (Figure 15A, inset). The resulting structures did not require further postprinting processing other than rinsing and sterilization before use, and exhibited mechanical and physical properties that permitted further manipulation. For example, a 3D-printed HAp-PLGA sheet could be rolled, folded, and cut (Figure 15A) to create architectures that might otherwise not be possible to 3D-print directly because of the large, unsupported overhangs. An example of how HB could be used surgically is illustrated in Figure 15C, where HB cylinders of various sizes were 3D-printed (inset), and the correct size was selected. Jakus et al. also rapidly 3D-printed HB inks into anatomically scaled, patient-specific grafts, such as an adult human mandible (Figure 15B). Additionally, unlike many other 3D-printable materials, HB inks could be used as self-adhesives, allowing independently 3D-printed objects made of the same or similar materials to be seamlessly fused together. Individually 3D-printed components were merged to form highly complex geometries, which would be impossible to 3D-print as one monolithic object (Figure 15D). Furthermore, HB inks were used as flexible coatings on other implantable materials, such as metallic screws. Last, the ability to synthesize and 3D-print HB inks under ambient conditions with no need for further sintering or chemical cementation allowed incorporation of biological factors and molecules, such as proteins (Figure 15E), peptides, genes, and antibiotics, which may enhance tissue regeneration and reduce infection [862].

**Figure 16:**
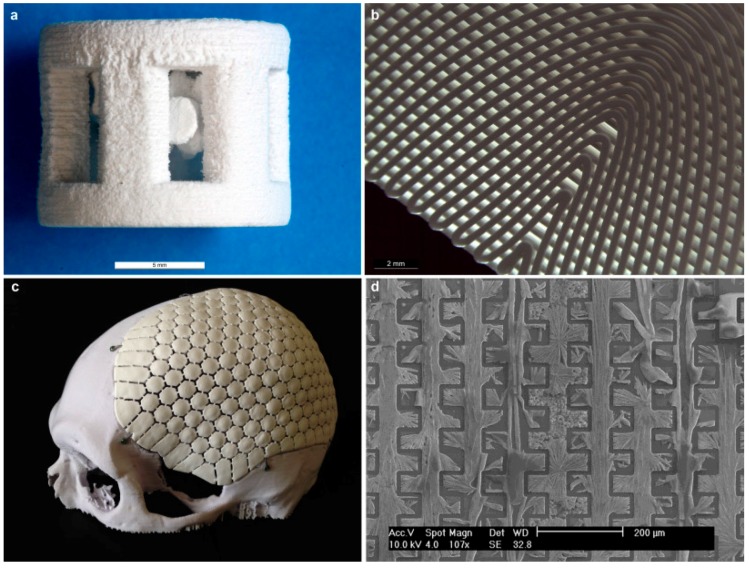
Examples of structures obtained by additive manufacturing techniques. (**a**) 3D printed scaffolds made of dicalcium phosphate anhydrous (DCPA) (scale bar: 5 mm) [864]; (**b**) Solid obtained by robocasting (scale bar: 2 mm); (**c**) “Craniomosaic”: a DCPA-based implant for treatment of cranial bone defects. The device uses a 3D-printed titanium mesh covered with DCPA ceramic tiles; (**d**) Pattern of CaP created on a silicon substrate using soft lithography (scale bar: 200 μm) [863]. Reproduced with permission from Elsevier Ltd.

Habraken et al. [863] reviewed several AM technologies for manufacturing of bone substitutes. Both macropores (larger than 100 μm) and micropores (0.1–10 μm) were found essential to provide fast resorption. Printing hollow structures is strongly limited by the need to remove the powder from unprinted volumes (depowdering, see Figure 16a) [864]. Also, it is not easy to perfectly control the composition of the printed pieces, and post-treatments in acids are generally required [863]. Another AM technique called ‘robocasting’ is based on the extrusion of a thick CaP slurry through a thin nozzle [865] (Figure 16b). Several companies have started commercializing products based on AM, for example, scaffolds with an oriented architecture to promote bone ingrowth in a specific direction [866], or innovative craniofacial implants combining a 3D-printed titanium mesh and DCPA ceramic tiles (Figure 16c) [863]. The application of patterning techniques of chemical or structural cues to obtain spatial and/or temporal control over a biological response is also likely to expand in the years to come (Figure 16d) [863].

## 17. Conclusions

Calcium phosphate (CaP) bioceramics are widely used in the field of bone regeneration, both in orthopedics and in dentistry, due to their good biocompatibility, osseointegration and osteoconduction. This article reviews many aspects of CaP bioceramics, in the form of bone cements, paste, scaffolds, and coatings. It starts with a brief historical perspective of their development and uses. Next, it describes the structure, chemistry and mechanical properties of bone. Then, it describes the transient precursor phases that have been observed, both *in vitro* and *in vivo*, and the mechanisms of dissolution of CaPs and their reprecipitation as bone. Next, the key requirements from CaPs for medical applications are presented. The structure, properties and applications of CaP-based materials are reviewed, starting with the individual phases as a function of their Ca-to-P atomic ratio, then nano-CaP, biphasic and triphasic formulations, composites and functionally graded materials (FGMs). The antibacterial behaviour of CaPs thanks to incorporation of certain antibacterial agents and the effect of different sterilization processes on CaPs are reviewed. Next, key principles of *in vitro* and *in vivo* tests are described. A variety of technologies for coating CaPs are reviewed, focusing on electrochemical processes and the requirements of the US FDA and international standards. Next, a variety of commercial products is listed according to their composition, and major applications are visualized. Finally, key issues in future developments are listed, along with recent developments in additive manufacturing (AM) of CaPs and hyperelastic bone.

## Figures and Tables

**Table 1:** Chemical composition of bone (wt %) [67,121].

Inorganic Phase	Organic Phase
HAp ≈ 60	Collagen ≈ 20
H_2_O ≈ 9	Non-collagenous proteins (osteocalcin, osteonectin, osteopontin, thrombospondin, morphogenetic proteins, sialoprotein, serum proteins) ≈ 3
Carbonate ≈ 4	Traces: Polysaccharides, lipids, cytokines
Citrate ≈ 0.9	Primary bone cells: osteoblasts, osteocytes, osteoclasts
Na^+^ ≈ 0.7	-
Mg^2+^ ≈ 0.5	
Cl^−^ ≈ 0.13	
Others: K^+^, F^−^, Zn^2+^, Fe^2+^, Cu^2+^, Sr^2+^, Pb^2+^	

**Table 2:** Properties required from calcium phosphates for medical applications.

Property	Definition/Function
Bioactivity	The inherent ability of a material to participate in specific biological reactions or have an effect on living tissues
Biocompatibility	The ability of a material to perform with an appropriate host response in a specific application
Bioactive fixation	Reactive surfaces form chemical bonding with bone, thus minimizing the fibrous capsule formation
Biostability	The ability of a material to maintain its properties *in vivo*
Crystallinity	Higher level of crystallinity prevents fast resorption (dissolution) of the bioceramic in body fluids
Interfacial stability and good adhesion	Prevent mechanical failures under load-bearing conditions
Osseointegration	Direct anchorage of an implant by the formation of bony tissue around it without growth of fibrous tissue at the bone/implant interface
Osteoconduction	Ability to provide a scaffold for the formation of new bone
Osteoinduction	The process by which osteogenesis is induced. This term means that primitive, undifferentiated and pluripotent cells are somehow stimulated to develop into the bone-forming cell lineage
Resorption	Gradual degradation over time to replace the biomaterial with the natural host tissue
Therapeutic capabilities	Templates for the in situ delivery of drugs and growth factors at required times
Wettability	The property that indicates a material’s ability to attract/repel water molecules

**Table 3:** Selected CaP phases of interest for biomedical applications [49,72,77,110,229].

Ca/P Molar Ratio	Name	Formula	pH Stability Range	Density (g/cm^3^)
0.5	MCPM (monobasic calcium phosphate monohydrate)	Ca(H_2_PO_4_)_2_·H_2_O	0.0–2.0	2.22
1.0	DCPA (dicalcium phosphate anhydrous, Monetite)	CaHPO_4_	2.0–5.5 (>80 °C)	2.929
1.0	DCPD (dibasic calcium phosphate dehydrate, Brushite)	CaHPO_4_·2H_2_O	2.0–6.0	2.319
1.33	OCP (octacalcium phosphate)	Ca_8_(HPO_4_)_2_(PO_4_)_4_·5H_2_O	5.5–7.0	2.673
1.5	α-TCP (α-tricalcium phosphate)	α-Ca_3_(PO_4_)_2_	Precipitated from aqueous solutions only at *T* > 1125 °C	2.814 ^3^
1.5	β-TCP (β-ticalcium phosphate)	β-Ca_3_(PO_4_)_2_	Precipitated from aqueous solutions only at *T* > 800 °C	3.067 ^3^
1.2–2.2	ACP (amorphous calcium phosphate)	Ca*_x_*H*_y_*(PO_4_)*_z_*·*n*H_2_O, *n* = 3–4.5, 15%–20% H_2_O	~5–12 ^1^	-
1.50–1.67	CDHA (calcium deficient hydroxyapatite, CDHAp; precipitated HAp, pHA, pHAp)	Ca_10−*x*_(HPO_4_)*_x_*(PO_4_)_6−*x*_(OH)_2−*x*_ (0 < *x* < 2) ^2^	6.5–9.5	-
1.67	HAp, or OHAp (Hydroxyapatite)	Ca_10_(PO_4_)_6_(OH)_2_	9.5–12.0	3.155
2.0	TTCP, or TetCP (tetracalcium phosphate, Hilgenstockite)	Ca_4_(PO_4_)_2_O	Precipitated from aqueous solutions only at *T* > 1300 °C	3.056 ^3^

^1^ Always metastable. The composition of the precipitate depends on the composition and pH of the electrolyte solution; ^2^ In the case *x* = 1 (the boundary condition with Ca/P = 1.5), the chemical formula looks as follows: Ca_9_(HPO_4_)(PO_4_)_5_(OH); ^3^ These compounds cannot be precipitated from aqueous solutions.

**Table 4:** Solubility of selected CaP phases of interest for biomedical applications [49,72,77,110,229,249].

Name	Solubility at 37 °C, −log(*K*_s_)	Solubility at 25 °C, −log(*K*_s_)	Solubility at 25 °C, g/L
MCPM	--	1.14	~18
DCPA	7.02	6.90	~0.048
DCPD	6.63	6.59	~0.088
OCP	95.9	96.6	~0.0081
α-TCP	25.5	25.5	~0.0025
β-TCP	29.5	28.9	~0.0005
ACP	^1^	^1^	-
CDHA	~85.1	85.1	~0.0094
HAp	117.2	116.8	~0.0003
TTCP	37–42	38–44	~0.0007

^1^ Cannot be measured precisely. However, the following values were found: 25.7 ± 0.1 (pH = 7.40), 29.9 ± 0.1 (pH = 6.00), 32.7 ± 0.1 (pH = 5.28). The comparative solubility in acidic buffer decreases in the following order: ACP >> α-TCP >> β-TCP > CDHAp >> HAp.

**Table 5:** Crystallographic data on selected CaP phases.

Name	Space Group	Unit Cell Parameters	JCPDS ^1,2^ File
MCPM	Triclinic $P\bar{1}$	*a* = 6.250, *b* = 11.892, *c* = 5.629 Å α = 96.67°, β = 114.20°, γ = 92.95°	00-009-0347
DCPA	Triclinic $P\bar{1}$	*a* = 6.910, *b* = 6.627, *c* = 6.998 Å α = 96.34°, β = 103.82°, γ = 88.33°	00-003-0398, 00-004-0513, 00-009-0080, 01-075-1520, 04-009-3755, 04-009-6216, 04-011-3070
DCPD	Monoclinic *Ia*	*a* = 5.812, *b* = 15.180, *c* = 6.239 Å β = 116.42°	00-009-0077, 00-011-0293, 04-008-2081, 04-013-3344
OCP	Triclinic $P\bar{1}$	*a* = 9.529, *b* = 18.994, *c* = 6.855 Å α = 92.33°, β = 90.13°, γ = 79.93°	00-026-1056, 00-044-0778, 04-013-3883, 04-016-3473
α-TCP	Orthorhombic (Monoclinic *P*2_1_/*a*?)	*a* = 15.220, *b* = 20.710, *c* = 9.109 Å α = β = γ = 90°	00-009-0348, 00-029-0359
β-TCP	Rhombohedral *R*3*c*	*a* = *b* = 10.439, *c* = 37.375 Å α = β = 90.00°, γ = 120.00°	00-009-0169, 04-002-4776, 04-008-8714
CDHA	Hexagonal *P*6_3_/*m*	*a* = *b* = 9.4157–9.4490, *c* = 6.8777–6.8865 Å α = β = 90°, γ = 120°	00-046-0905
HAp	Monoclinic *P*2_1_/*b* or Hexagonal *P*6_3_/*m* ^3^	*a* = 9.84214, *b* = 2*a*, *c* = 6.8814 Å, γ = 120° (monoclinic). *a* = *b* = 9.418, *c* = 6.884 Å α = β = 90.00°, γ = 120.00° (hexagonal)	00-009-0432, 00-024-0033, 01-074-0565, 01-074-0566, 01-084-1998, 01-089-4405, 04-007-2837, 04-007-5086, 04-016-1185
TTCP	Monoclinic *P*2_1_	*a* = 7.018, *b* = 11.980, *c* = 9.469 Å, α = γ = 90.00°, β = 90.88°	00-011-0232, 00-025-1137

^1^ JCPDS: Joint Committee on Powder Diffraction Standards; ^2^ In bold: Our most recommended file for use in experimental studies; ^3^ The stoichiometric HAp is monoclinic at temperatures <212 °C. However, small quantities of impurities lead to a transformation from monoclinic to hexagonal, the only form found in biology.

**Table 6:** Physicochemical, mechanical and biological properties of HAp [121,374,383,384,385].

Property	Value	Comments
Binding energy	−280.6 eV	Reference [373]
Kohn-Sham gap	−5.4 eV	Reference [373]
Dielectric constant	7.40–10.47	-
Thermal conductivity	0.013 W/(cm·K)	-
Relative density	95%–99.5%	-
Decomposition temperature	>1000 °C	-
Melting point	1614 °C	-
Tensile strength	38–300 MPa ~3 MPa	For dense HAp For porous HAp
Compressive strength	120–900 MPa 2–100 MPa	For dense HAp For porous HAp
Bending strength	38–250 MPa 2–11 MPa	For dense HAp For porous HAp
Young’s (elastic) modulus	35–120 GPa	For dense HAp
Fracture toughness	0.7–1.2 MPa·m^1/2^	Decreases almost linearly with porosity [383]
Fracture energy	2.3–20 J/m^2^	Behaves like a typical brittle ceramic
Vickers hardness	3–7 GPa	For dense HAp
Poisson’s ratio	0.27	For synthetic HAp (bones ~0.3)
Biocompatibility	High	-
Bioactivity	High	-
Biodegradation	Low	-
Cellular compatibility	High	-
Osteoinduction	Nil	-
Osteoconduction	High	-

**Table 7:** Ion concentrations (mM) in simulated body fluids (SBFs), human blood plasma and synovial fluid.

Medium	Na^+^	K^+^	Ca^2+^	Mg^2+^	Cl^−^	$HCO_{3}^{-}$	$HPO_{4}^{2-}$	$SO_{4}^{2-}$	Organic Acids (mg/L)	Proteins (mg/L)
Human blood plasma	142.0	3.6–5.5	2.1–2.6	1.5	95.0–107.0	27.0	0.65–1.45	0.5	210	66,300
Synovial fluid	136.0	4.0	1.5	-	107.5	30.8	1.0	0.5	-	15,000
Original SBF	142.0	5.0	2.5	1.5	148.8	4.2	1.0	-	-	-
Corrected SBF (c-SBF)	142.0	5.0	2.5	1.5	147.8	4.2	1.0	0.5	-	-
Revised SBF (r-SBF)	142.0	5.0	2.5	1.5	103.0	27.0	1.0	0.5	-	-
Newly improved SBF (n-SBF)	142.0	5.0	2.5	1.5	103.0	4.2	1.0	0.5	-	-
Phosphate-buffered saline (PBS)	157.0	4.5	-	-	139.7	-	10.0 + 1.8 $PO_{4}^{3-}$	-	-	-
Ringer’s	291.3	10.8	6.3	-	212.0	3.3	-	-	-	-
Hanks’ balanced salts solution (HBSS)	141.7	5.7	1.7	0.8	145.6	4.2	0.7	0.8	-	-

**Table 8:** Requirements from CaP coatings for orthopedic and dental implants [806,808].

Property	Requirement	Testing Method	Standard	Comment
Chemical composition	Ca/P = 1.67–1.76	Inductively coupled plasma/mass spectroscopy (ICP/MS) or atomic absorption (AAS)	ISO 13779-3 ASTM F1088 ASTM F1185	Atomic ratio. Analyse enough samples to produce a meaningful mean and variance (i.e., 95% confidence interval)
Trace elements	As: max 3 ppm, Cd: max 5 ppm, Hg: max 5 ppm, Pb: max 30 ppm, Total heavy metals: 50 ppm	Inductively coupled plasma/mass spectroscopy (ICP/MS) or atomic absorption (AAS)	ISO 13779-3 ISO 10993-17 ASTM F1185	-
Phase content	Only HAp and OCP	XRD + FTIR to identify functional groups	ASTM F2024 ISO 13779-3	XPS analysis: [185]. Superimpose relevant JCPDS/ICDD lines. Provide a table identifying all peaks by intensity, *d*-spacing and 2θ. Specify scan range and scan rate. Report preferred orientations, effect of strain, etc. Characteristic absorption bands for HAp: 570, 962 and 1050 cm^−1^ for $PO_{4}^{3-}$; 630 and 3540 cm^−1^ for $OH^{-}$
Percentage of crystallinity	min 45% crystalline HAp, max 5% other crystalline phases, Balance: amorphous	-	ISO 13779-3	-
Adhesion strength	min 15 MPa (under tension)	-	ISO 13779-4 ASTM F1147 in tension. ASTM F1044 shear adhesive strength	Provide SEM images at 100× of the epoxy/coating/substrate prior to testing to demonstrate any potential penetration of the epoxy. At least 10 samples. Report STDEV
Microporosity and macroporosity	-	-	ASTM F1854	Report average porosity size and overall pore volume
Surface coverage	-	Microscopic examination at 10× magnification. SEM images at 100× of the coated implant surfaces and of a cross-sectioned area of the implant showing the coating interface	-	Report “bare” areas, “pinholes,” cracking, foreign debris, unmelts, chips, delamination, the appearance at the coating/substrate interface, etc. Provide photomicrographs at 100×
Coating thickness	-	Cross-sections	ASTM F1854 ASTM E376	Report distinct layers, if exist, and tolerance
Colour	-	Macroscopic examination	-	Ensure a uniform and consistent appearance
Surface roughness	-	-	ANSI/ASME B46.1	*R*_a_ and the tolerances of the substrate and coating should be reported
Abrasion resistance	-	-	None available for CaP. ASTM F1978 may be used	Need to determine if the coating will spall.
Fatigue strength	-	Three-point bending, rotating beam, or modified static test methods for testing of dental implants	ASTM F1160. ISO 7206 for hip prostheses	Both the coating/substrate interface and the effect on the substrate should be evaluated. The effect of the coating on the resulting fatigue strength of an actual implant should also be considered. Provide SEM images of failure regions. Provide S/N curve. Test for the worst-case scenario. For femoral stems, the S/N curve may be substituted with testing of the stem at a load of 3–4 times body weight and *R* = 0.1 for 10 M cycles. A sample size of 5 is required
Solubility products and dissolution rate	-	In a physiologically similar solution such as tris-HCl buffered solution at 37 °C and pH = 3.0 and 7.3	ASTM F1926	Measurement should include dissolved Ca and P. Weight loss should also be measured. If compound contains other elements such as F, these should be measured too. Monitor pH changes. Calculate *K*_sp_
Density of the coating	2.98 g/cm^3^	Helium pycnometer	-	-
Animal studies	-	-	-	Check Guidance for the Arrangement and Content of a Premarket Approval (PMA) Application for an Endosseous Implant for Prosthetic Attachment
Clinical studies	-	-	-	Check Guidance for the Arrangement and Content of a Premarket Approval (PMA) Application for an Endosseous Implant for Prosthetic Attachment

**Table 9:** Trademarks of the commercially produced CaPs.

Composition	Product Name	Producer
β-TCP	adbone^®^TCP	Medbone, Portugal
	Bioresorb	Sybron Implant Solutions, Germany
	Biosorb	SBM S.A., France
	Calciresorb	Ceraver, France
	Cerasorb	Curasan, Germany
	Ceros	Thommen Medical, Switzerland
	Conduit	DePuy Spine, USA
	JAX	Smith and Nephew Orthopaedics, USA
	Osferion	Olympus Terumo Biomaterials, Japan
	OsSatura TCP	Integra Orthobiologics, CA, USA
	SynthoGraft	Synthograft, MA, USA
	Triha+	Teknimed, France
	Vitoss	Orthovita, PA, USA
CDHA	Osteogen	Impladent, NY, USA
HAp	Actifuse	ApaTech, UK
	Apaceram	Pentax, Japan ApaTech, UK
	ApaPore	ApaTech, UK
	Bioroc	Depuy-Bioland, France
	Bonefil	Pentax, Japan
	Bonetite	Pentax, Japan
	Boneceram	Sumitomo Osaka Cement, Japan
	Bone Source	Stryker Orthopaedics, NJ, USA
	Calcitite	Zimmer, IN, USA
	Cerapatite	Ceraver, France
	Neobone	Toshiba Ceramics, Japan
	Ostegraf	Ceramed, CO, USA
	Ostim	Heraeus Kulzer, Germany
	Synatite	SBM, France
Coralline HAp	Interpore	Interpore, CA, USA
	ProOsteon	Interpore, CA, USA
Algae-derived HAp	Algipore	Dentsply Friadent, Germany
Bovine bone apatite (unsintered)	BioOss	Geitslich, Switzerland
	Laddec	Ost-Developpement, France
	Lubboc	Ost-Developpement, France
	Oxbone	Bioland biomateriaux, France
	Tutoplast	IOP, CA, USA
Bovine bone apatite (sintered)	Cerabone	aap Implantate, Germany
	Endobon	Biomet Deutschland GmbH, Germany
	Osteograf	Ceramed, CO, USA
	PepGen P-15	Dentsply Friadent, Germany
	XenoGraft	Staumann, Switzerland
HAp + collagen	Bioimplant	Connectbiopharm, Russia
	Bonject	Koken, Japan
	CollapAn	Intermedapatite, Russia
	HAPCOL	Polystom, Russia
	Healos Fx	DePuy Spine, USA
	LitAr	LitAr, Russia
HAp + sodium alginate	Bialgin	Biomed, Russia
HAp + PLLA	SuperFIXSORB30	Takiron, Japan
HAp + Polyethylene	HAPEX	Gyrus, TN, USA
HAp + CaSO_4_	Hapset	LifeCore, MIN, USA
BCP (HAp + β-TCP)	4Bone	MIS, Israel
	BCP	Medtronic, MN, USA
	Biosel	Depuy Bioland, France
	BoneSave	Stryker Orthopaedics, NJ, USA
	Calciresorb	Ceraver, France
	CellCeram	Scaffdex, Finland
	Ceraform	Teknimed, France
	Ceratite	NGK Spark Plug, Japan
	Eurocer	FH Orthopedics, France
	Graftys BCP	Graftys, France
	Hatric	Arthrex, Naples, FL, USA
	Indost	Polystom, Russia
	MBCP Gel In’Oss (contains also hydrogel)	Biomatlante, France
	Kainos	Signus, Germany
	Mastergraft	Medtronic, IN, USA
	Maxresorb	Staumann, Switzerland
	MBCP	Biomatlante, France
	OptiMX	Exactech, FL, USA
	OsSatura BCP	Integra Orthobiologics, CA, USA
	Osteosynt	Einco, Brazil
	Repros	JRI Orthopaedics, UK
	SBS	Expanscience, France
	TCH	Kasios, France
	Triosite	Zimmer, IN, USA
	Tribone	Stryker, Europe
	Valeos	Innov’spine, France
BCP (HAp + α-TCP)	Skelite	Millennium Biologix, ON, Canada
BCP (DCPD/HAp)	BONIT	DOT Medical Implants Solutions, Germany
	BONITex	DOT Medical Implants Solutions, Germany
BCP + collagen	Allograft	Zimmer, IN, USA
BCP + fibrin	TricOS	Baxter BioScience, France
BCP + silicon	FlexHA	Xomed, FL, USA
FA + BCP (HAp + β-TCP)	FtAP	Polystom, Russia
β-TCP + PMMA	Cal-CEMEX	Tecres Spa, Italy
rhBMP-2 on the surface of HAp/β-TCP	CowellBMP	Cowellmedi Co (CWM), Korea
TTCP + DCPA + saline	BoneSource HAC	Stryker Instruments, MI, USA
α-TCP + TTCP + sodium glycerophosphate + (lime + phosphoric acid)	Cementek	Teknimed LC, France
CaP within lyophilized type I bovine collagen sponges	CopiOS Sponge	Zimmer Biomet Spine, CO, USA
ACP + DCPD	Biobon (α-BSM)	Etex, MA, USA
BCP (HAp + β-TCP) granules, bovine collagen and bone marrow aspirate	Collagraft	Zimmer, IN, USA
β-TCP granules and polymer	Therigraft Putty	Therics, OH, USA
β-TCP granules and an aqueous solution of glycerol and carboxymethylcellulose (CMC)	JAXTCP	Smith and Nephew, USA
HAp, P-15 peptide and aqueous Na-hyaluronate solution	Pepgen P-15 flow	Dentsply, PA, USA
α-TCP + TTCP + CaHPO_4_ + HAp	BIOPEX	Taisho Pharmaceutical, Japan
BCP (DCPD + β-TCP)	ChronOS	DePuy Synthes, PA, USA
Carbonate apatite	Healos	Orquest, CA, USA
	Norian SRS	Synthes, PA, USA
